# Supplementary material for: Interplay between c-Src and the APC/C co-activator Cdh1 regulates mammary tumorigenesis
Source: Nat Commun. 2019 Aug 16;10:3716. doi: 10.1038/s41467-019-11618-7 (PMC6697746; doi:10.1038/s41467-019-11618-7)
Supplement: Supplementary file 1 — Supplementary Information [file 41467_2019_11618_MOESM1_ESM.pdf]

Supplementary Information

Interplay between c-Src and the APC/C Co-activator Cdh1 Regulates Mammary Tumorigenesis

Han et al.

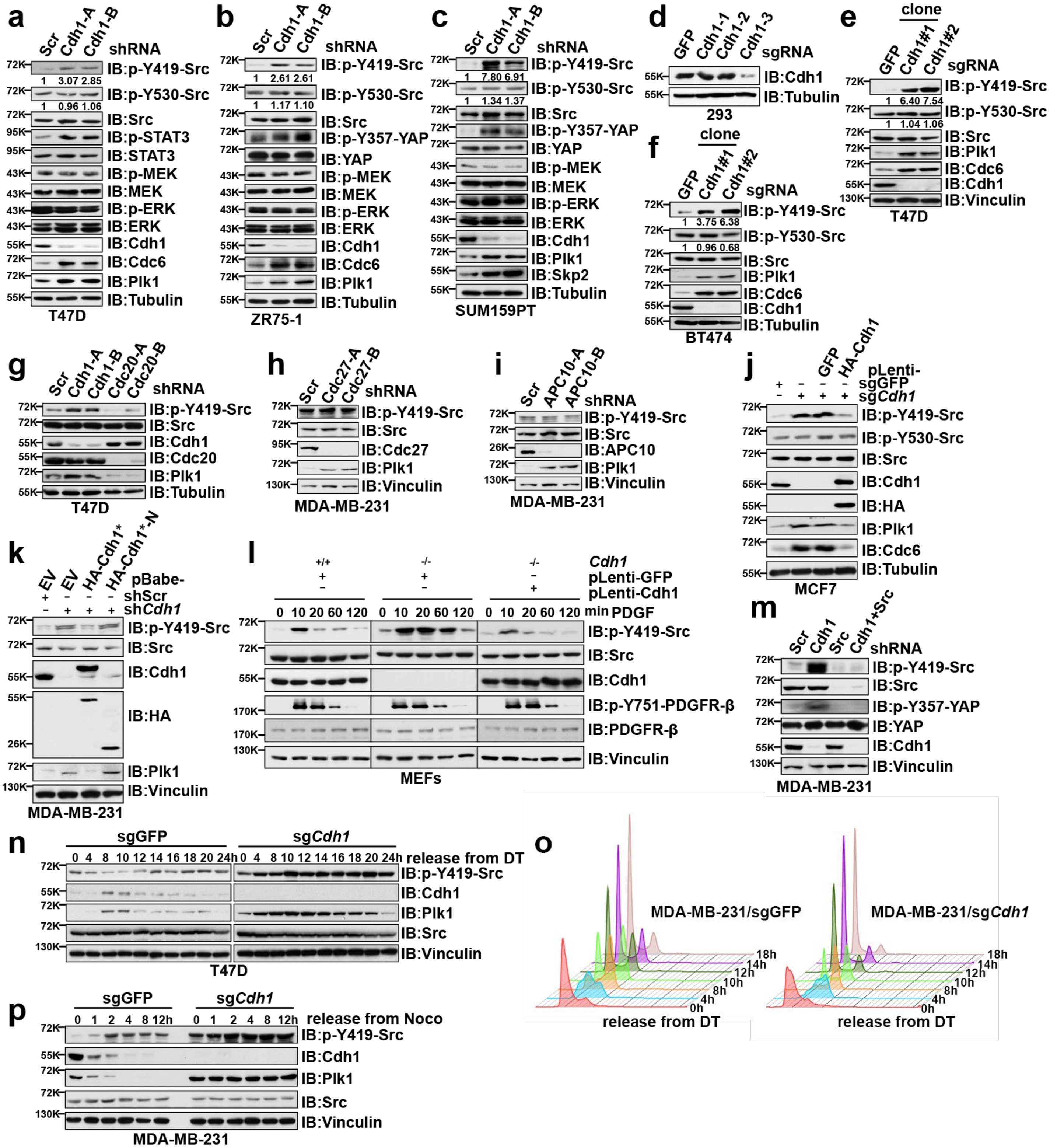

Supplementary Figure 1 Cdh1 negatively regulates Src kinase activity in an APC-independent manner.

(a-c) Immunoblot (IB) analysis of T47D (**a**), ZR75-1 (**b**) and SUM159PT (**c**) cells infected with control (shScr) or the indicated sh*Cdh1* lentiviral shRNA constructs. The infected cells were selected with 1 μg·ml^{-1} puromycin for 72 h before harvest.

(d) IB analysis of HEK293 cells infected with control (sgGFP) or the indicated sg*Cdh1* construct. The infected cells were selected with 1 μg·ml^{-1} puromycin for 7 days before harvest.

(e-f) IB analysis of T47D (**e**) and BT474 (**f**) cells infected with control (sgGFP) or sg*Cdh1* lentiviral construct. The infected cells were selected with 1 μg·ml^{-1} puromycin for 7 days before plating for single clone selection.

(g) IB analysis of T47D cells infected with the indicated lentiviral shRNA constructs. The infected cells were selected with 1 $\mu g{\cdot}ml^{-1}$ puromycin for 72 h before harvest.

(h-i) IB analysis of MDA-MB-231 cells infected with control (shScr) or the indicated sh*Cdc27* (**h**) and sh*APC10* (**i**) lentiviral shRNA constructs. The infected cells were selected with 1 $\mu g{\cdot}ml^{-1}$ puromycin for 72 h before harvest.

(j) sgGFP and sg*Cdh1*-MCF7 cells stably expressing lentiviral GFP or Cdh1 constructs as indicated were selected with 100 $\mu g{\cdot}ml^{-1}$ hygromycin for 72 h before harvest.

(k) MDA-MB-231 cells stably expressing EV, WT-, or *N*-Cdh1 retroviral constructs were further infected with shScr or sh*Cdh1* lentiviral constructs as indicated. The infected cells were selected with 1 $\mu g{\cdot}ml^{-1}$ puromycin for 72 h before harvest. *Cdh1 cDNA used in this experiment has been mutated to escape sh*Cdh1*-mediated gene silencing.

(l) WT and *Cdh1*$^{-/-}$ MEFs were infected with lentiviral GFP or Cdh1 constructs as indicated were selected with 100 $\mu g{\cdot}ml^{-1}$ hygromycin for 72 h before harvest.

(m) IB analysis of MDA-MB-231 cells infected with the indicated lentiviral shRNA constructs. The infected cells were selected with 1 $\mu g{\cdot}ml^{-1}$ puromycin and 100 $\mu g{\cdot}ml^{-1}$ hygromycin for 72 h before harvest.

(n) IB analysis of WCL derived from sgGFP- and sg*Cdh1*-infected T47D cells that were synchronized at the G1-S boundary by double-thymidine block and then released back into the cell cycle for the indicated periods of time.

(o) Flow cytometric analyses showed the cell cycle profiles of the synchronized cells derived from **Fig. 1k.**

(p) IB analysis of WCL derived from sgGFP- and sg*Cdh1*-infected MDA-MB-231 cells that were synchronized in M phase by 300 nM nocodazole arrest and then released back into the cell cycle for the indicated periods of time.

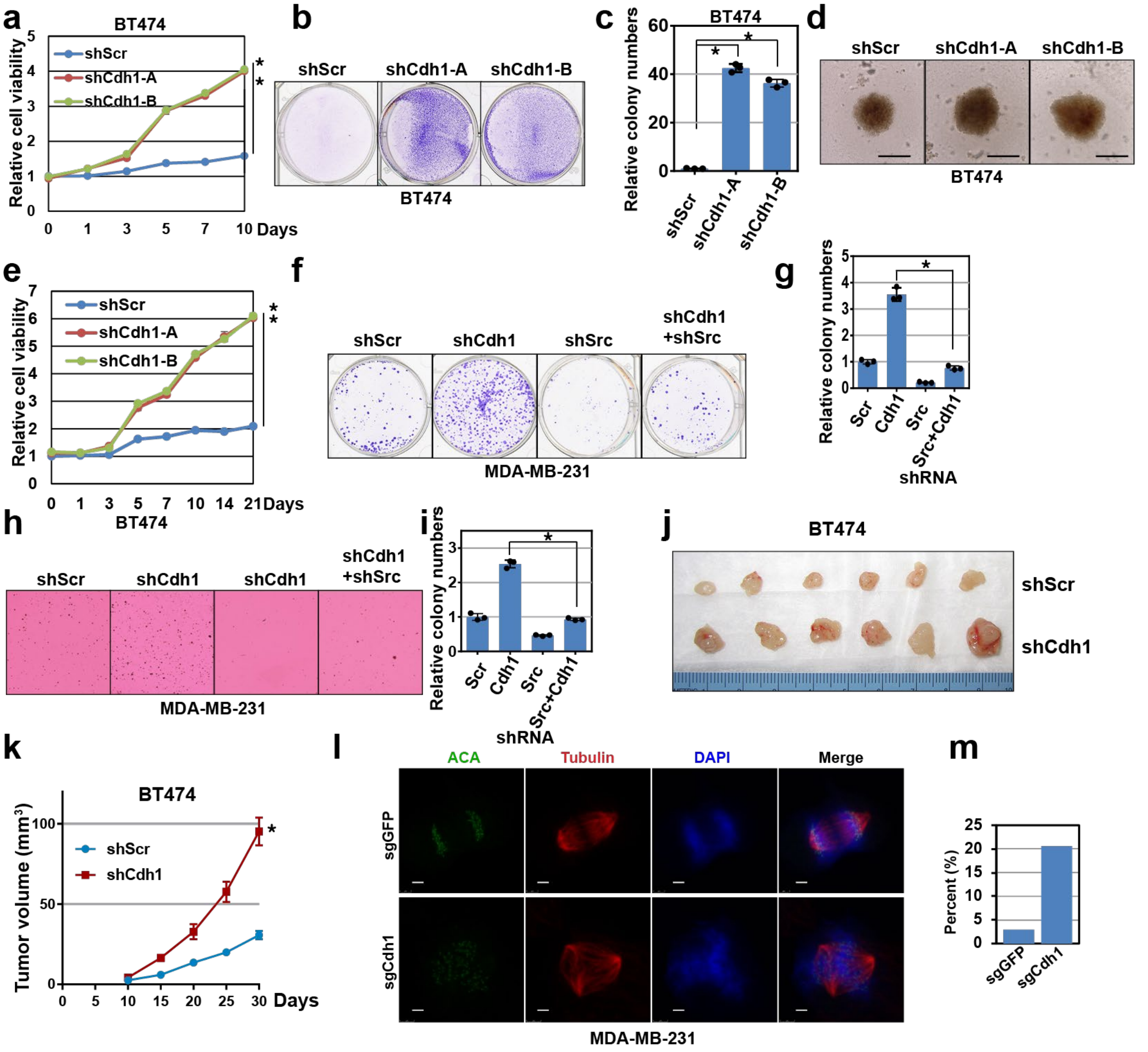

Supplementary Figure 2 Depletion of *Cdh1* augments breast cancer cell proliferation.

(a) BT474 cells infected with control shRNA (shScr) or sh*Cdh1* lentiviral constructs as described in Figure 1c were subjected to cell proliferation assays in RPMI1640 medium supplemented with 10% FBS for up to 10 days. Relative cell viability was determined at the indicated time points and was calculated as mean ± SD (n=3). * $P < 0.05$; Student's *t* test.

(b-c) BT474 cells generated in (**a**) were subjected to clonogenic survival assays in RPMI1640 medium supplemented with 10% FBS for 14 days. Crystal violet was used to stain the formed colonies (**b**) and the colony numbers were calculated as mean ± SD (n=3), * $P < 0.05$; Student's *t* test (**c**).

(d-e) BT474 cells generated in (**a**) were subjected to 3D spheroid formation experiments in RPMI1640 medium supplemented with 10% FBS for 21 days. The representative pictures are shown from three independent experiments (**d**), Scale bar, 100 µm. Anchorage-independent cell growth was measured using CellTiter-Glo 3D Cell Viability Assay Kit at the indicated time points (**e**). Relative cell viability was calculated as mean ± SD (n=3). * $P < 0.05$; Student's *t* test (**e**).

(f-g) MDA-MB-231 cells generated in **Supplementary Figure 1m** were subjected to clonogenic survival assays in DMEM medium supplemented with 10% FBS for 14 days. Crystal violet was used to stain the formed colonies (**f**) and the relative colony numbers were calculated as mean ± SD (n=3), * $P < 0.05$; Student's *t* test (**g**).

(h-i) MDA-MB-231 cells generated in **Supplementary Figure 1m** were subjected to soft agar colony formation assays for 21 days. Formed colonies were stained with iodonitrotetrazolium chloride (INT) (**h**). The relative colony numbers were calculated as mean ± SD (n=3). * $P < 0.05$; Student's *t* test (**i**).

(j-k) Tumor pictures (**j**) and the growth curves (**k**) for the xenograft experiments with the BT474 cells generated in (**a**) were inoculated subcutaneously. In each flank of six nude mice, 3×10^6 cells were injected. The visible tumors were measured at the indicated days. Error bars represent ±SEM (n=6) (**k**).

(l-m) MDA-MB-231 cells generated in **Fig. 1k** were subjected to immunofluorescence staining using anti-centromere antibody (ACA), tubulin antibody and DAPI (DNA) as indicated (**l**). The percentage of M phase cells containing chromosome segregation defects was quantified (**m**, n = 30). Representative images are shown in (**l**). Scale bar, 10 µm.

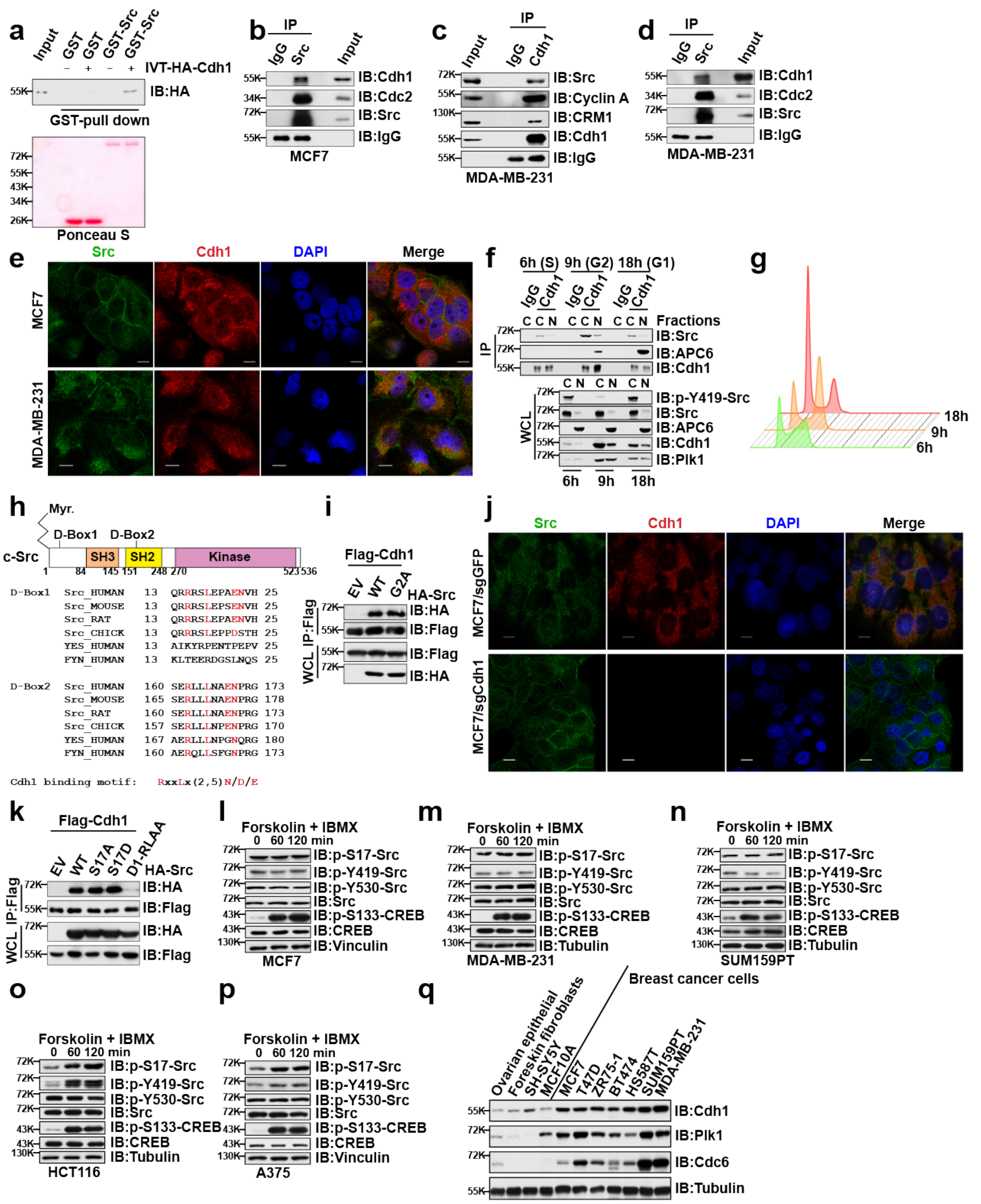

a
Input
GST
GST-Src
IVT-HA-Cdh1
IB:HA
GST-pull down
Ponceau S
b
IP
IgG
Src
Input
IB:Cdh1
IB:Cdc2
IB:Src
IB:IgG
MCF7
c
Cdh1
IB:Cyclin A
IB:CRM1
MDA-MB-231
d
e
DAPI
Merge
f
6h (S) 9h (G2) 18h (G1)
Fractions
IB:APC6
IB:p-Y419-Src
IB:Plk1
WCL
g
18h
9h
6h
h
Myr.
D-Box1 D-Box2
c-Src
SH3
SH2
Kinase
D-Box1 Src_HUMAN 13 QRRRSLEPAENVH 25
Src_MOUSE 13 QRRRSLEPSENVH 25
Src_RAT 13 QRRRSLEPAENVH 25
Src_CHICK 13 QRRRSLEPPDSTH 25
YES_HUMAN 13 AIKYRPENTPEPV 25
FYN_HUMAN 13 KLTEERDGSLNQS 25
D-Box2 Src_HUMAN 160 SERLLLNAENPRG 173
Src_MOUSE 165 SERLLLNAENPRG 178
Src_RAT 160 SERLLLNAENPRG 173
Src_CHICK 157 SERLLLNPENPRG 170
YES_HUMAN 167 AERLLLNPGNQRG 180
FYN_HUMAN 160 AERQLLSFGNPRG 173
Cdh1 binding motif: RxxLx(2,5)N/D/E
i
Flag-Cdh1
EV
WT
G2A
HA-Src
IB:Flag
IP:Flag
j
MCF7/sgGFP
MCF7/sgCdh1
k
S17A
S17D
D1-RLAA
l
Forskolin + IBMX
0 60 120 min
IB:p-S17-Src
IB:p-Y530-Src
IB:p-S133-CREB
IB:CREB
IB:Vinculin
m
IB:Tubulin
n
SUM159PT
o
HCT116
p
A375
q
Breast cancer cells
Ovarian epithelial
Foreskin fibroblasts
SH-SY5Y
MCF10A
T47D
ZR75-1
BT474
HS587T
IB:Cdc6

Supplementary Figure 3 Cdh1 binds Src mainly in the cytoplasm.

(a) *In vitro* transcribed and translated (IVT) HA-Cdh1 bound to purified recombinant GST-Src, but not the GST recombinant protein.

(b-d) Immunoblot (IB) analysis of WCL and anti-Src (**b, d**) or anti-Cdh1 (**c**) immunoprecipitates (IP) derived from MCF7 (**b**) and MDA-MB-231 (**c-d**) cells.

(e) Immunofluorescent staining of MDA-MB-231 and MCF7 cells using Cdh1 and Src antibodies. Scale bar, 50 µm.

(f-g) MDA-MB-231 cells were synchronized by double thymidine block. Cells were then released to the cell cycle for the indicated period of time (**g**) before being subjected to cytoplasm/nucleus fraction and anti-Cdh1 IP (**f**).

(h) A schematic illustration of sequence alignments of the putative D-boxes–containing region between Src proteins from various species.

(i) IB analysis of WCL and IP derived from 293T cells transfected with Flag-Cdh1 and the indicated HA-Src constructs.

(j) Immunofluorescent staining of control and sg*Cdh1*-MCF7 cells using Cdh1 and Src antibodies. Scale bar, 50 µm.

(k) IB analysis of WCL and IP derived from 293T cells transfected with Flag-Cdh1 and the indicated HA-Src constructs.

(l-p) MCF7 (**l**), MDA-MB-231 (**m**), SUM159PT (**n**), HCT116 (**o**) and A375 (**p**) cells were treated with PKA agonists Forskolin (10 µM) and IBMX (100 µM) for the indicated period of time before harvest.

(q) IB analysis of WCL derived from a panel of non-transformed cells and breast cancer cell lines.

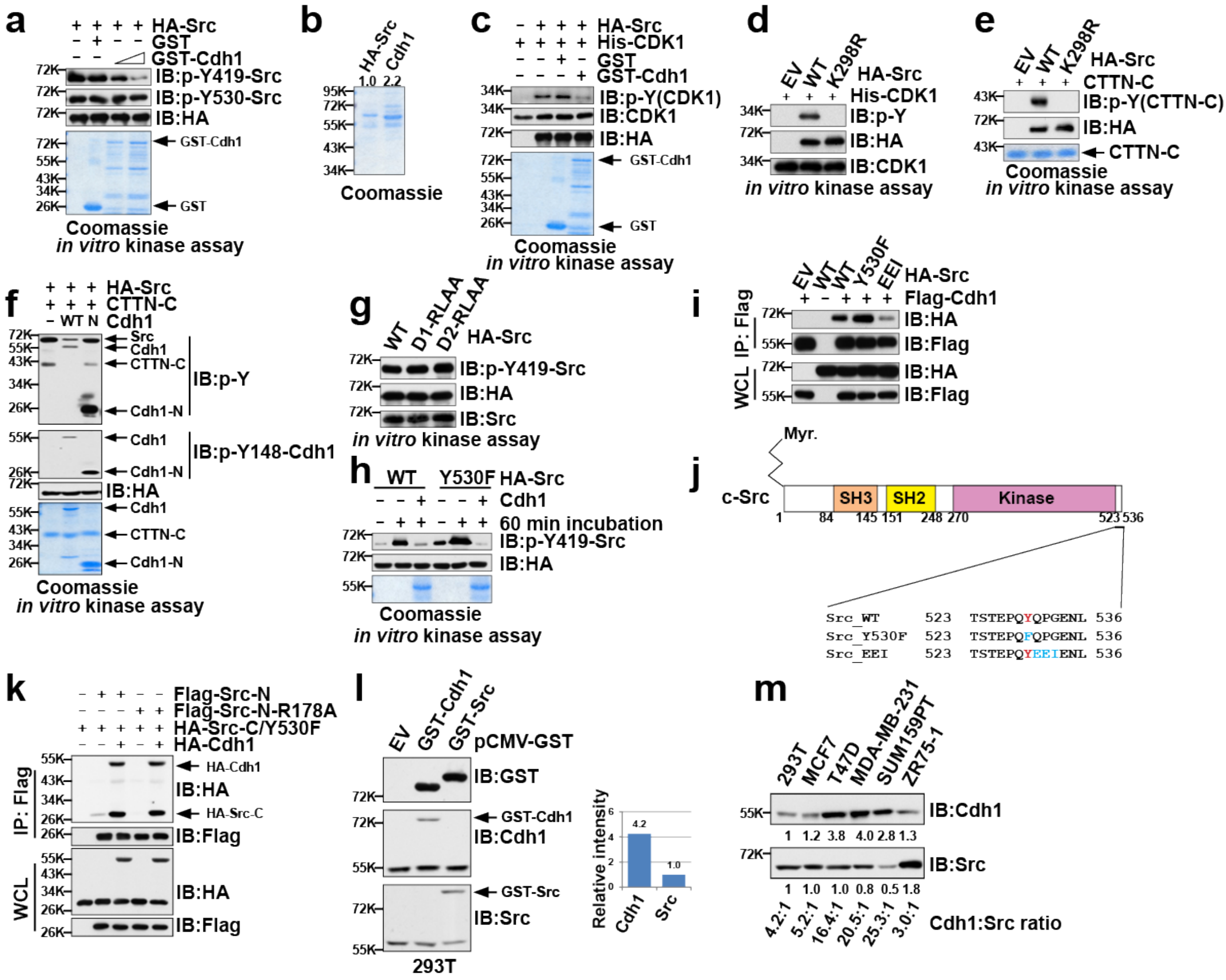

Supplementary Figure 4 Cdh1 suppresses Src kinase activity.

(a) Immuno-purified HA-Src from 293T cells were incubated with bacterially purified GST-Cdh1 as indicated. The reaction was performed at 30°C for 60 minutes followed by SDS-PAGE and Immunoblot (IB) analyses.

(b) Quantification of the relative protein amount of immuno-purified HA-Src and recombinant and PreScission-cleaved Cdh1 proteins that were used in the *in vitro* kinase assays. HA-Src proteins were loaded with 10 × higher amount than those used in the *in vitro* kinase assays.

(c) Recombinant GST-Cdh1 inhibited the kinase activity of Src to promote CDK1 phosphorylation *in vitro.*

(d-e) *In vitro* kinase assays showing that WT-Src, but not the K298R inactive mutant, promoted the phosphorylation of CDK1 (**d**) and CTTN-*C* (**e**). Immuno-purified WT- and K298R-HA-Src from 293T cells were incubated with bacterially purified His-CDK1 or CTTN-*C* as indicated. The reaction was performed at 30°C for 60 minutes and followed by SDS-PAGE and IB analyses.

(f) Recombinant full-length Cdh1, but not *N*-terminal Cdh1 inhibited the kinase activity of Src *in vitro.*

(g) *In vitro* kinase assays showing that immuno-purified WT, D-box1-mutated and D-box2-mutated HA-Src displayed similar kinase activity *in vitro* as determined by Src p-Y419 autophosphorylation.

(h) Recombinant Cdh1 suppressed the kinase activities of both WT- and Y530F-Src *in vitro*.

(i) IB analysis of WCL and IP derived from 293T cells transfected with Flag-Cdh1 and the indicated HA-Src constructs.

(j) A schematic illustration of mutations generated on Src which mimics open conformation of Src (Y530F) and closed conformation of Src (EEI).

(k) IB analysis of WCL and IP derived from 293T cells transfected with HA-Cdh1 and the indicated Src constructs.

(l) Quantification of the relative protein amount of Cdh1 and Src proteins in 293T cells. pCMV-GST-Cdh1 and pCMV-GST-Src constructs were transfected into 293T cells using doses with matched expression level. GST-Cdh1 and GST-Src protein levels were then compared to endogenous Cdh1 and Src levels using Cdh1 and Src antibodies,

respectively. The ratio between endogenous Cdh1 and endogenous Src were then calculated using GST-Cdh1 and GST-Src as references. ImageJ was used for densitometry determination.

(m) Comparison of Cdh1 and Src expression levels in a panel of cell lines.

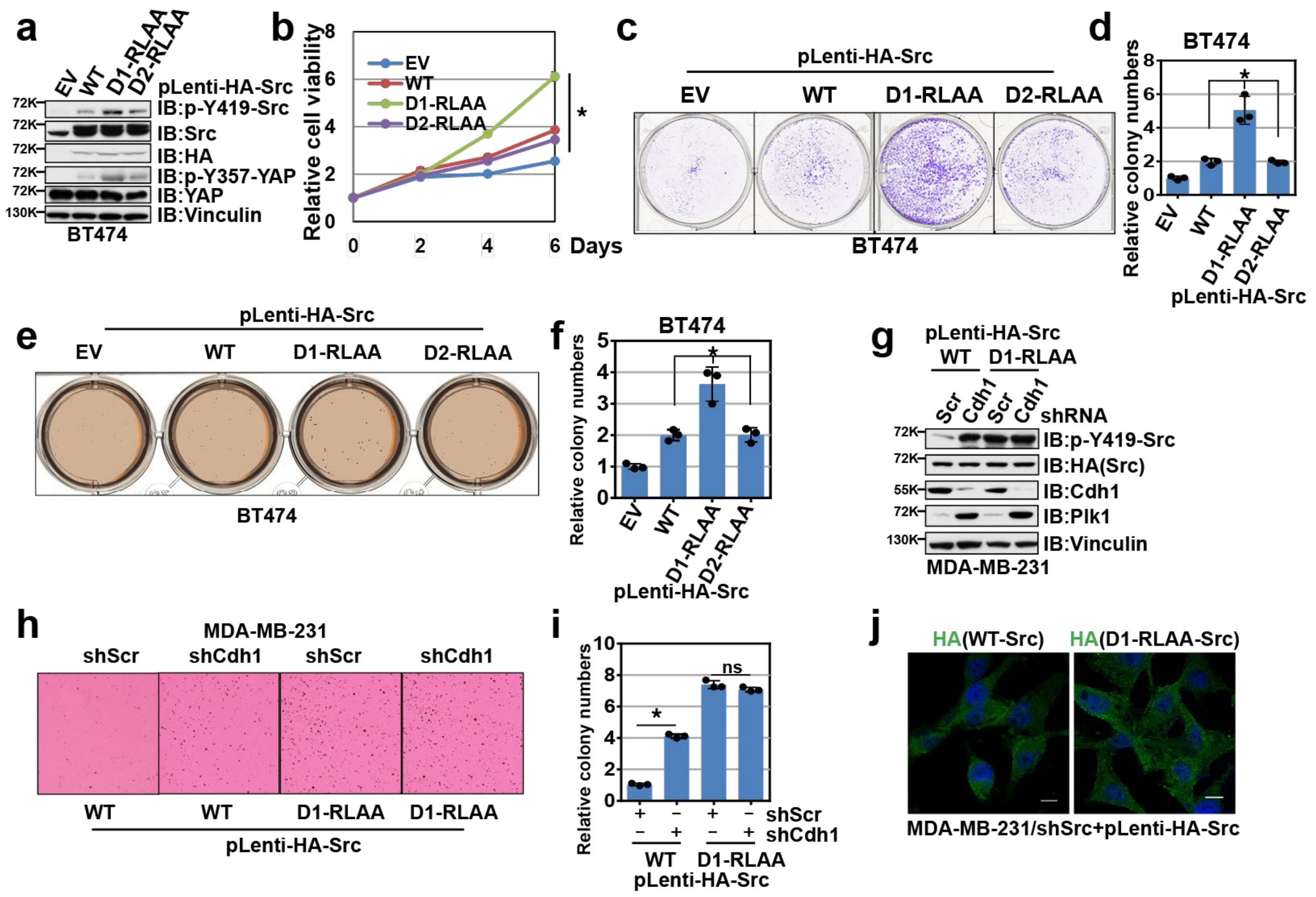

Supplementary Figure 5 Cdh1-binding deficient Src mutant promotes breast cancer cell growth.

(a) Immunoblot (IB) analysis of whole-cell lysates (WCL) derived from BT474 cells stably expressing empty vector (EV), WT-Src, D-box-1-mutated or D-box-2-mutated Src.

(b) BT474 cells generated in (**a**) were subjected to cell proliferation assays in RPMI1640 medium supplemented with 10% FBS for 6 days. Cell viability was determined at the indicated time points. The relative viability was calculated as mean ± SD (n=3). * $P < 0.05$; Student's *t* test.

(c-d) BT474 cells generated in (**a**) were seeded for clonogenic survival assays (1,000 cells per well). 14 days after plating, crystal violet was used to stain the formed colonies (**c**) and the relative colony numbers were counted as mean ± SD (n=3), * $P < 0.05$; Student's *t* test (**d**).

(e-f) BT474 cells generated in (**a**) were subjected to soft agar colony formation assays for 21 days. Formed colonies were stained with iodonitrotetrazolium chloride (INT) (**e**). The relative colony numbers were calculated as mean ± SD (n=3). * $P < 0.05$; Student's *t* test (**f**).

(g) MDA-MB-231 cells stably expressing WT- or D1-RLAA-Src were infected with shScr or shCdh1 lentiviral constructs as indicated followed by 1 µg/mL puromycin selection for 72 h before harvest.

(h-i) MDA-MB-231 cells generated in (**g**) were subjected to soft agar colony formation assays for 21 days. Formed colonies were stained with iodonitrotetrazolium chloride (INT) **(h).** The relative colony numbers were calculated as mean ± SD (n=3). * $P < 0.05$; Student's *t* test (**i**).

(j) MDA-MB-231 cells generated in **Fig. 5a** were subjected to immunofluorescent staining using anti-HA antibody. Scale bar, 50 µm.

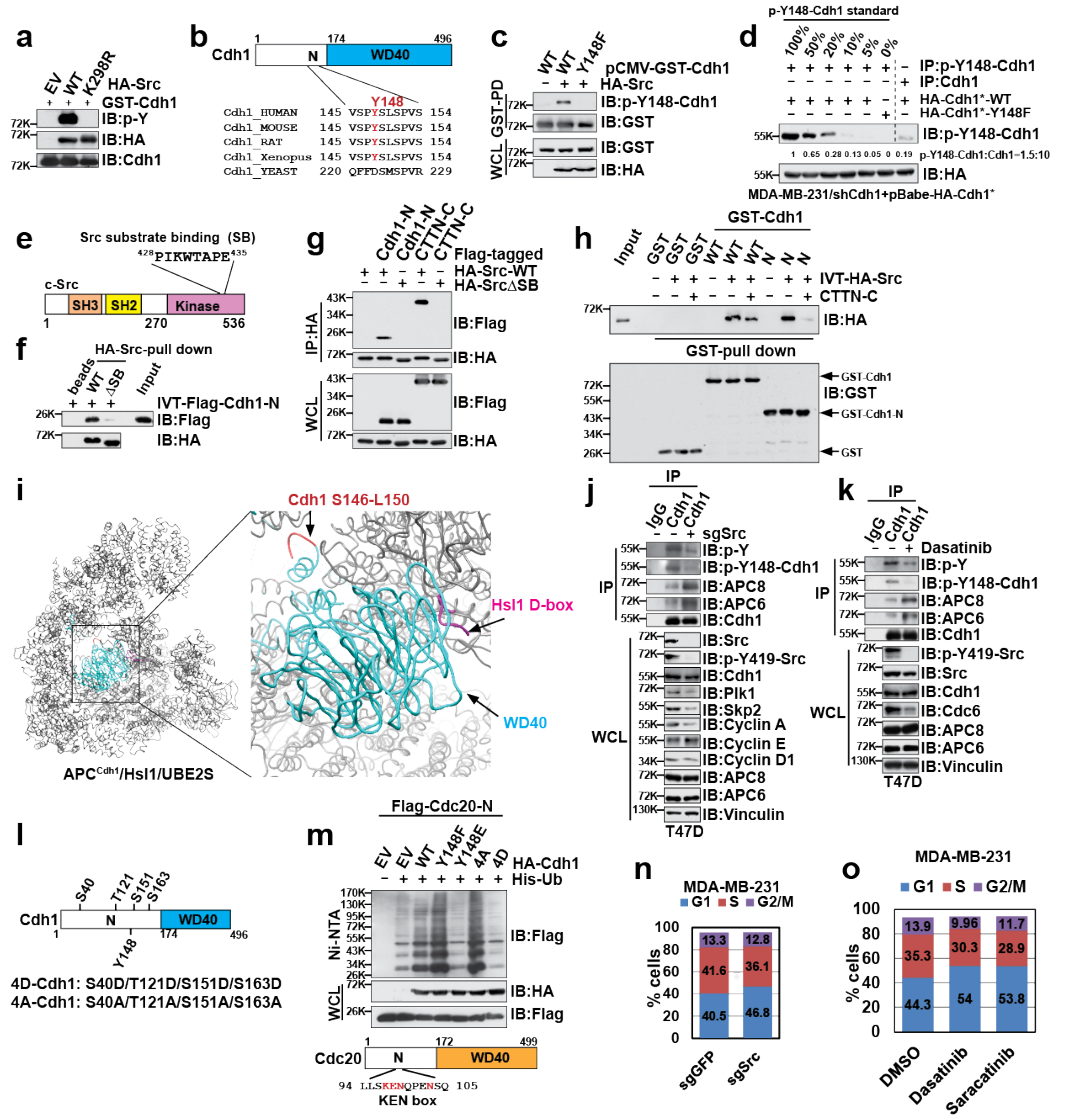

Supplementary Figure 6 Src phosphorylates Cdh1 at Y148 to inhibit APC^Cdh1 E3 ligase activity.

(a) *In vitro* kinase assays showing that bacterially purified GST-Cdh1 *N*-terminus was phosphorylated by immuno-purified WT, but not the enzymatic deficient K298R-HA-Src using phospho-tyrosine (p-Y) antibody.

(b) Sequence alignments of the putative Y148 site-containing region among Cdh1 proteins from various species.

(c) A specific anti-p-Y148-Cdh1 was generated and validated using WT- and Y148-Cdh1 constructs described in **Fig. 6g**.

(d) The percentage of Cdh1 Y148 phosphorylation in MDA-MB-231 cells was estimated using the p-Y148-Cdh1 antibody. Anti-p-Y148-Cdh1 immunoprecipitations (IP) using cell lysates derived from shCdh1-MDA-MB-231 cells stably expressing HA-Cdh1-WT or the Y148F mutant were performed to generate the 100% p-Y148-Cdh1 and the 0% p-Y148-Cdh1 standards, respectively. These two standard IP samples were normalized to Cdh1 total protein amount using anti-HA IB followed by mixing at the indicated ratio as shown in lanes 1-6. An anti-Cdh1 IP was performed using shCdh1-MDA-MB-231 cells stably expressing HA-Cdh1-WT and normalized (lane 7). Anti p-Y148-Cdh1 IB was

carried out using both the standards and the sample IP, the IB:p-Y148-Cdh1 band intensities were determined using ImageJ and Cdh1 Y148 phosphorylation percentage in MDA-MB-231 cells was estimated by comparing to the standards. *Cdh1 cDNA used in this experiment has been mutated to escape sh*Cdh1*-mediated gene silencing.

(e) A schematic illustration of the previously identified Src substrate binding motif (SB).

(f) *In vitro* HA-pull down experiment showing that Src substrate binding motif (SB)-deleted Src mutant failed to bind Cdh1 *N*-terminal domain.

(g) IB analysis of WCL and anti-HA immunoprecipitates (IP) derived from 293T cells transfected with either *N*-terminal Cdh1 or *C*-terminal CTTN and the indicated HA-Src constructs.

(h) GST pull-down analysis showing that the *C*-terminal domain of CTTN, a well-characterized Src substrate, compete with Cdh1 to bind Src *in vitro*.

(i) Structural illustrations of Cdh1 WD40 domain and the *N*-terminal Y148 residue in a reported APC^{Cdh1} complex Cryo-EM structure [1] (PDB ID: 1UWH). The illustration was generated using UCSF Chimera.

(j) Deletion of *Src* attenuated Cdh1 tyrosine phosphorylation and increased the binding of Cdh1 with the APC core complex. IB analysis of WCL and anti-Cdh1 IP derived from control or sg*Src*-infected T47D cells. Cells were pretreated with 4 $ng{\cdot}ml^{-1}$ PDGF for 30 min before harvest.

(k) IB analysis of WCL and anti-Cdh1 IP derived from DMSO or dasatinib-treated T47D cells. Cells were pretreated with 4 $ng{\cdot}ml^{-1}$ PDGF for 30 min before harvest.

(l) A schematic illustration of the previously identified four serine/threonine phosphorylation site and the Y148 residue in the Cdh1 *N*-terminal domain.

(m) IB analysis of WCL and Ni-NTA affinity precipitates derived from 293T cells transfected with His-UB, Flag-Cdc20-*N*-terminal domain and the indicated HA-Cdh1 constructs. Cells were pretreated with 10 μM MG132 for 12 hours before harvest. A schematic illustration of the Cdc20 KEN box was shown underneath the result.

(n) Cell cycle profiling of sgGFP- and sg*Src*-MDA-MB-231 cells.

(o) Cell cycle profiling of MDA-MB-231 cells treated with DMSO, 100 nM dasatinib or 100 nM saracatinib for 24h prior to FACS analysis.

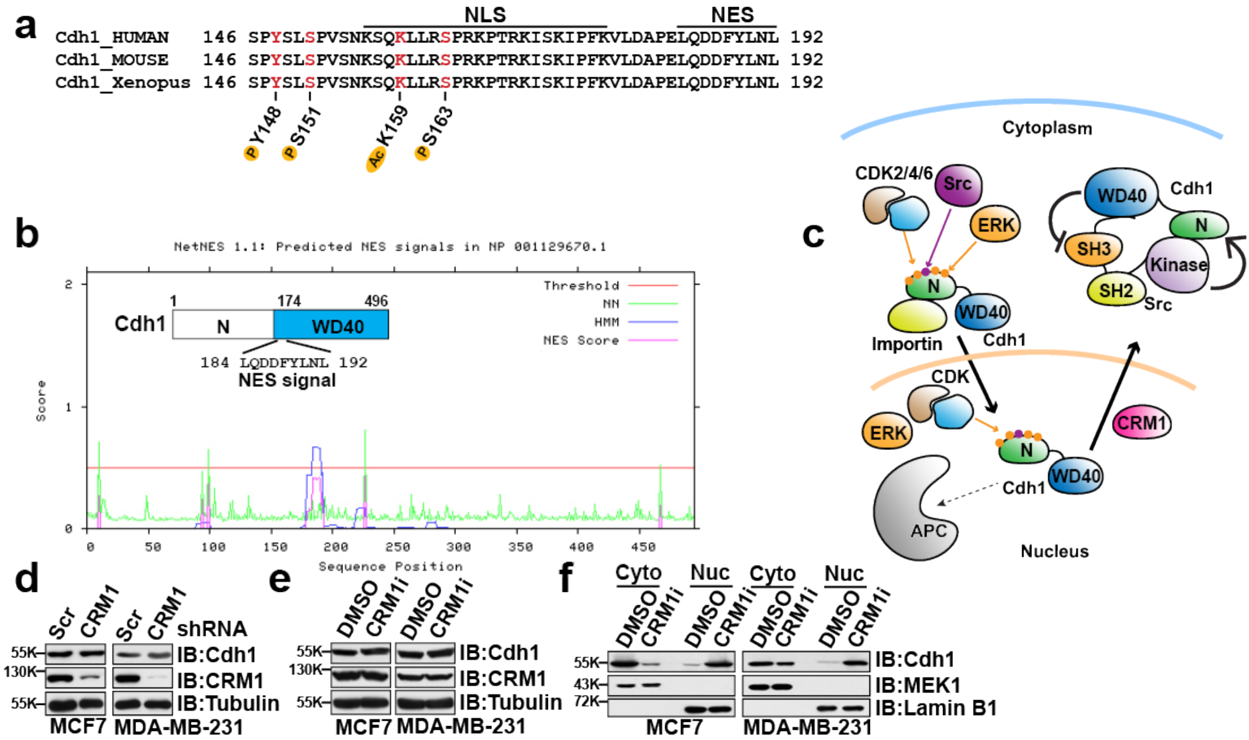

Supplementary Figure 7 Identification of a nuclear export signal in the *N*-terminal domain of Cdh1.

(a) Sequence alignments of part of the *N*-terminal Cdh1 sequence encompassing the NLS and NES motifs from various species.

(b) Prediction of the Cdh1 NES sequence using the NetNES 1.1 Server.

(c) A schematic illustration of the proposed model that *N*-terminal phosphorylation of Cdh1 facilitates its binding with CRM1 and subsequent cytoplasmic translocation.

(d) MCF7 and MDA-MB-231 cells were infected with shScr or sh*CRM1* lentiviral shRNA constructs. The infected cells were selected with 1 μg·ml^{-1} puromycin for 72 h before harvesting for IB analysis.

(e) MCF7 and MDA-MB-231 cells were treated with 5 nM Leptomycin B (CRM1i) for 24h before harvest for IB analysis.

(f) MCF7 and MDA-MB-231 cells were treated with 5 nM Leptomycin B (CRM1i) for 24h before harvest for cytoplasm/nucleus fractionation and IB analysis.

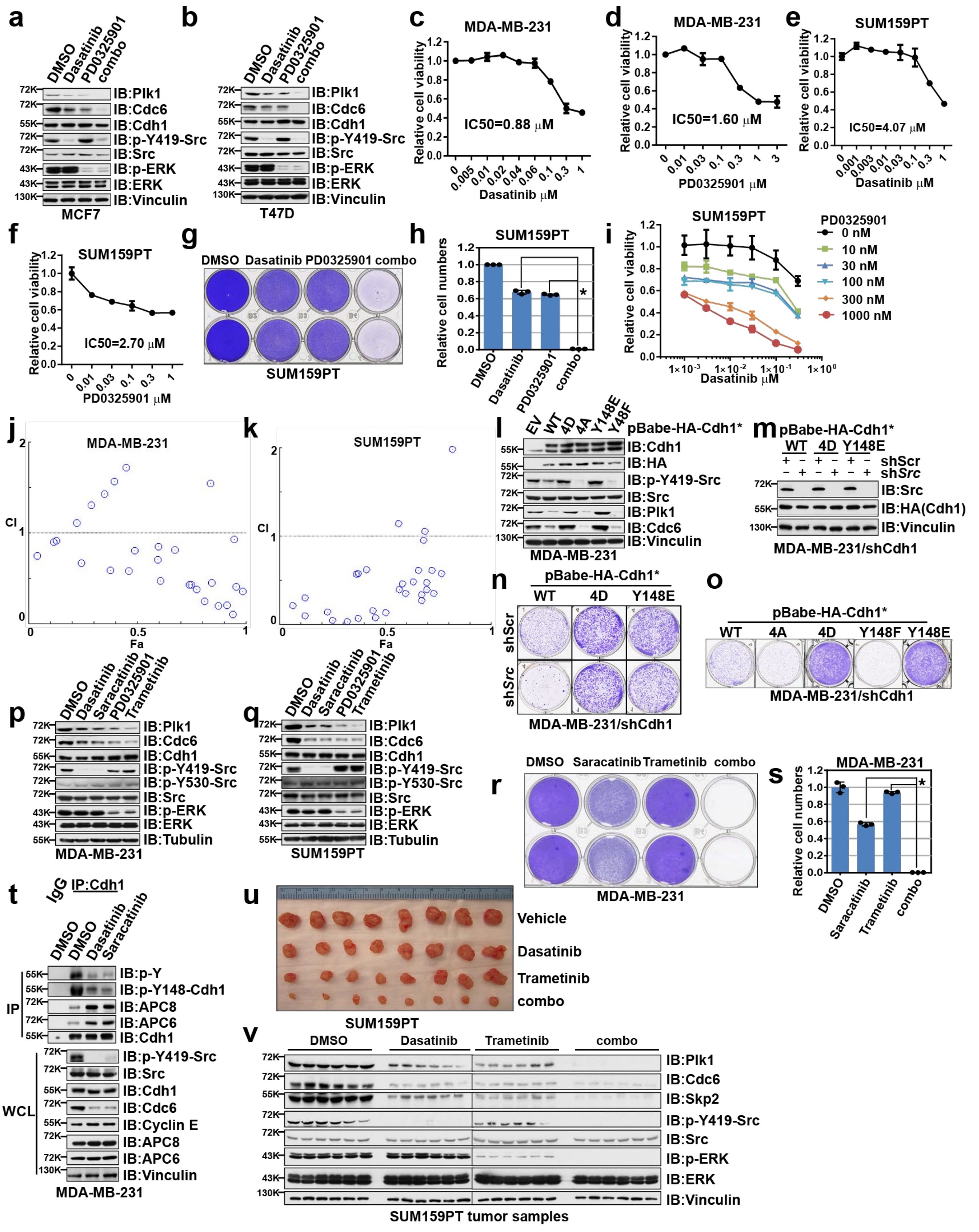
a
DMSO
Dasatinib
PD0325901
combo
IB:Plk1
IB:Cdc6
IB:Cdh1
IB:p-Y419-Src
IB:Src
IB:p-ERK
IB:ERK
IB:Vinculin
MCF7
b
T47D
c
MDA-MB-231
IC50=0.88 μM
Relative cell viability
Dasatinib μM
d
MDA-MB-231
IC50=1.60 μM
PD0325901 μM
e
SUM159PT
IC50=4.07 μM
Dasatinib μM
f
SUM159PT
IC50=2.70 μM
PD0325901 μM
g
DMSO Dasatinib PD0325901 combo
SUM159PT
h
SUM159PT
Relative cell numbers
i
SUM159PT
PD0325901
0 nM
10 nM
30 nM
100 nM
300 nM
1000 nM
Dasatinib μM
j
MDA-MB-231
CI
Fa
k
SUM159PT
l
EV
WT
4D
4A
Y148E
Y48F
pBabe-HA-Cdh1*
IB:HA
MDA-MB-231
m
pBabe-HA-Cdh1*
shScr
shSrc
IB:HA(Cdh1)
MDA-MB-231/shCdh1
n
pBabe-HA-Cdh1*
MDA-MB-231/shCdh1
o
pBabe-HA-Cdh1*
Y148F
MDA-MB-231/shCdh1
p
Saracatinib
Trametinib
IB:p-Y530-Src
IB:Tubulin
MDA-MB-231
q
SUM159PT
r
DMSO Saracatinib Trametinib combo
MDA-MB-231
s
MDA-MB-231
t
IgG IP:Cdh1
IB:p-Y
IB:p-Y148-Cdh1
IB:APC8
IB:APC6
IP
WCL
IB:Cyclin E
MDA-MB-231
u
Vehicle
Dasatinib
Trametinib
combo
SUM159PT
v
DMSO
Dasatinib
Trametinib
combo
IB:Skp2
SUM159PT tumor samples

Supplementary Figure 8 Src and MEK inhibitors synergistically suppress breast cancer cell survival.

(a-b) Protein levels of APC^{Cdh1} ubiquitin substrates reduced upon Src or MEK inhibition in MCF7 (**a**) and T47D (**b**) cells. Immunoblot (IB) analysis of MCF7 (**a**) and T47D (**b**) cells treated with either 100 nM Src inhibitor dasatinib, 100 nM MEK inhibitor PD0325901, a combination of these two inhibitors (combo), or DMSO as a negative control for 24 h before harvest.

(c-d) IC50s of dasatinib and PD0325901 were determined in MDA-MB-231 cells. MDA-MB-231 cells were treated with different doses of dasatinib (**c**) and PD0325901 (**d**) as indicated. Relative cell viability was determined after 72 h treatment. IC50 values were calculated using Graphpad Prism.

(e-f) IC50s of dasatinib and PD0325901 were determined in SUM159PT cells. SUM159PT cells were treated with different doses of dasatinib (**e**) and PD0325901 (**f**) as indicated. Relative cell viability was determined after 72 h treatment. IC50 values were calculated using Graphpad Prism.

(g-h) Colony formation assays showing the synergism of dasatinib and PD0325901 in SUM159PT cells. Cells were treated with 300 nM dasatinib, 300 nM PD0325901, or the combination of dasatinib with PD0325901 for 3 days before being fixed and stained with crystal violet (**g**). Relative colony numbers were calculated as mean ± SD (n=3). * $P < 0.05$; Student's *t* test (**h**).

(i) Dose-response curves of SUM159PT cells treated with dasatinib, PD0325901 or the combination. Relative cell viability was determined after 72 h treatment. Combination indexes (CI) were calculated using the CompuSyn software as shown in (**k**).

(j-k) Plot of combination index values of MDA-MB-231 (**j**) and SUM159PT (**k**) cells treated with dasatinib, PD0325901 or the combination of dasatinib and PD0325901. CI values were calculated using the CompuSyn software with the Chou-Talalay equation.

(l) IB analysis of WCL derived from MDA-MB-231 stably expressing the indicated retroviral HA-Cdh1 constructs.

(m) MDA-MB-231 cells generated in (**l**) were further depleted with *Cdh1* and *Src* as indicated for IB analysis.

(n) MDA-MB-231 cells generated in (**m**) were seeded for clonogenic survival assays (1,000 cells per well). 10 days after plating, crystal violet was used to stain the formed colonies.

(o) MDA-MB-231 cells generated in (**l**) were further depleted with *Cdh1* before seeding for clonogenic survival assays (1,000 cells per well). 10 days after plating, crystal violet was used to stain the formed colonies.

(p-q) Protein levels of APC^{Cdh1} ubiquitin substrates reduced upon Src or MEK inhibition in MDA-MB-231 (**p**) and SUM159PT (**q**) cells. IB analysis of MDA-MB-231 (**p**) and SUM159PT (**q**) cells treated with either 100 nM Src inhibitor dasatinib, 100 nM Src inhibitor saracatinib, 100 nM MEK inhibitor PD0325901, 100 nM MEK inhibitor trametinib, or DMSO as a negative control for 24 h before harvest.

(r-s) Colony formation assays showing the synergism of saracatinib and trametinib in MDA-MB-231 cells. Cells were treated with 300 nM saracatinib, 300 nM trametinib, or the combination of saracatinib with trametinib for 3 days before being fixed and stained with crystal violet (**r**). Relative colony numbers are calculated as mean ± SD (n=3). * $P < 0.05$; Student's *t* test (**s**).

(t) *N*-terminal phosphorylation and protein levels of APC^{Cdh1} ubiquitin substrates were reduced upon Src inhibition in MDA-MB-231 cells. IB analysis of MDA-MB-231 cells treated with either 100 nM Src inhibitor dasatinib or 100 nM Src inhibitor saracatinib, or DMSO as a negative control for 24 h before harvest.

(u) Tumor pictures at the end point of the SUM159PT xenograft tumors with the vehicle, dasatinib, trametinib or the combinational treatment as described in **Fig. 8k**.

(v) IB analysis of WCL derived from the tumor samples of the SUM159PT xenograft as described in **Fig. 8k.**

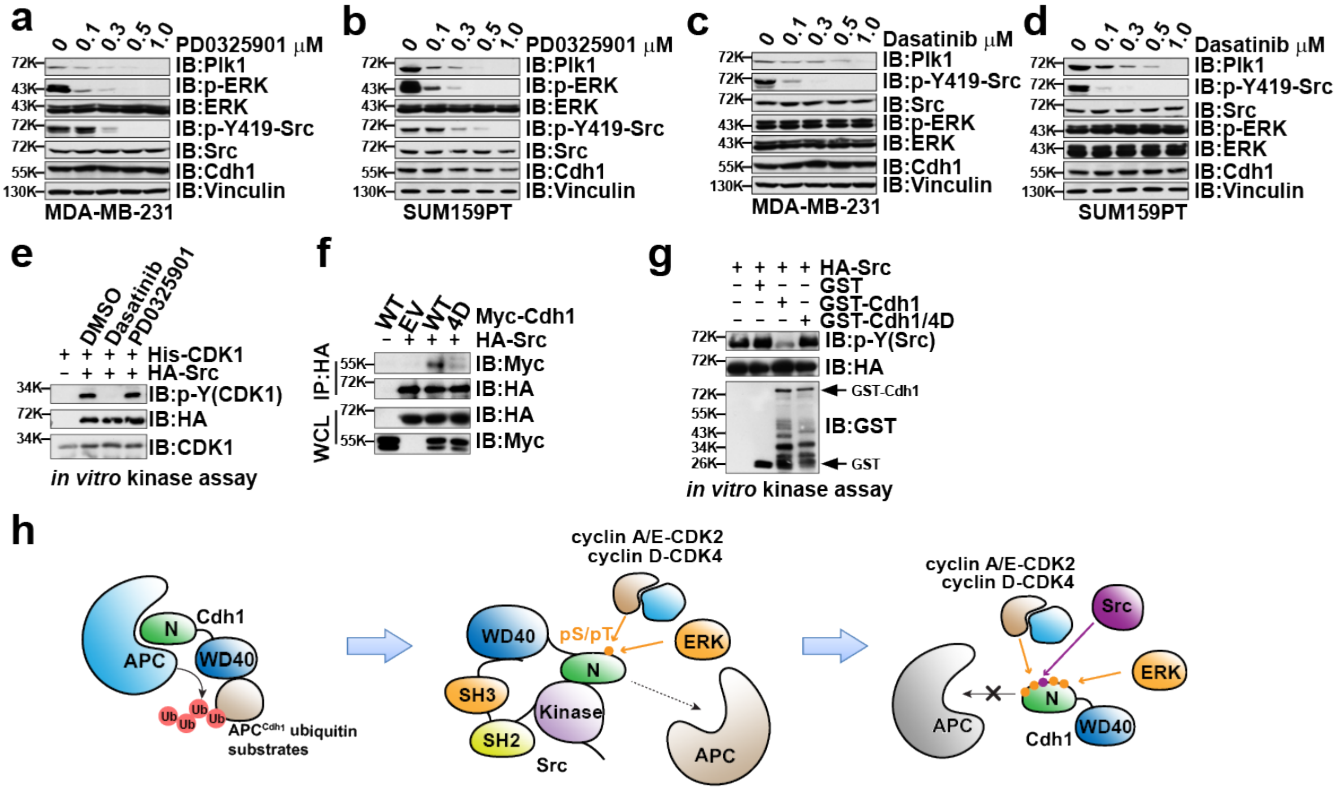

Supplementary Figure 9 Serine/threonine phosphorylated Cdh1 exhibited compromised function to suppress Src activity.

(a-b) Immunoblot (IB) analysis of MDA-MB-231 (**a**) and SUM159PT (**b**) cells treated with the indicated concentrations of MEK inhibitor PD0325901 for 24 h before harvest.

(c-d) IB analysis of MDA-MB-231 (**c**) and SUM159PT (**d**) cells treated with the indicated concentrations of Src inhibitor dasatinib for 24 h before harvest.

(e) *In vitro* kinase assays showing that only dasatinib, but not PD0325901, inhibits the phosphorylation of CDK1 by immuno-purified Src. Immuno-purified HA-Src from 293T cells was incubated with bacterially purified His-CDK1 as indicated together with 2.4 nM dasatinib or 10 nM PD0325901. The reaction was performed at 30 °C for 60 minutes and followed by SDS-PAGE and IB analysis.

(f) WT-Myc-Cdh1, but not 4D-Myc-Cdh1 binds to Src. IB analysis of WCL and IP derived from 293T cells transfected with HA-Src and the indicated Myc-Cdh1 constructs.

(g) *In vitro* kinase assays showing that 4D-Cdh1 failed to inhibit Src kinase activity.

(h) Schematic illustrations of the proposed mechanisms of a stepwise inactivation of APC^{Cdh1} E3 ligase activity via a cascade of phosphorylation events occurring at the *N*-terminal domain of Cdh1.

Supplementary Figure 10 Uncropped Original Scans

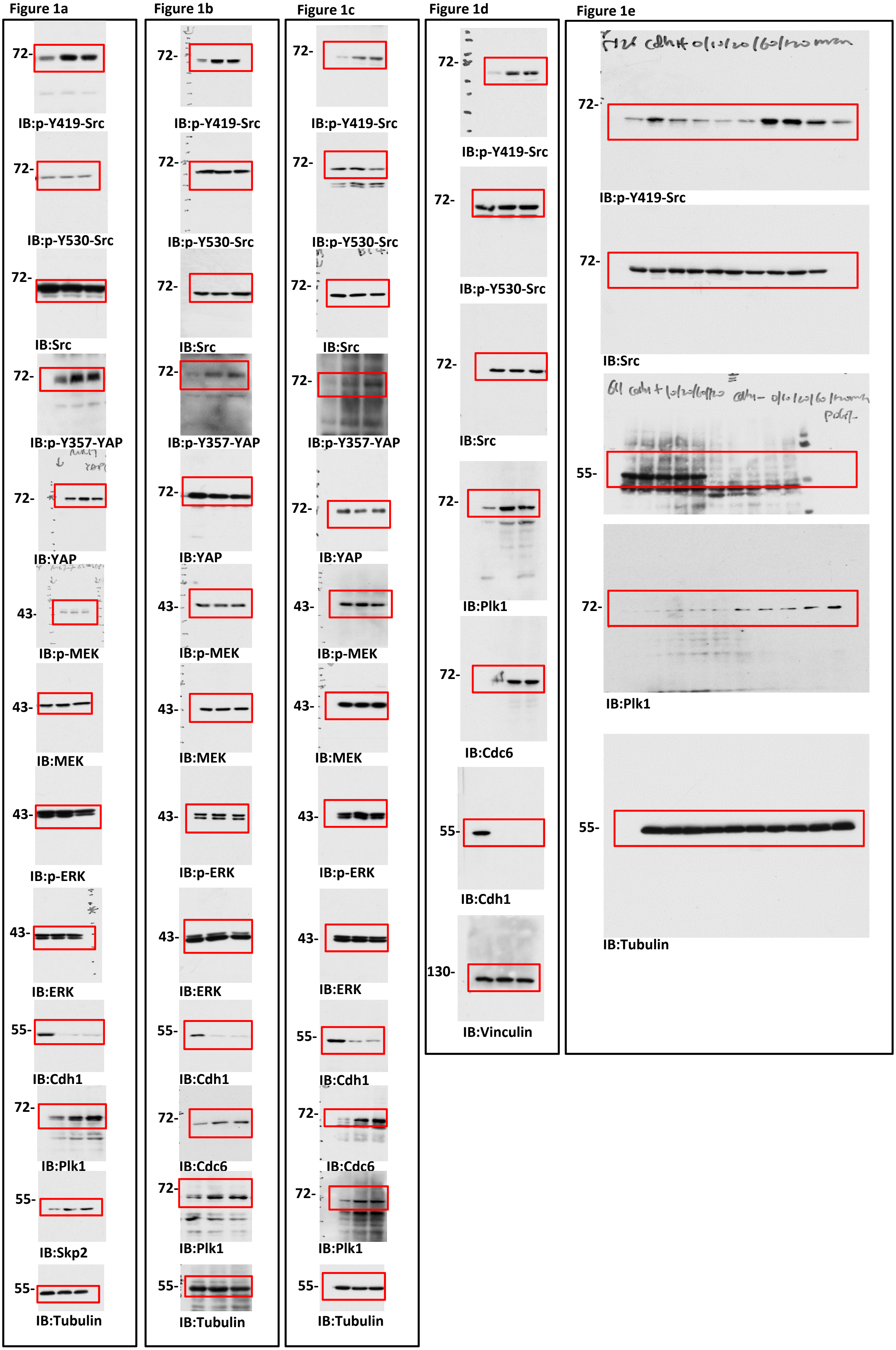

Uncropped immunoblots from Figure 1

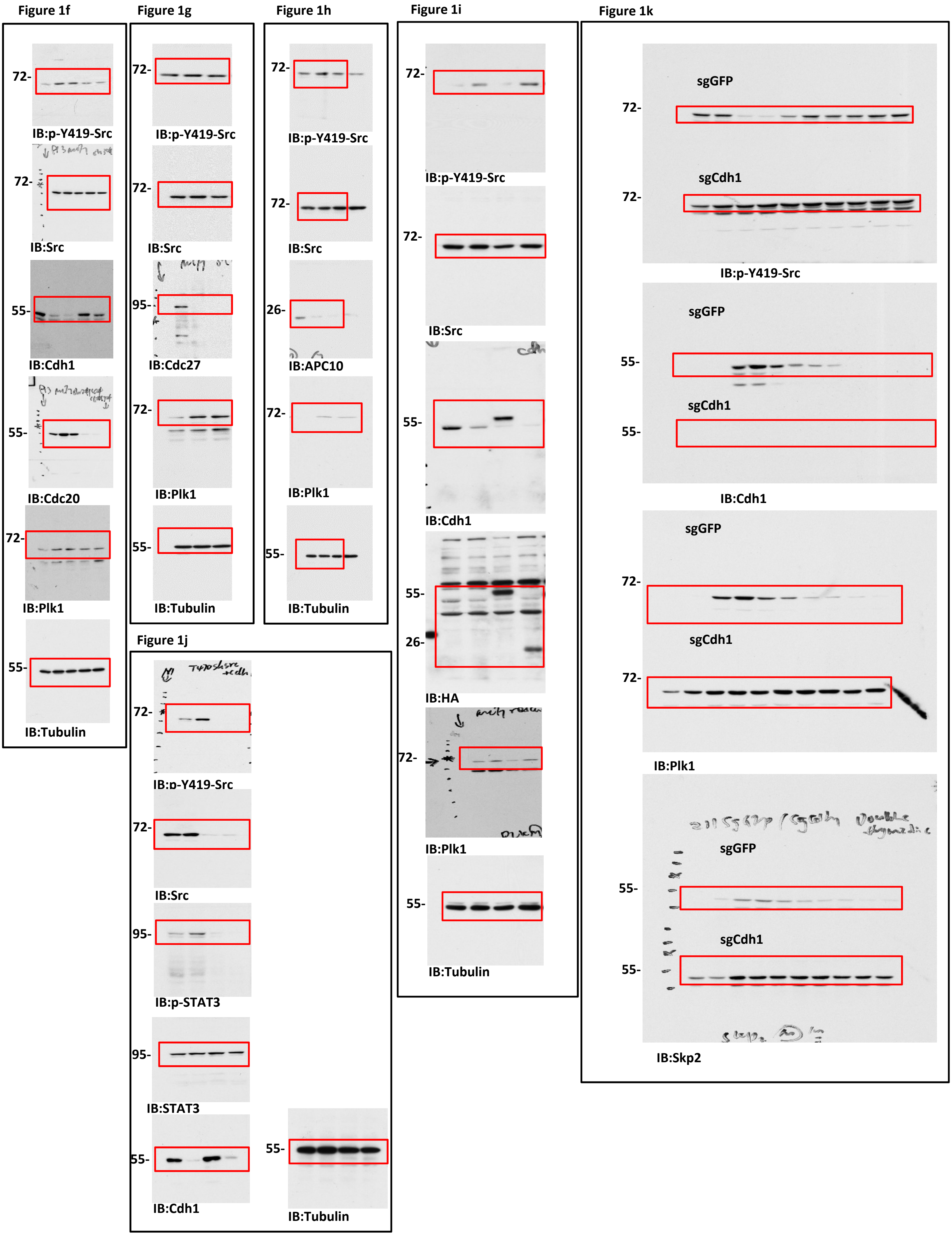

Uncropped immunoblots from Figure 1

Figure 1k

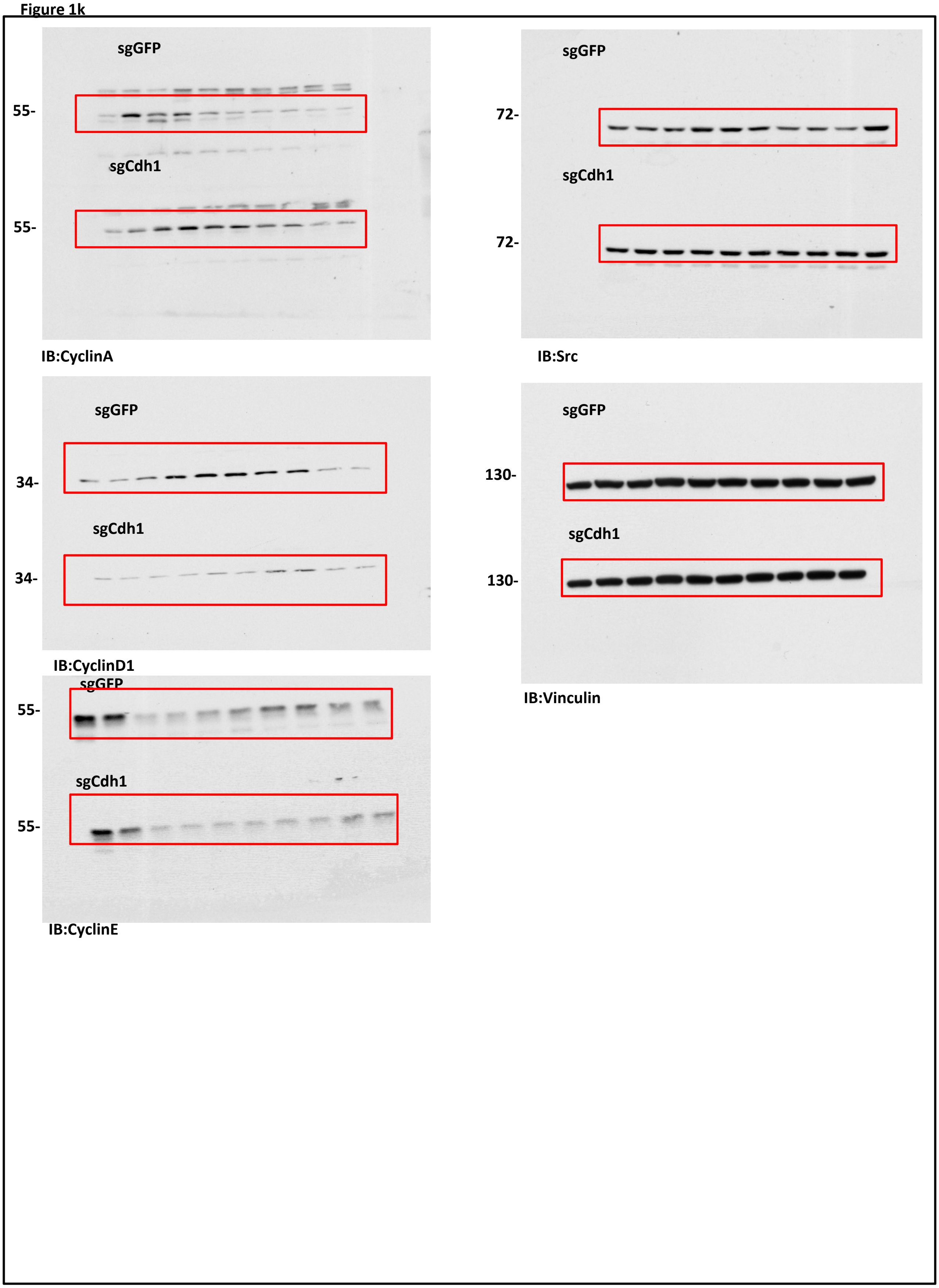

Uncropped immunoblots from Figure 1

Figure 2I

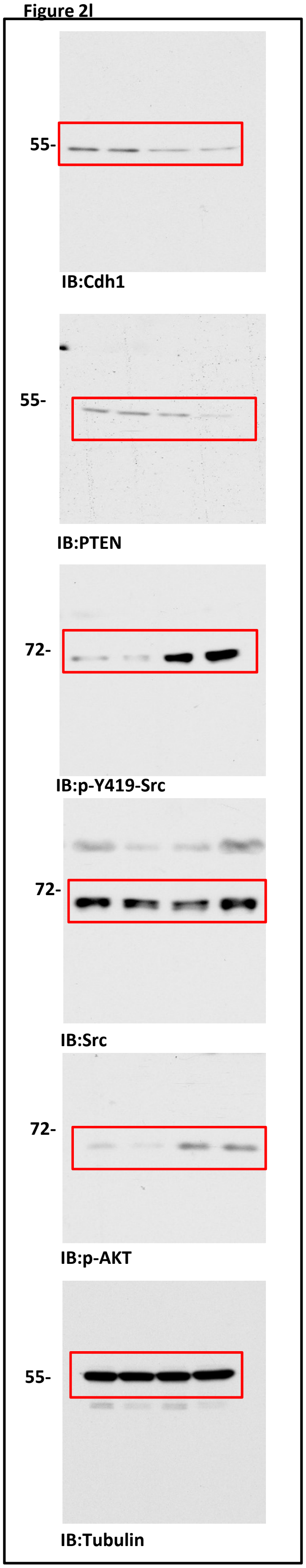

Uncropped immunoblots from Figure 2

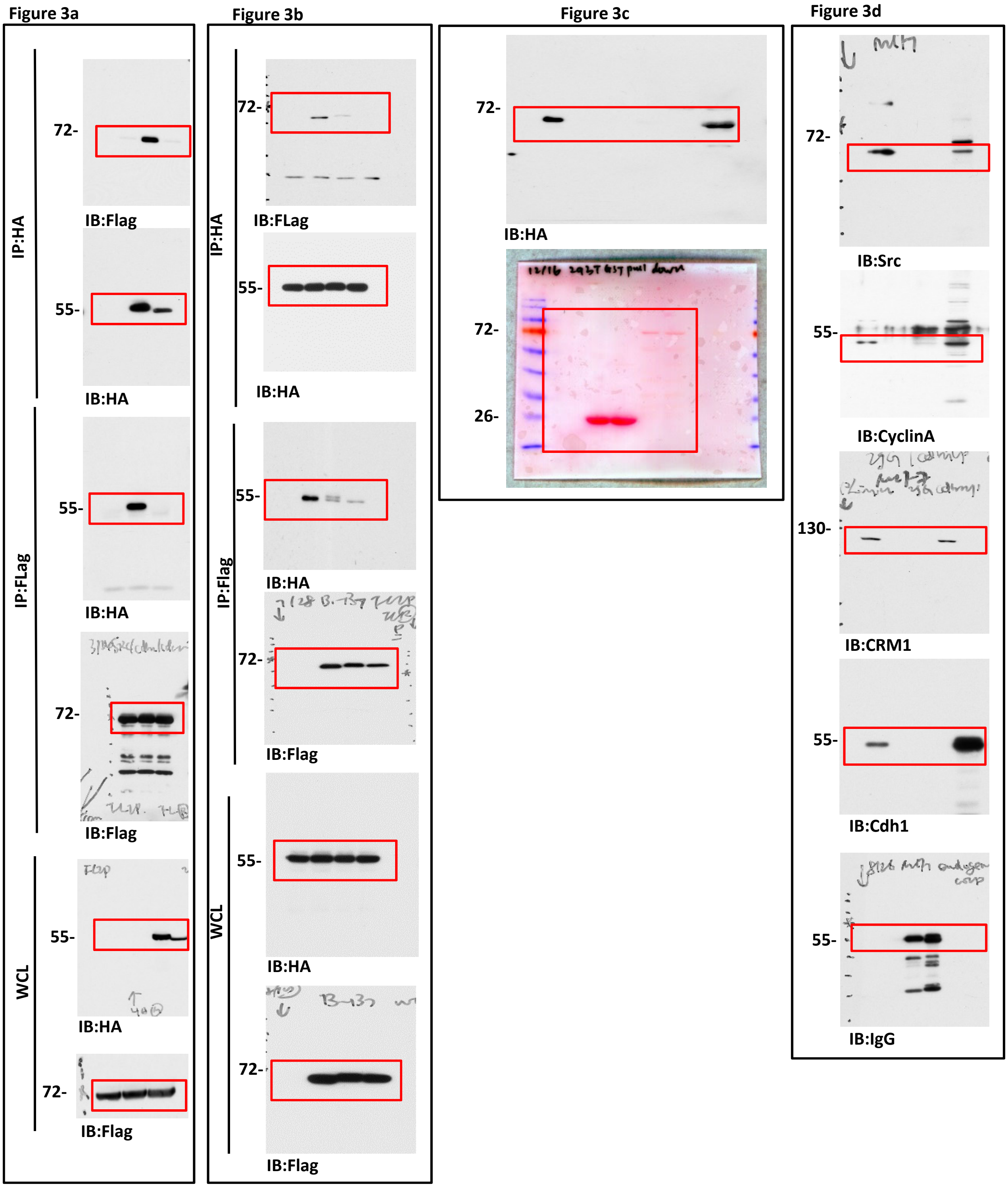

Uncropped immunoblots from Figure 3

Figure 3f

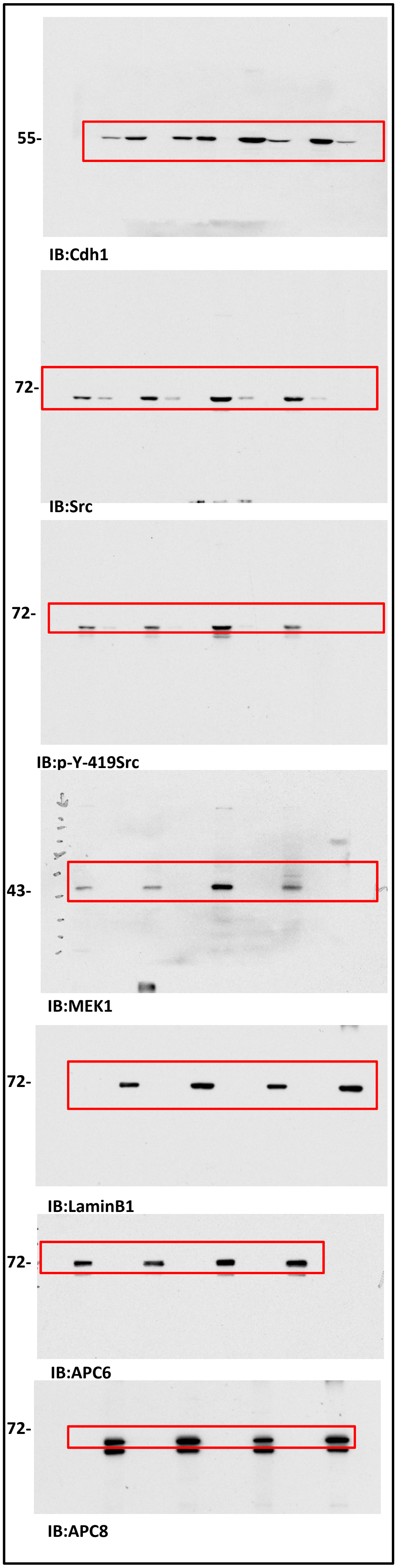

Figure 3h

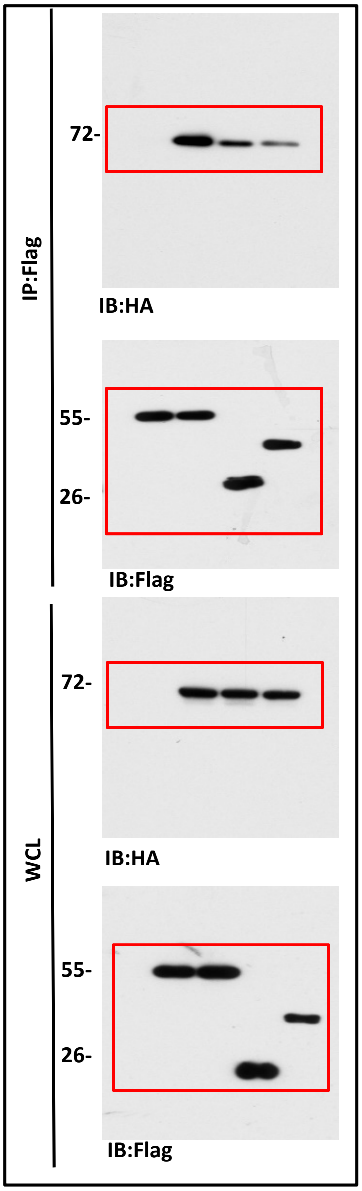

Figure 3i

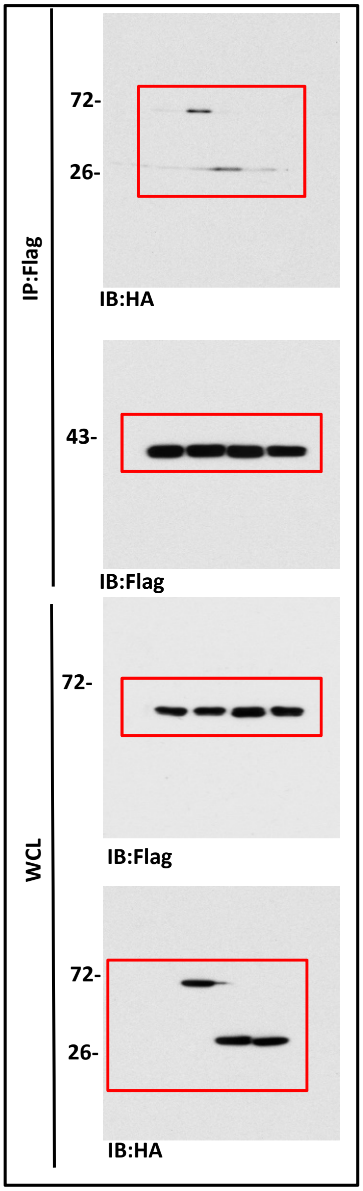

Uncropped immunoblots from Figure 3

Figure 3j

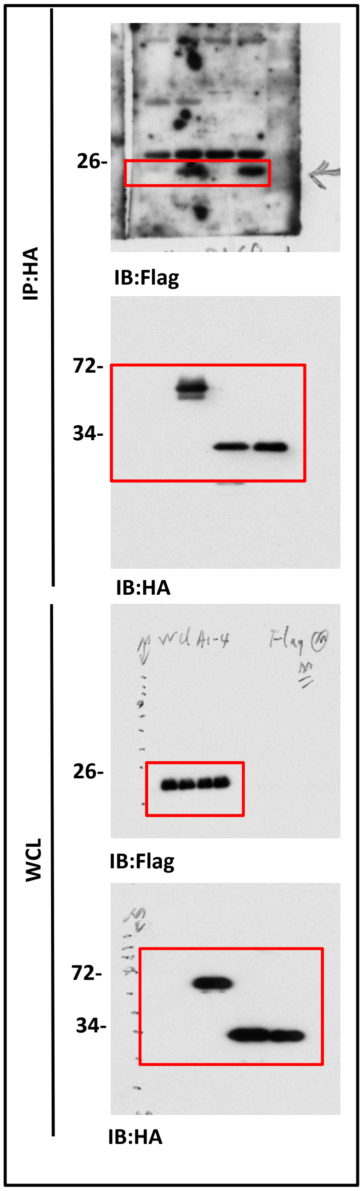

Figure 3l

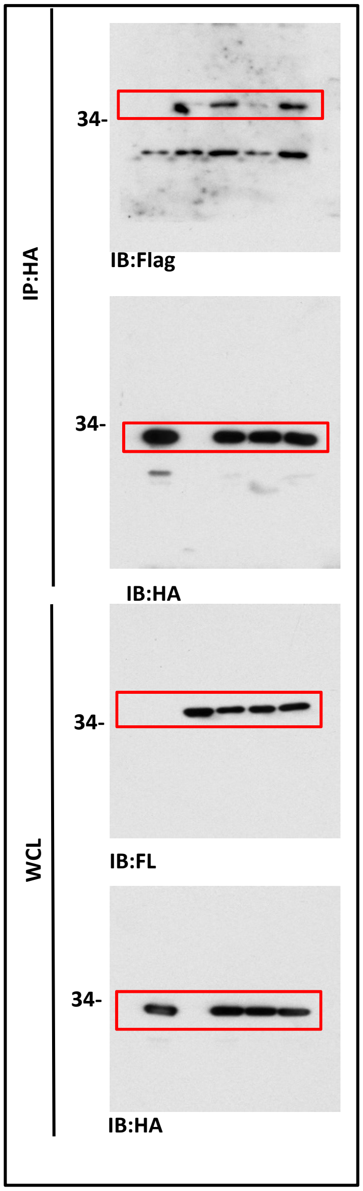

Figure 3m

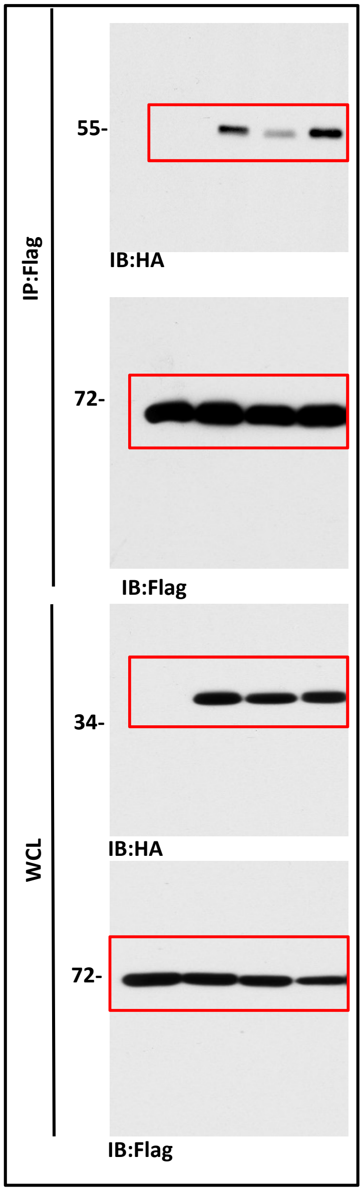

Uncropped immunoblots from Figure 3

Figure 4a

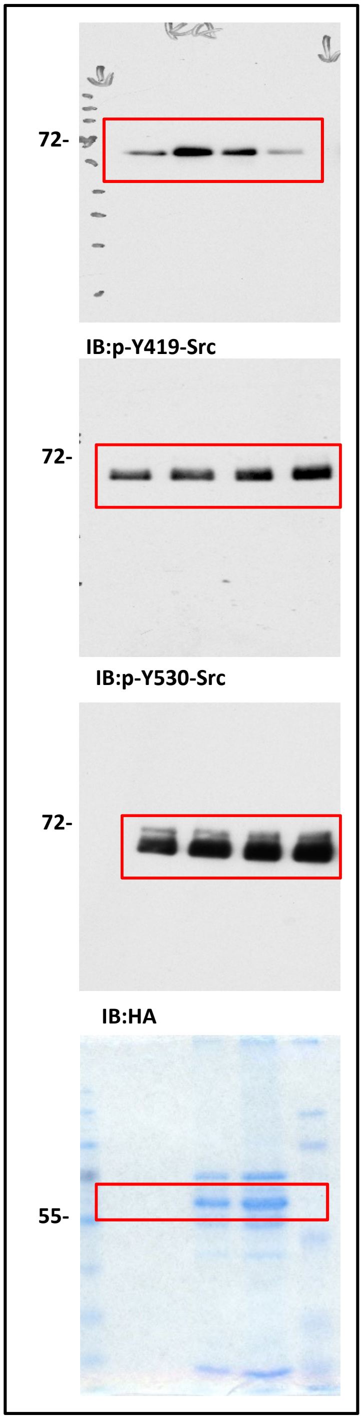

Figure 4b

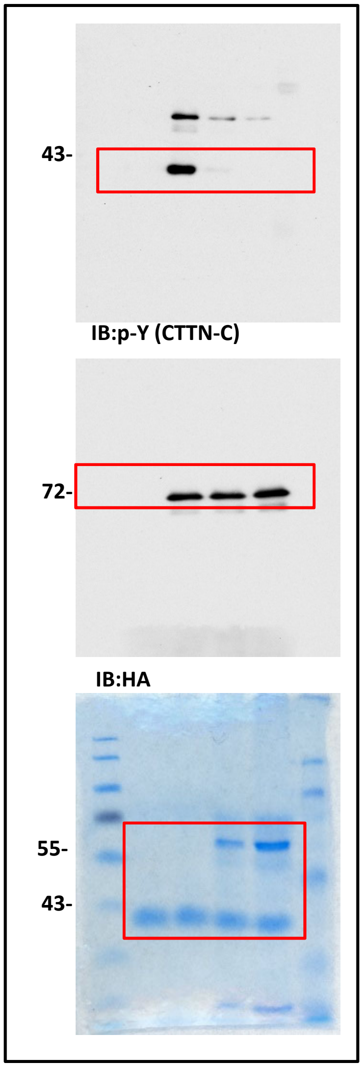

Figure 4d

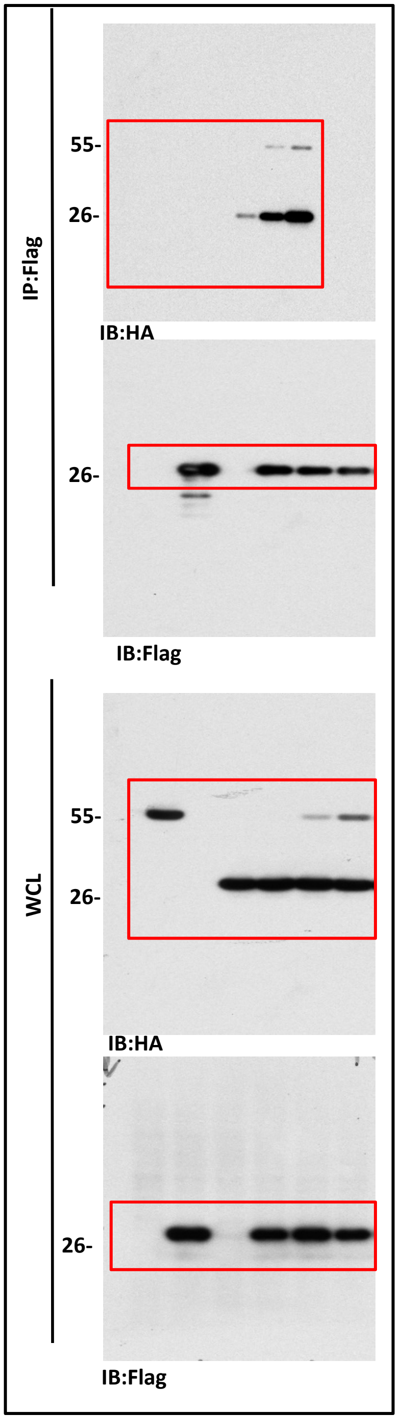

Figure 4e

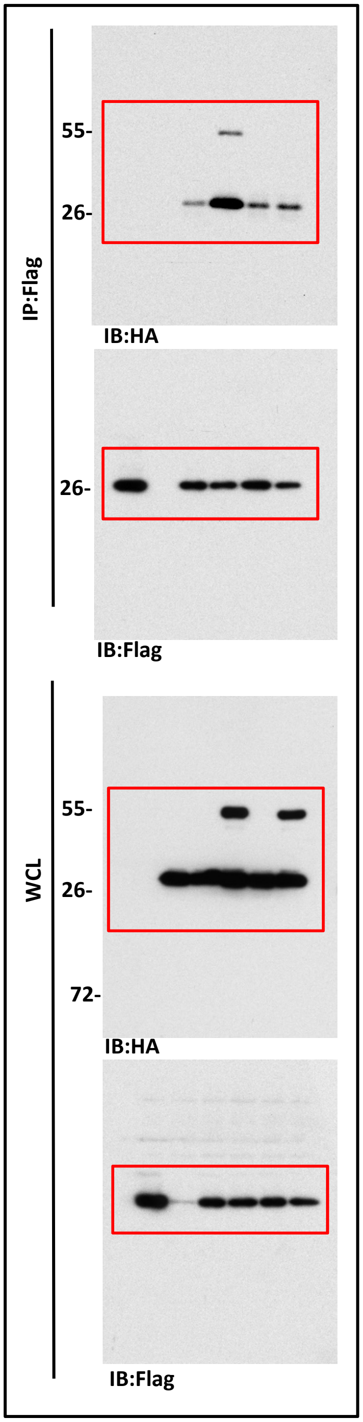

Uncropped immunoblots from Figure 4

Figure 4g

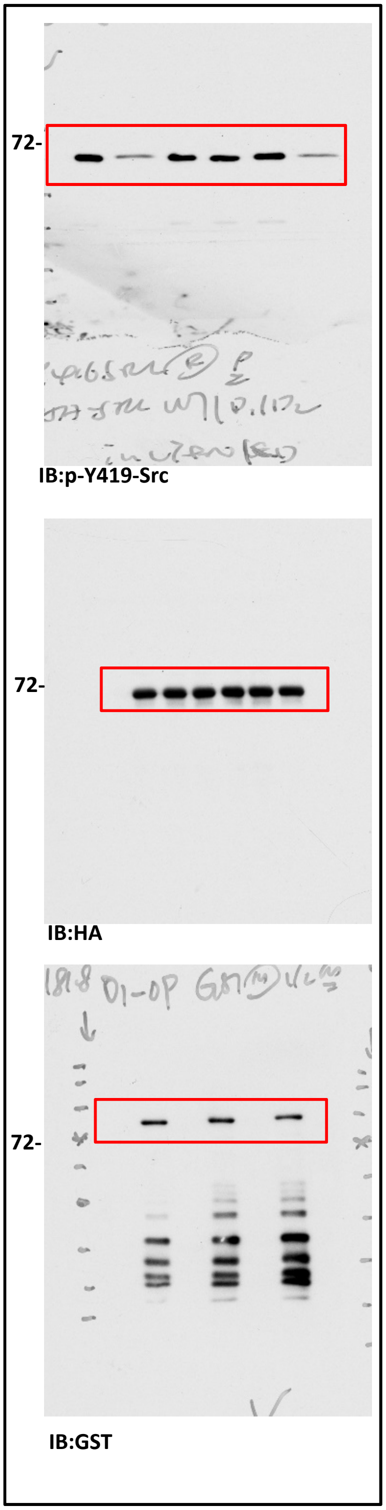

Figure 4h

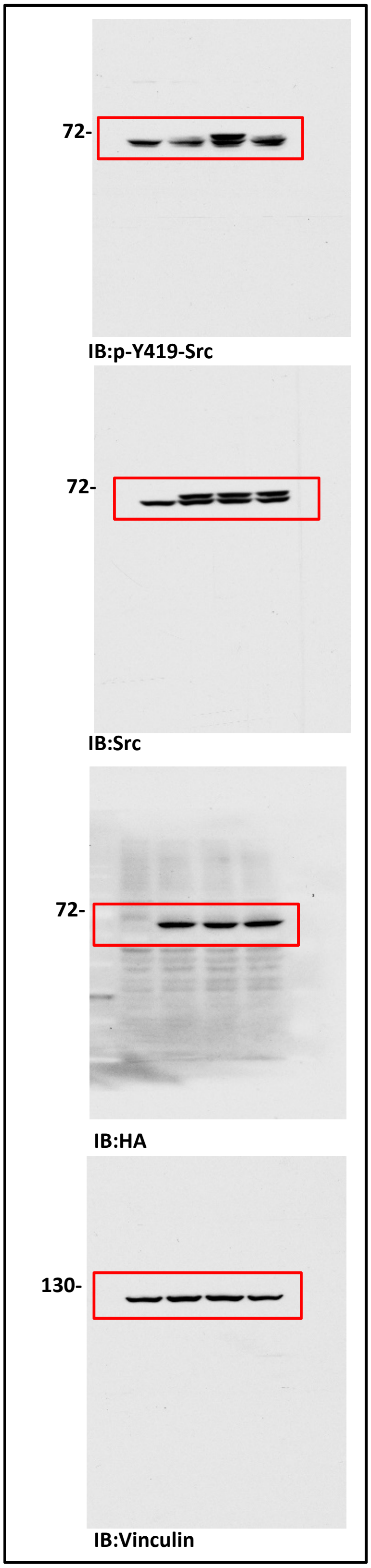

Figure 4i

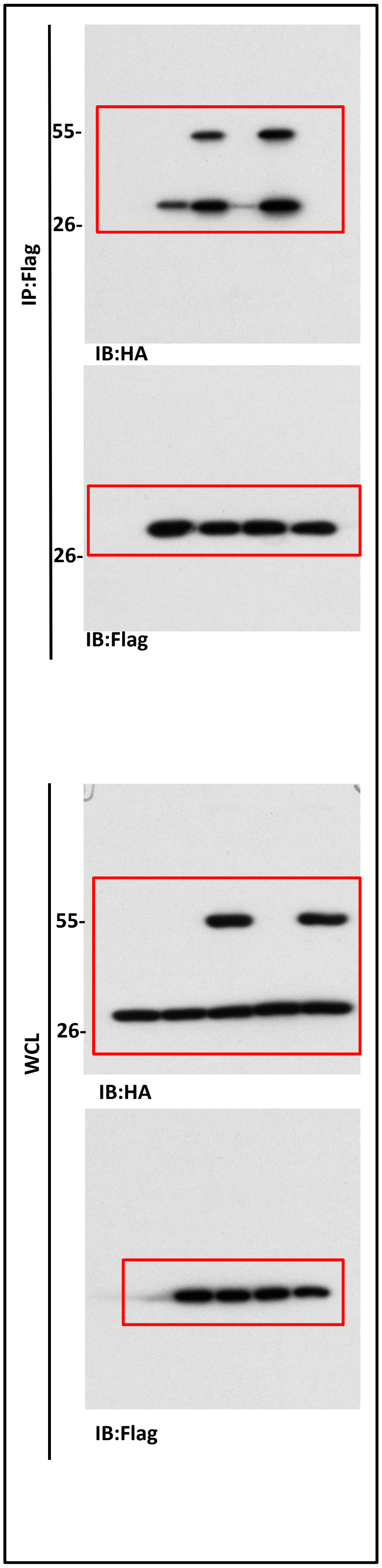

Uncropped immunoblots from Figure 4

Figure 4j

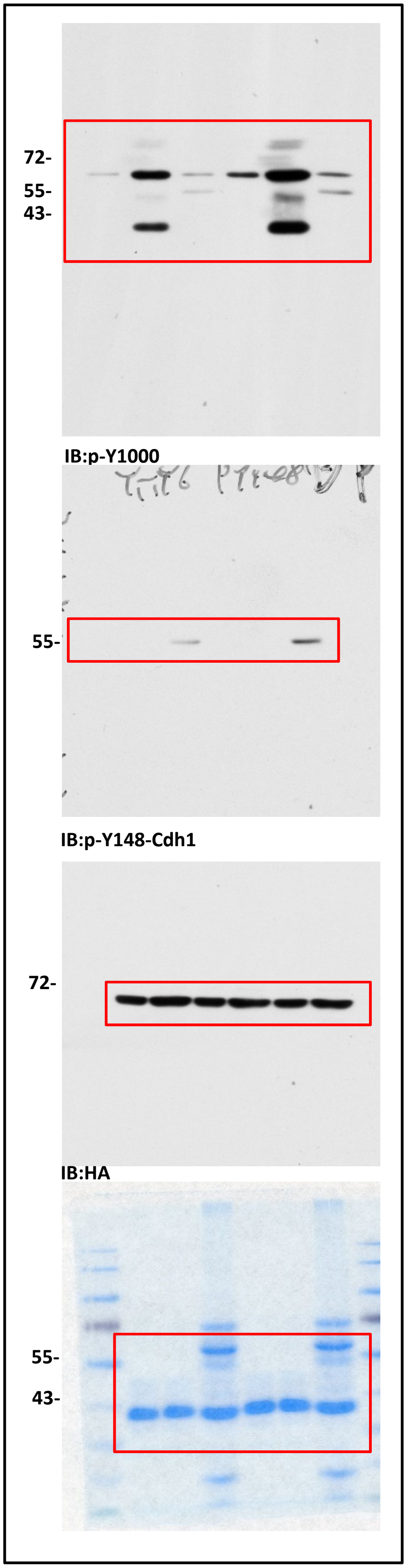

Figure 4k

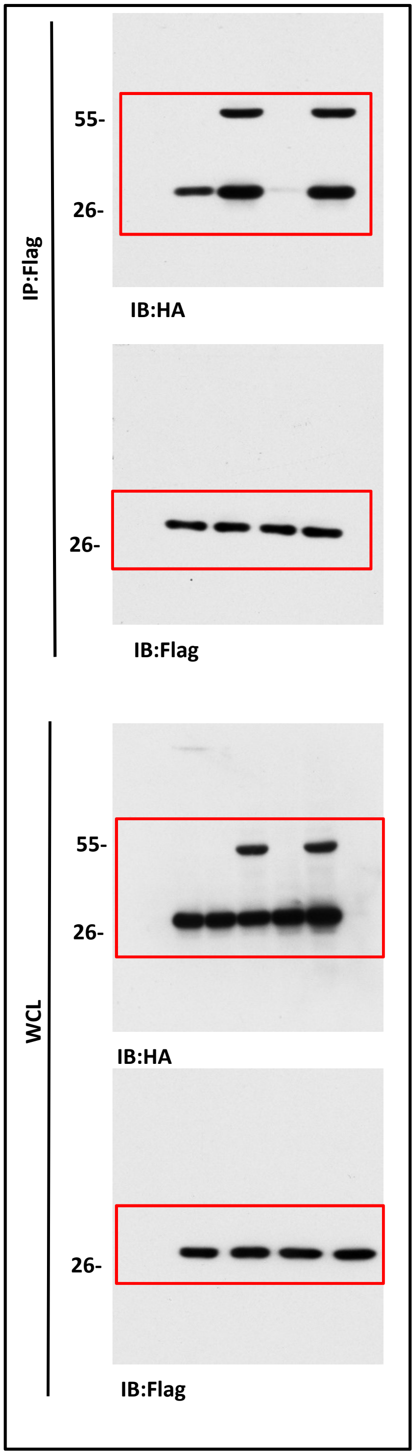

Uncropped immunoblots from Figure 4

Figure 5a

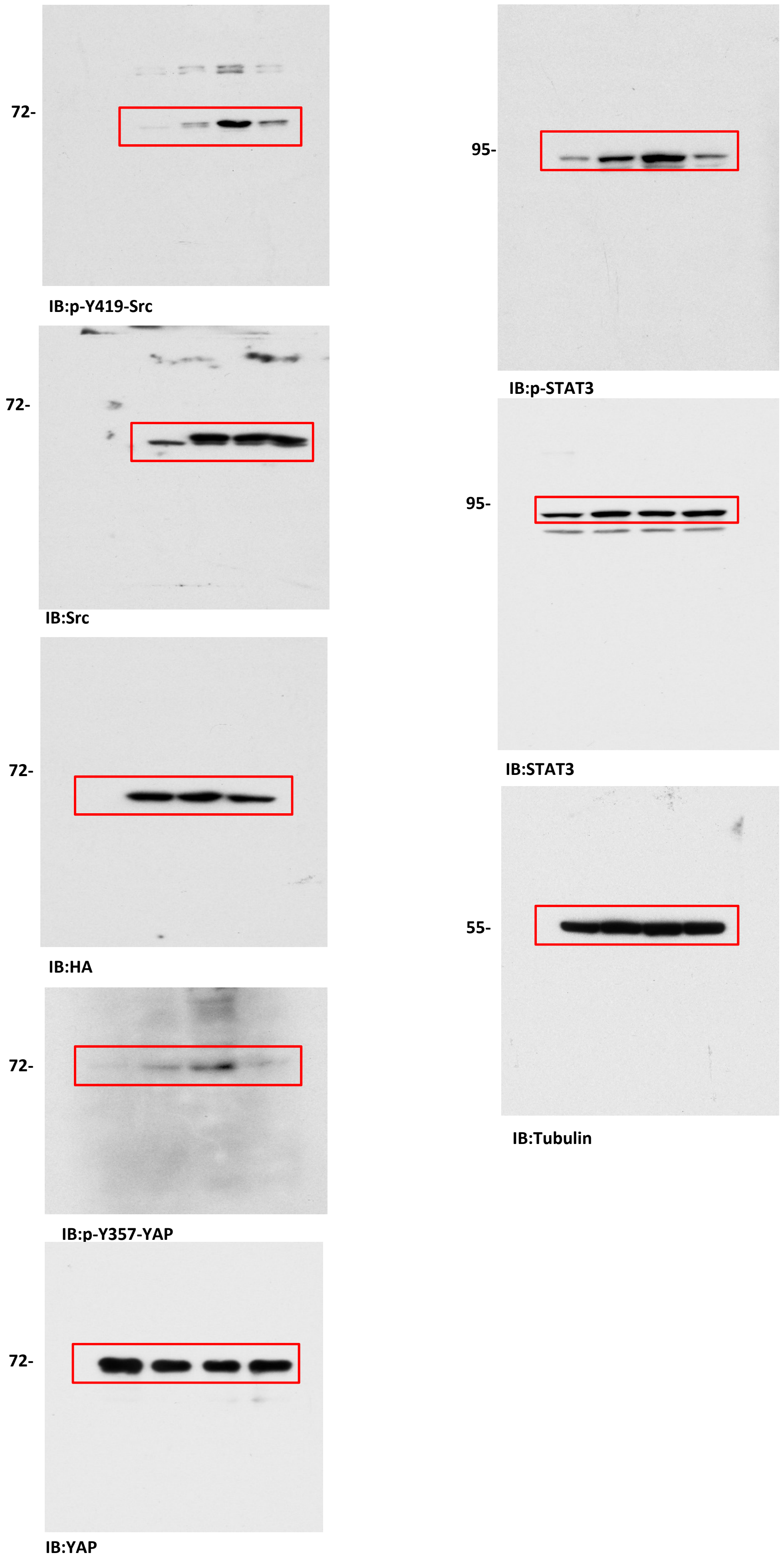

Uncropped immunoblots from Figure 5

Figure 6a Figure 6b Figure 6c Figure 6d

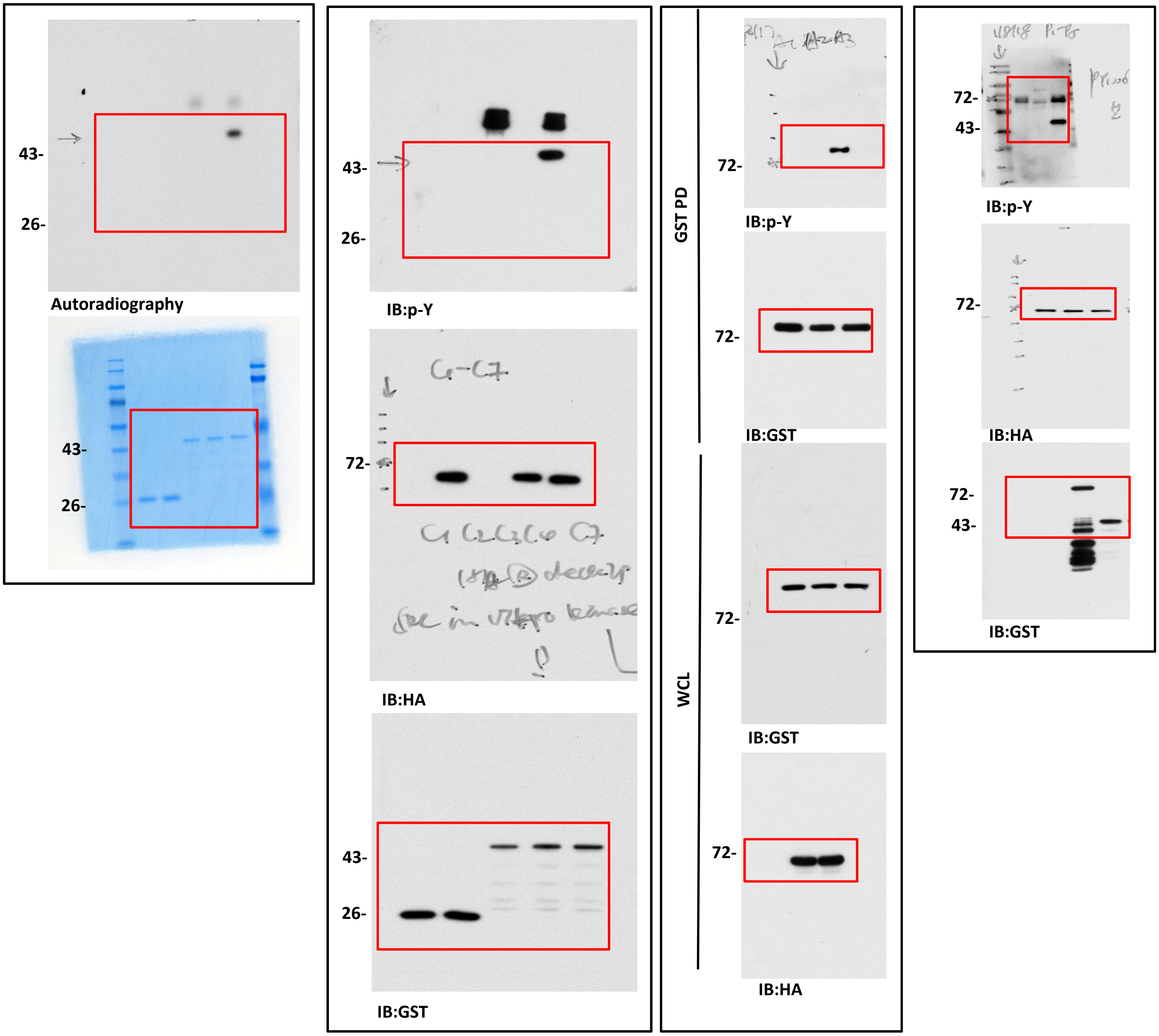

Uncropped immunoblots from Figure 6

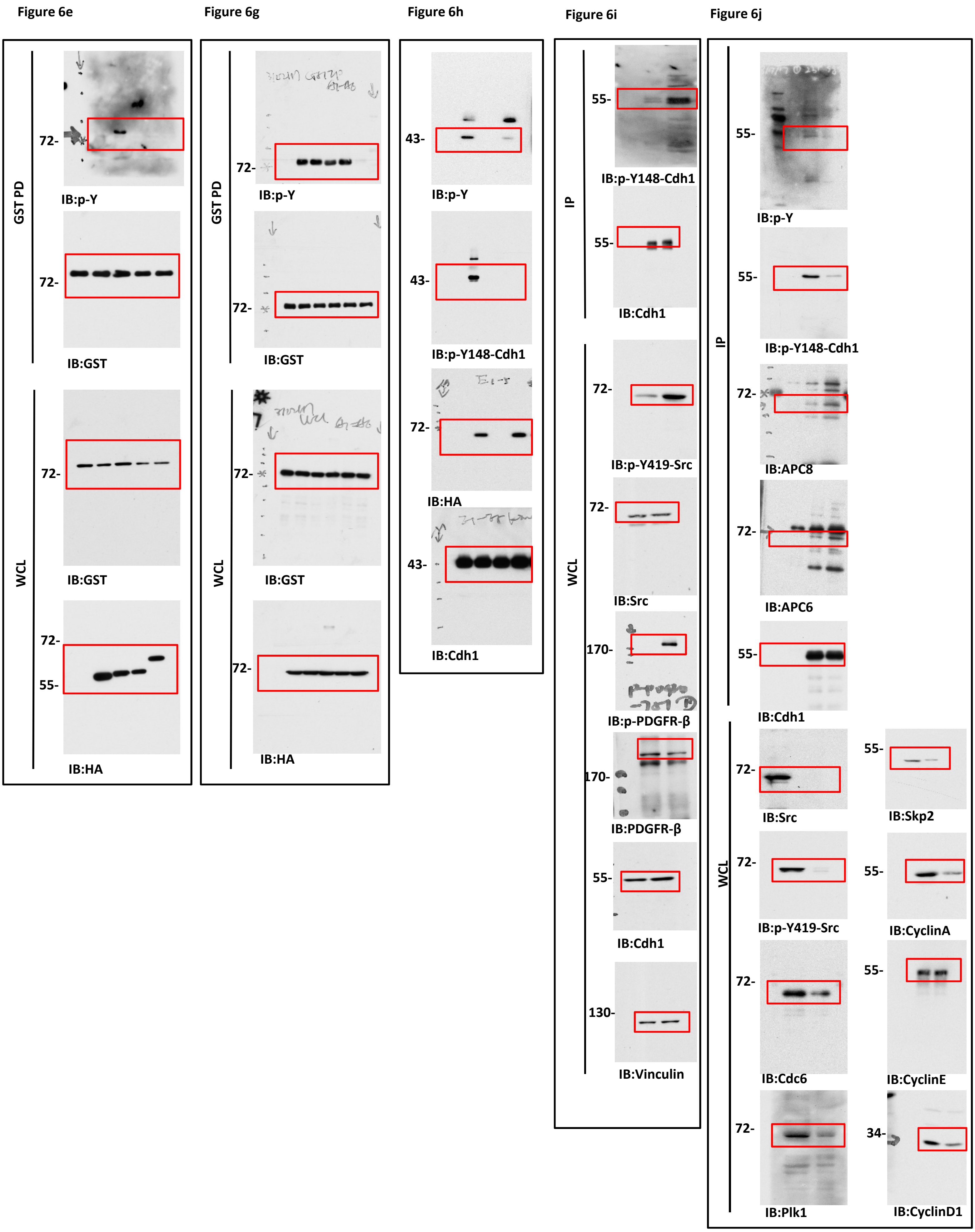

Uncropped immunoblots from Figure 6

Figure 6j

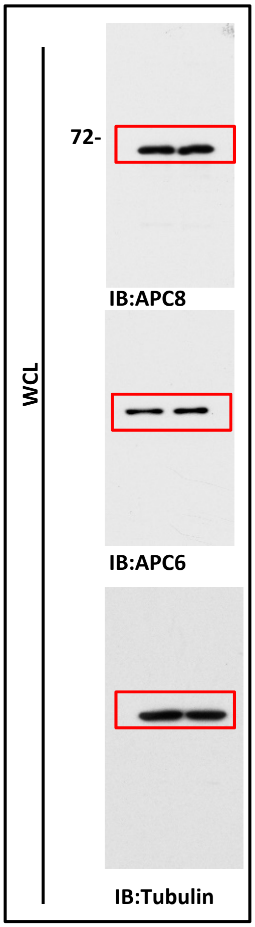

Figure 6k

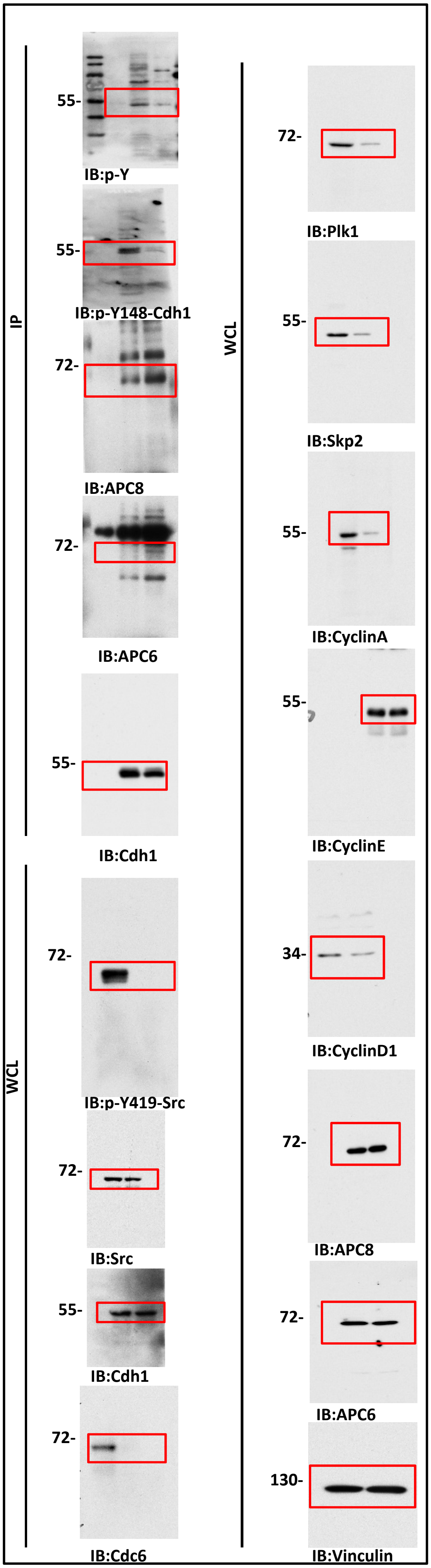

Figure 6l

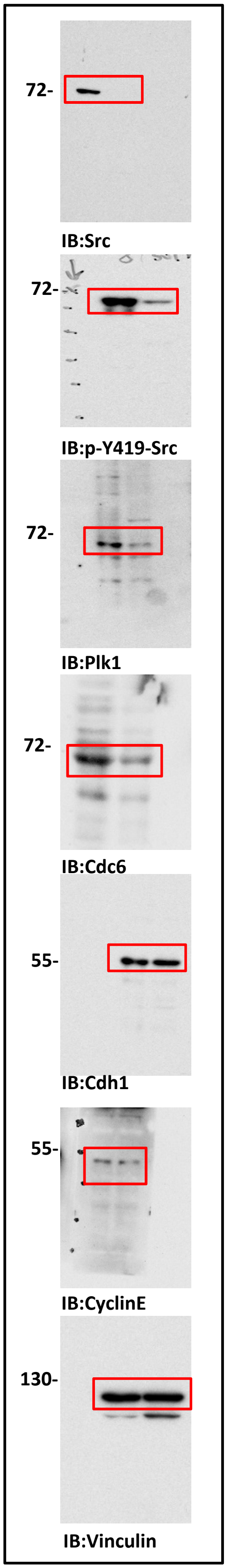

Uncropped immunoblots from Figure 6

Figure 7b

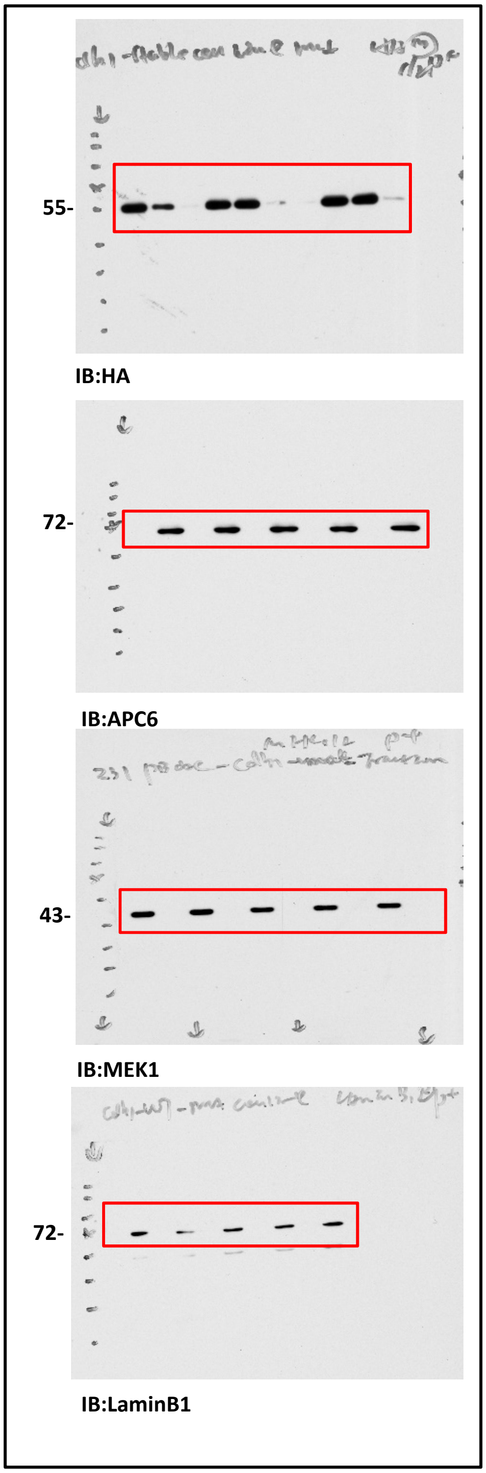

Figure 7c

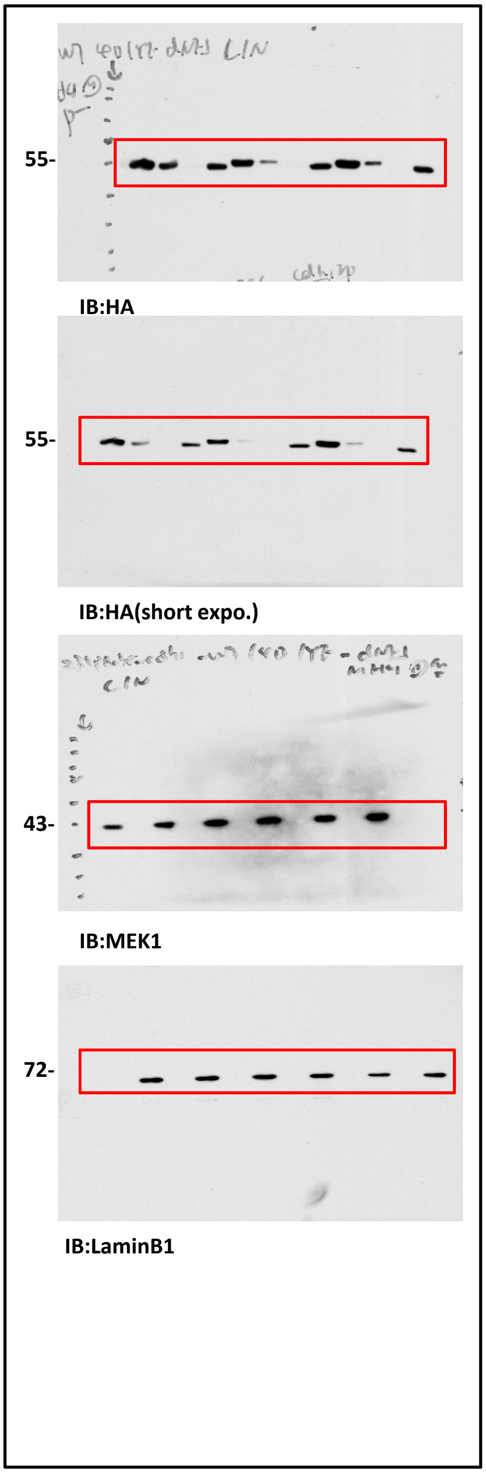

Figure 7d

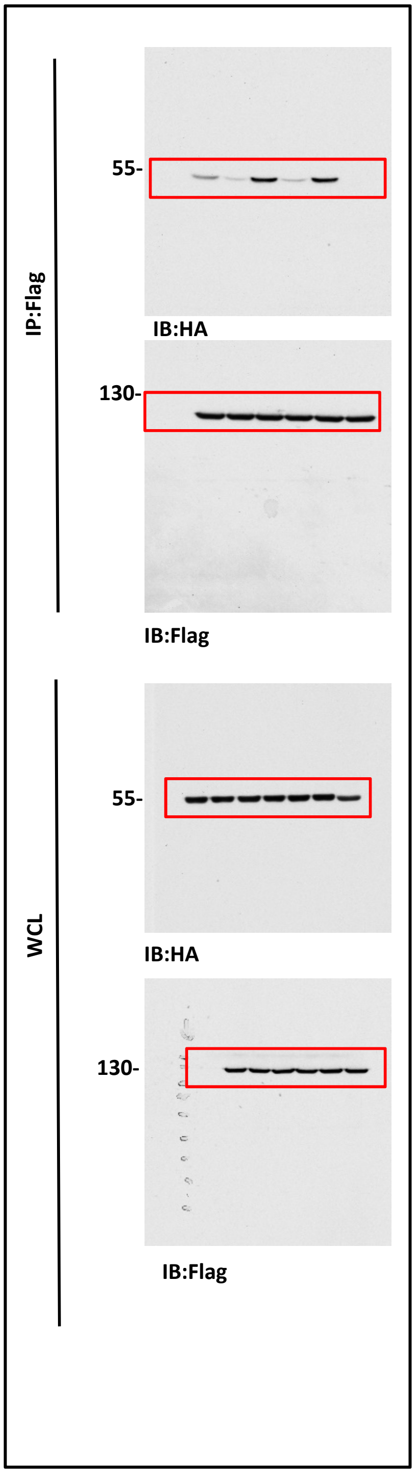

Uncropped immunoblots from Figure 7

Figure 7e

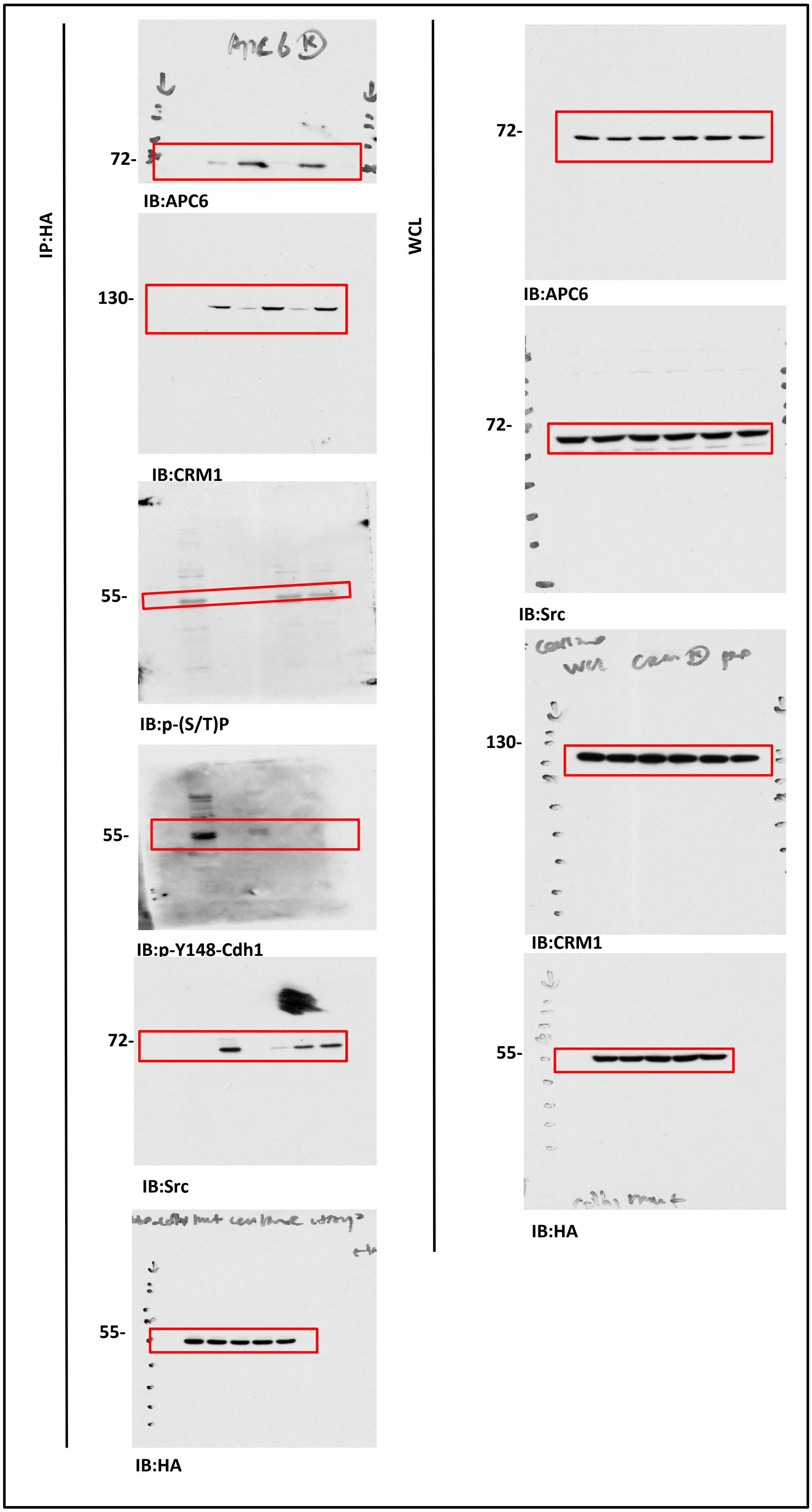

Figure 7f

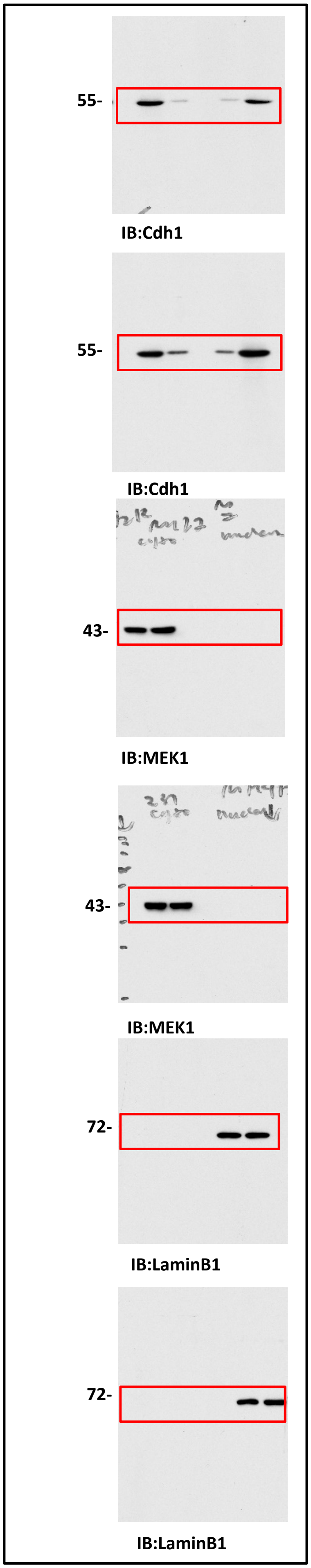

Uncropped immunoblots from Figure 7

Figure 7g

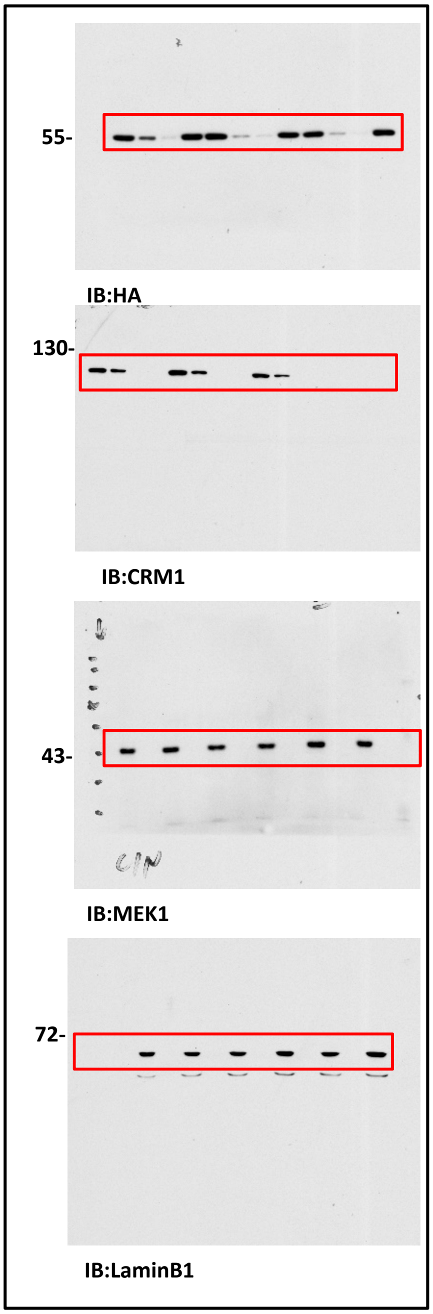

Figure 7h

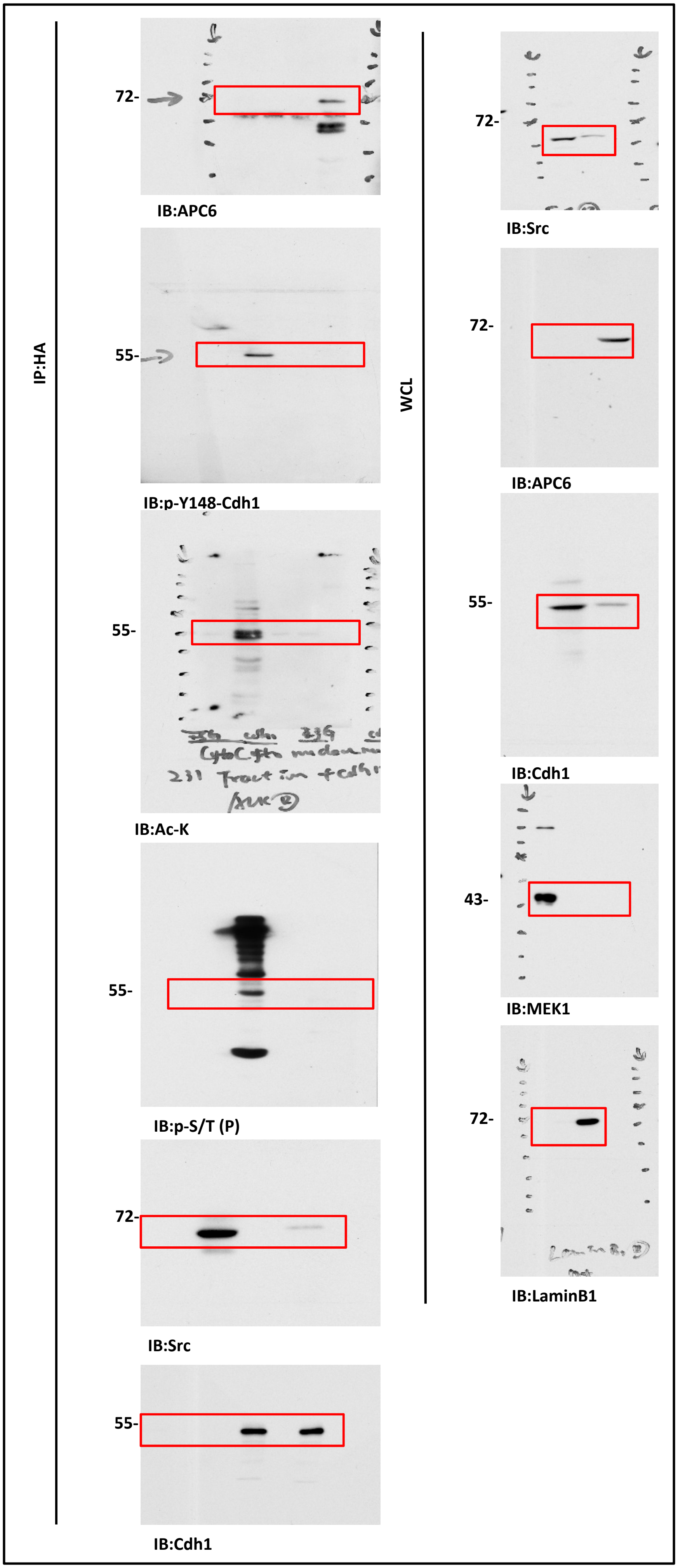

Uncropped immunoblots from Figure 7

Figure 8a

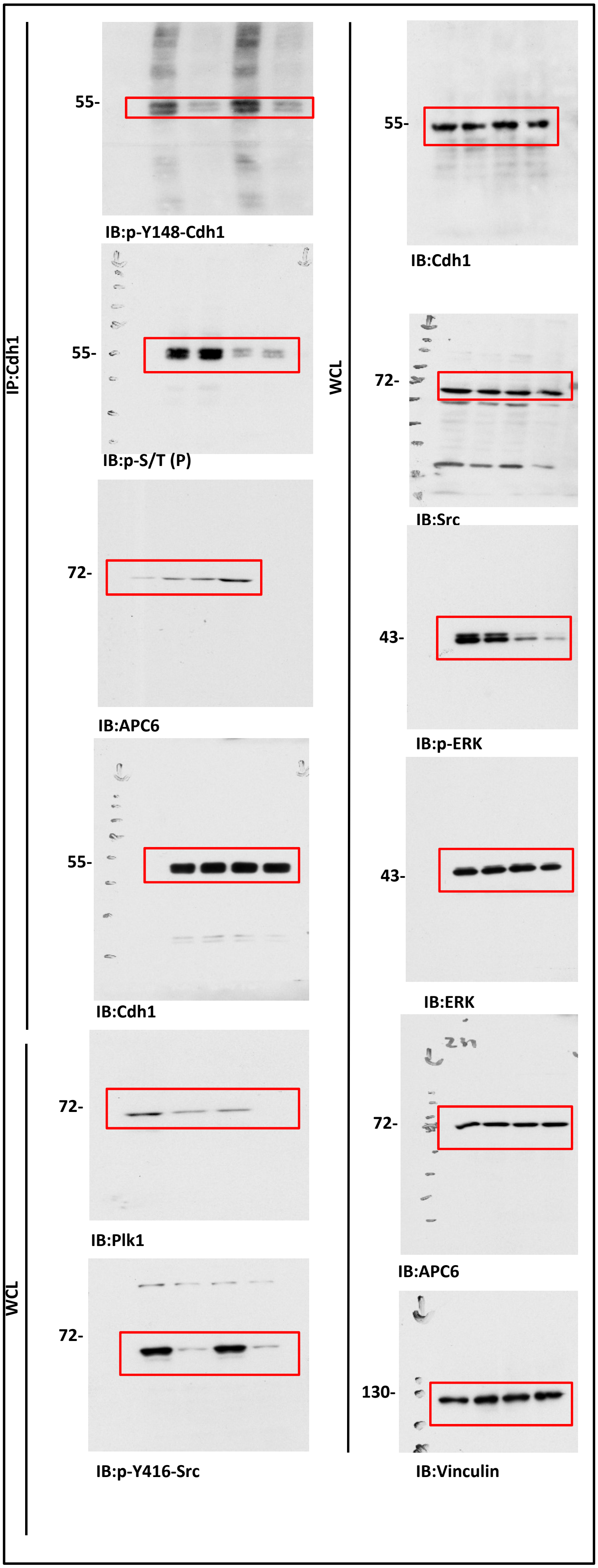

Figure 8b

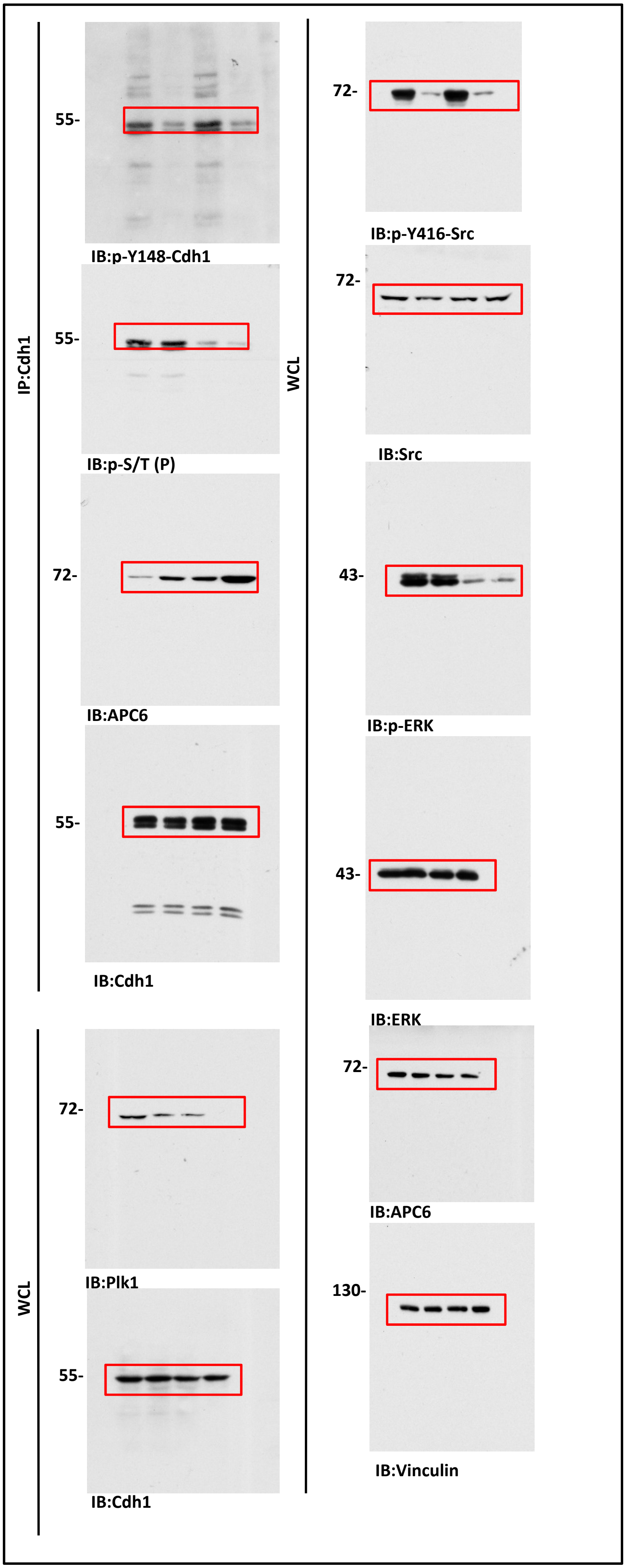

Uncropped immunoblots from Figure 8

Figure 8f

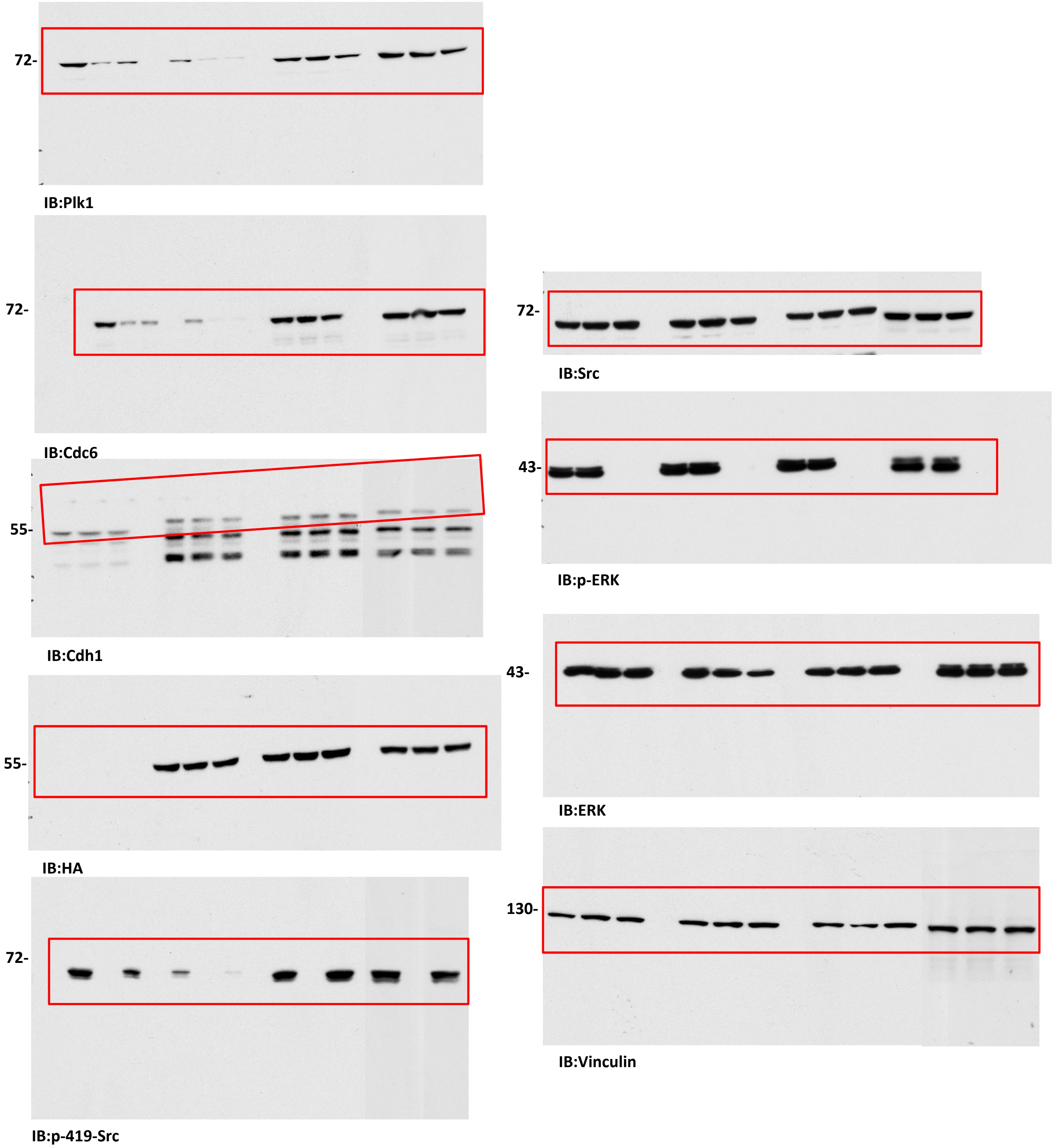

Uncropped immunoblots from Figure 8

Supplementary Figure 1a

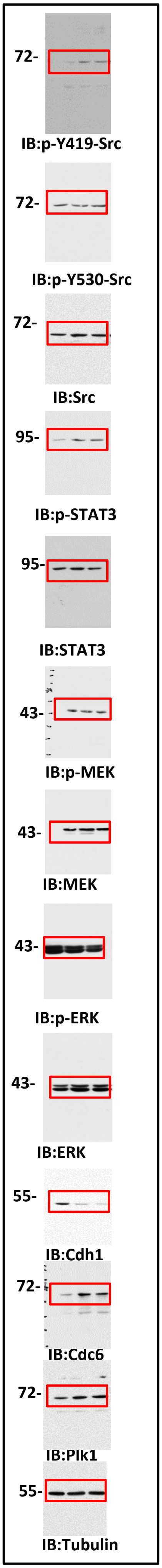

Supplementary Figure 1b

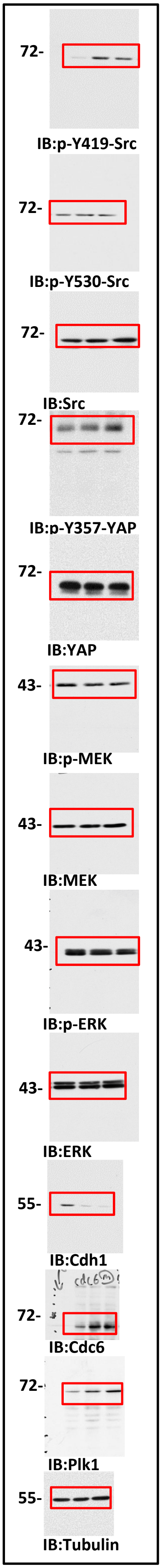

Supplementary Figure 1c

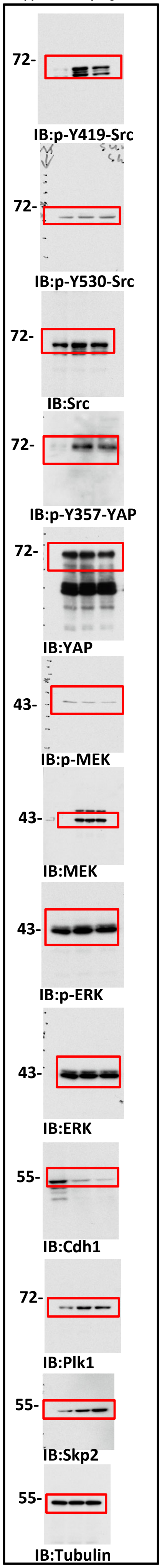

Supplementary Figure 1e

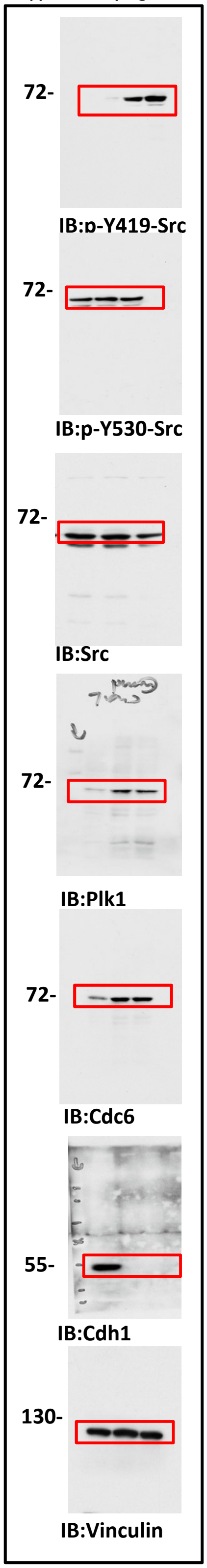

Supplementary Figure 1f

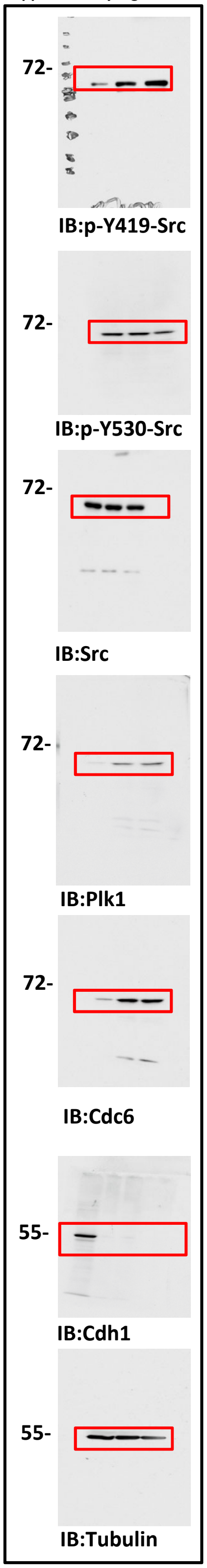

Supplementary Figure 1d

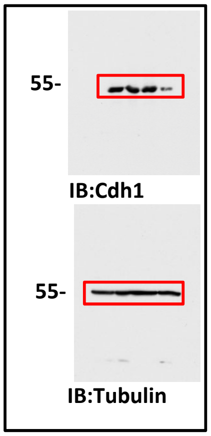

Uncropped immunoblots from Supplementary Figure 1

Supplementary Figure 1g

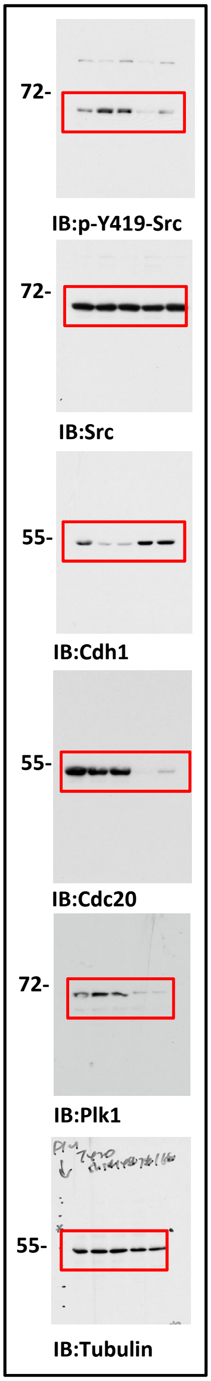

Supplementary Figure 1h

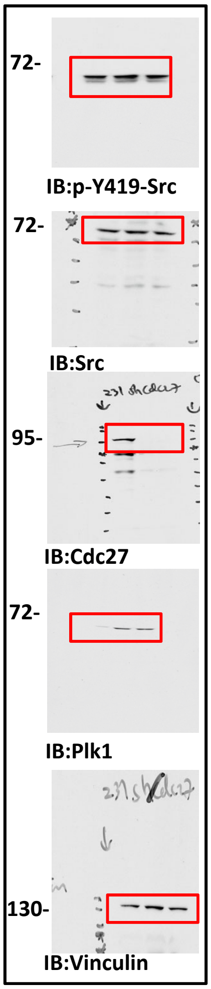

Supplementary Figure 1i

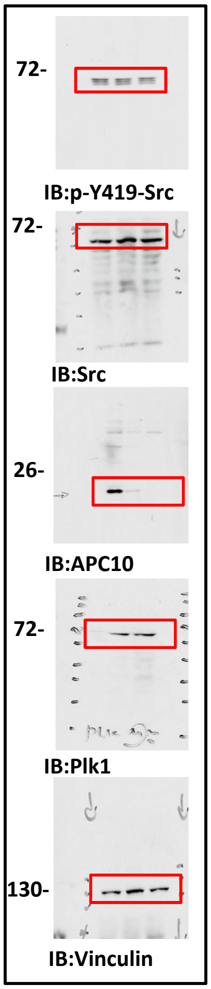

Supplementary Figure 1j

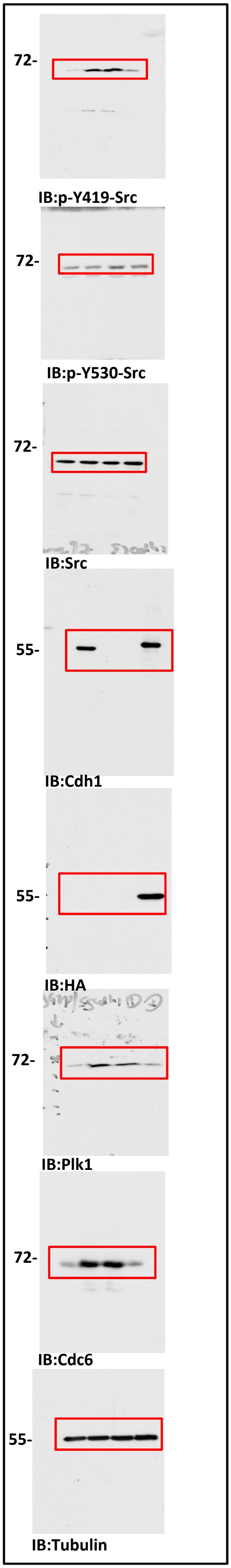

Supplementary Figure 1k

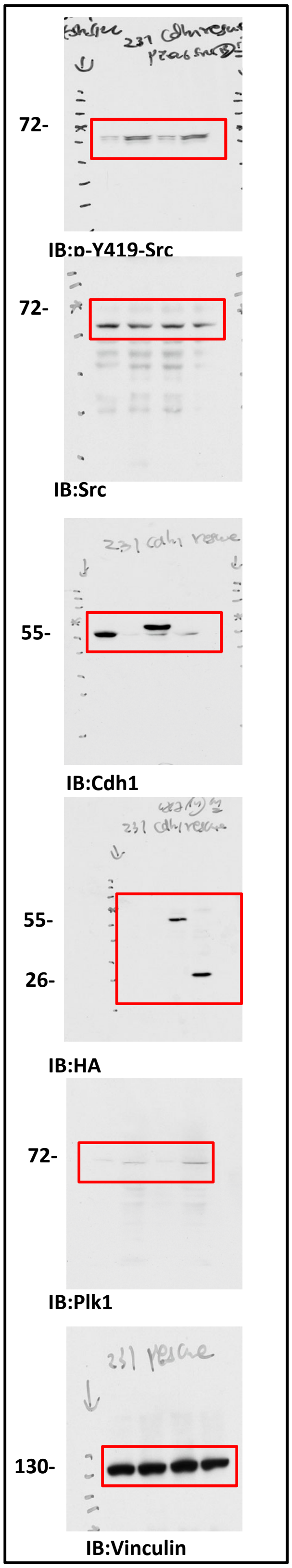

Uncropped immunoblots from Supplementary Figure 1

Supplementary Figure 1l

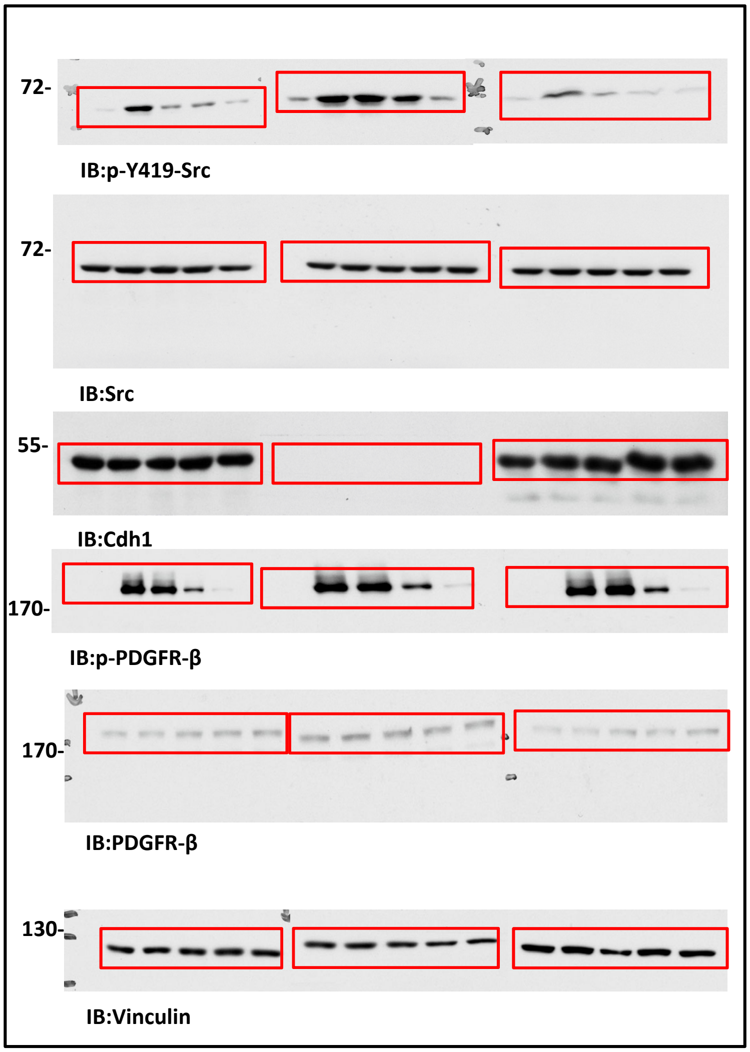

Supplementary Figure 1m

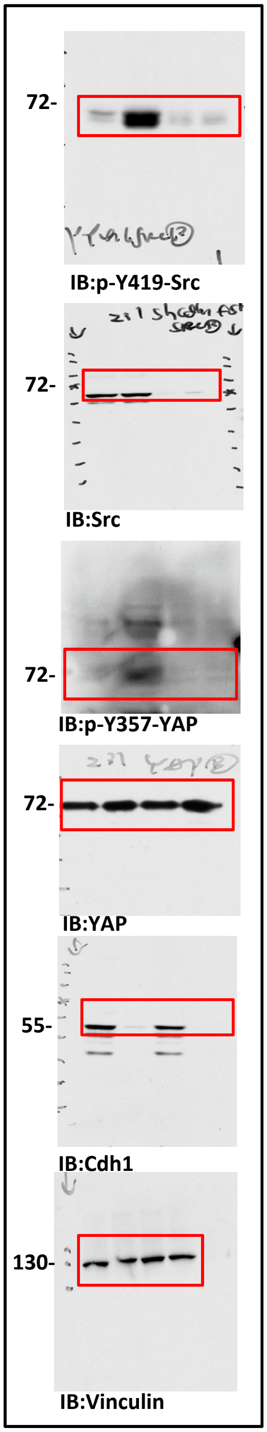

Uncropped immunoblots from Supplementary Figure 1

Supplementary Figure 1n

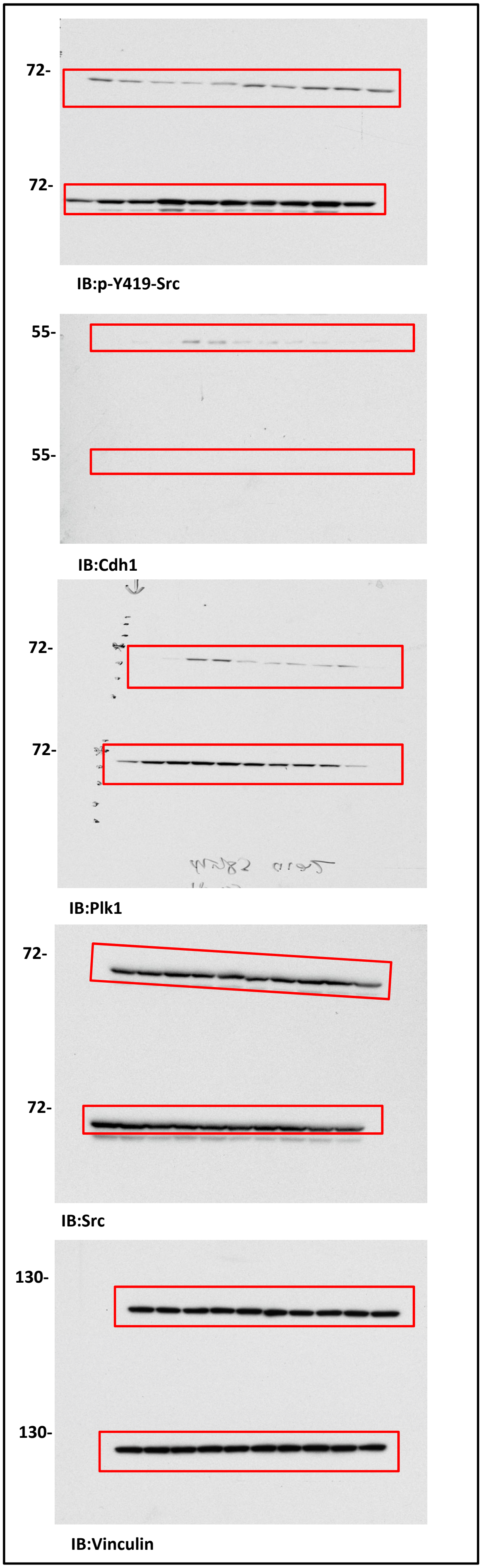

Supplementary Figure 1p

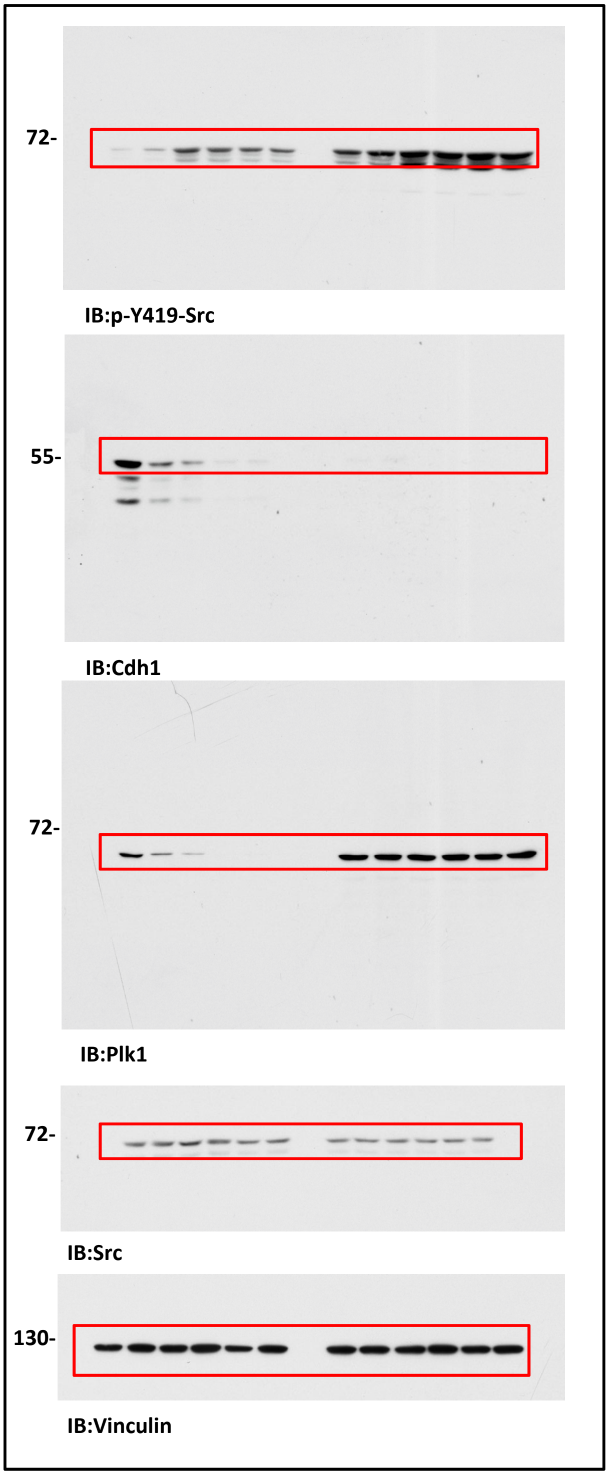

Uncropped immunoblots from Supplementary Figure 1

Supplementary Figure 3a

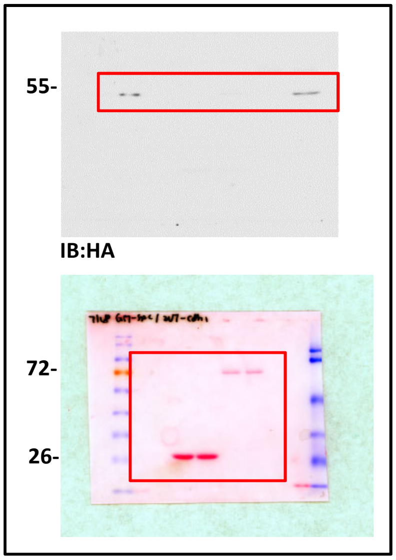

Supplementary Figure 3b

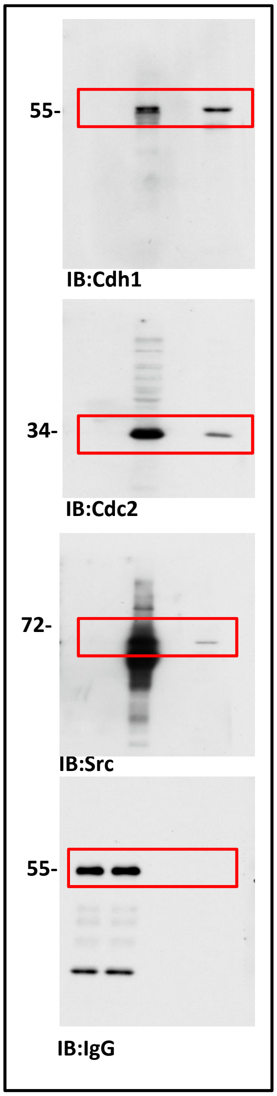

Supplementary Figure 3c

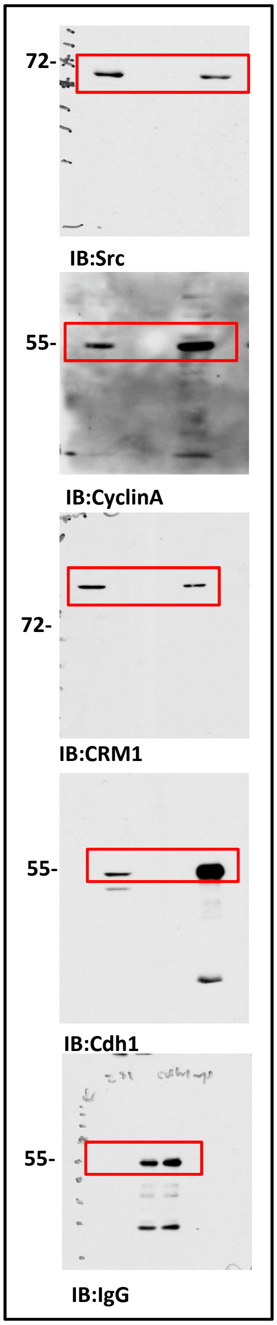

Supplementary Figure 3d

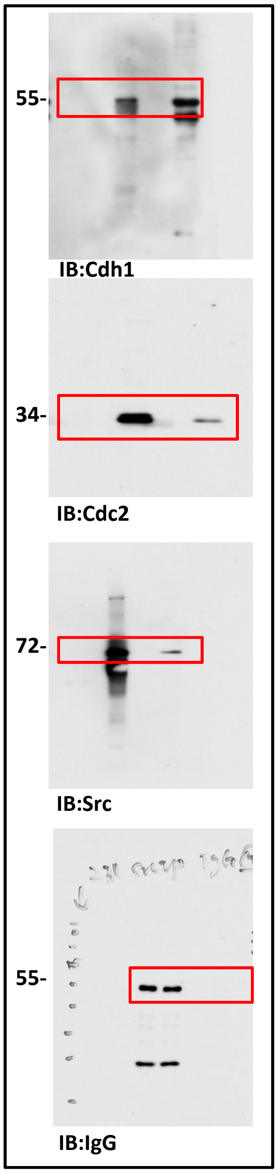

Uncropped immunoblots from Supplementary Figure 3

Supplementary Figure 3f

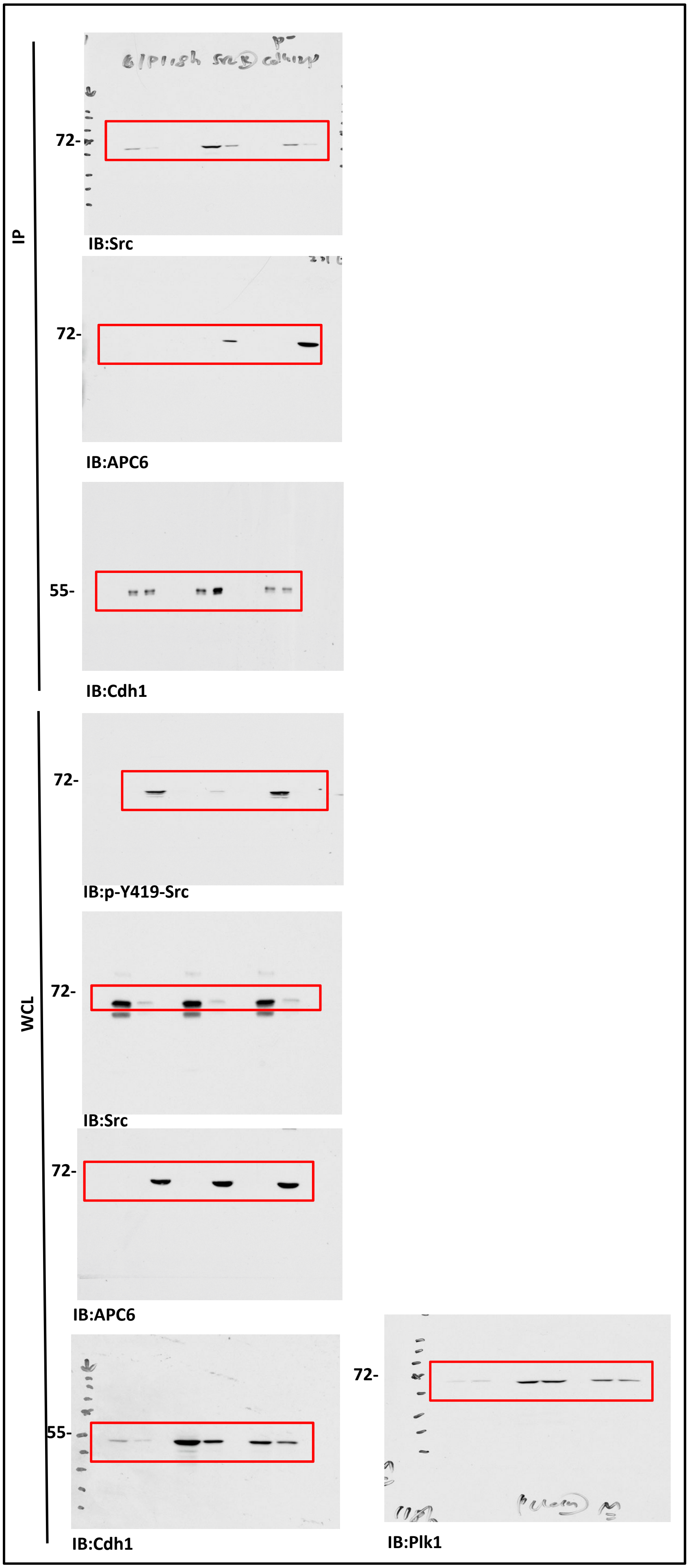

Supplementary Figure 3i

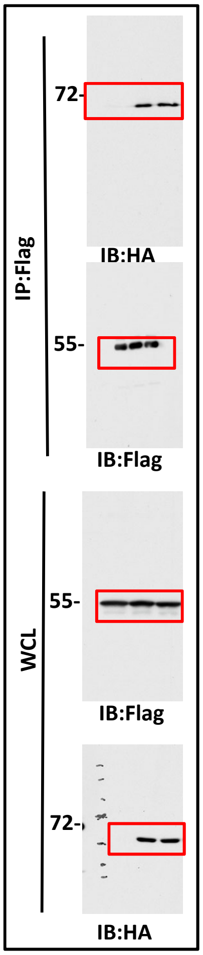

Supplementary Figure 3k

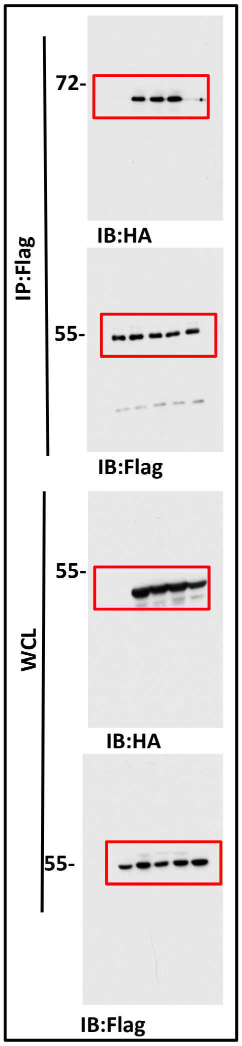

Supplementary Figure 3l

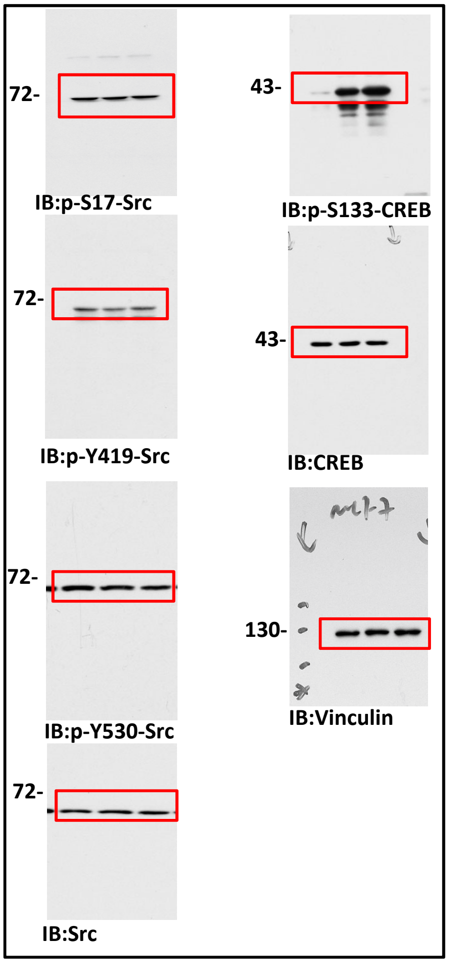

Uncropped immunoblots from Supplementary Figure 3

Supplementary Figure 3m

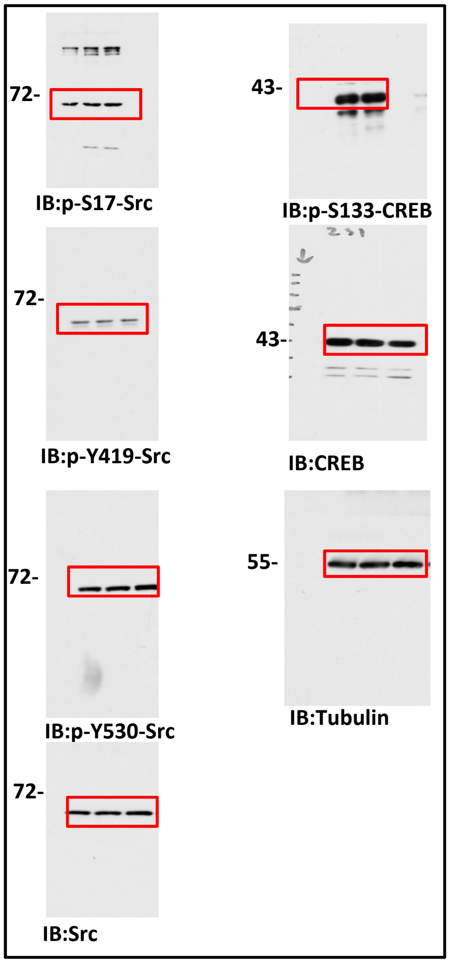

Supplementary Figure 3n

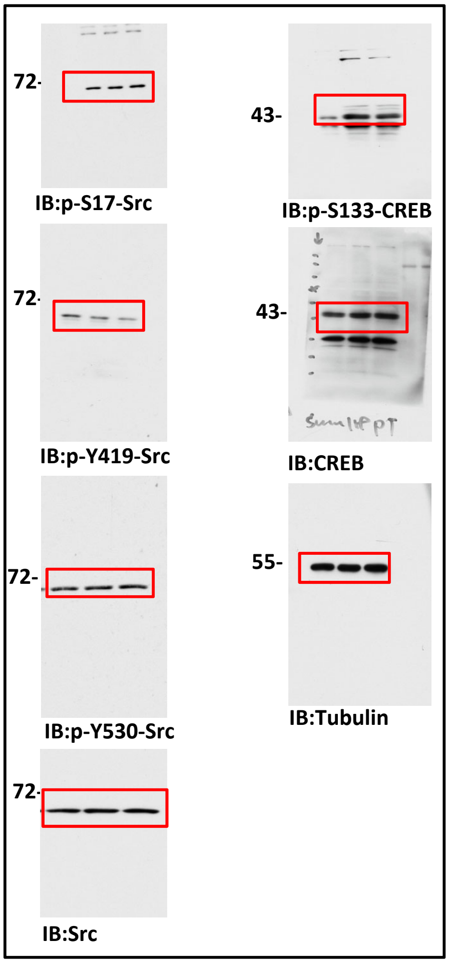

Supplementary Figure 3o

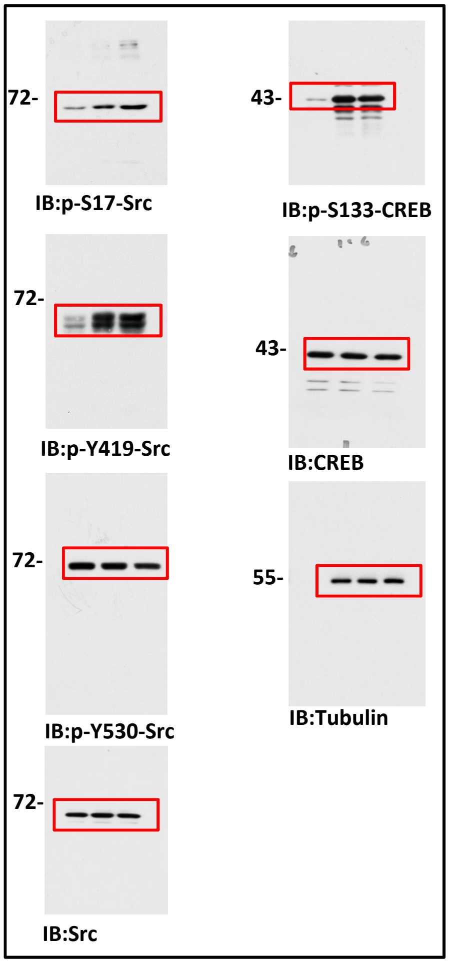

Supplementary Figure 3p

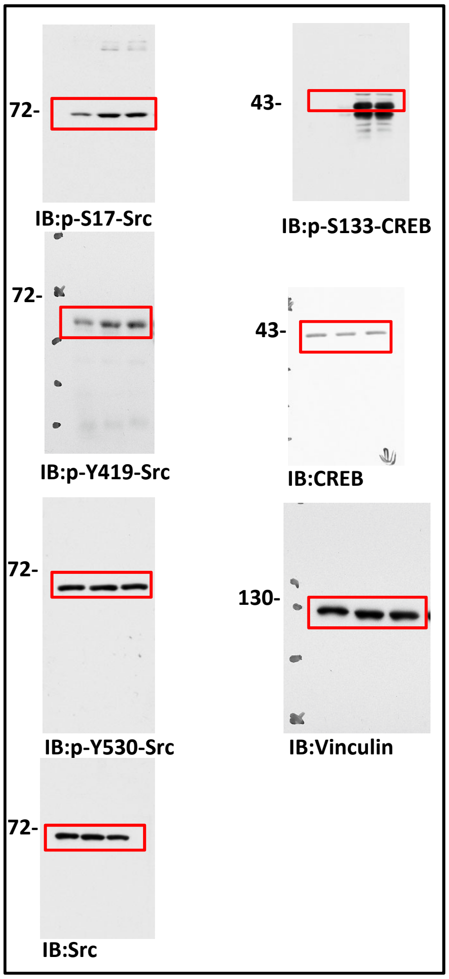

Supplementary Figure 3q

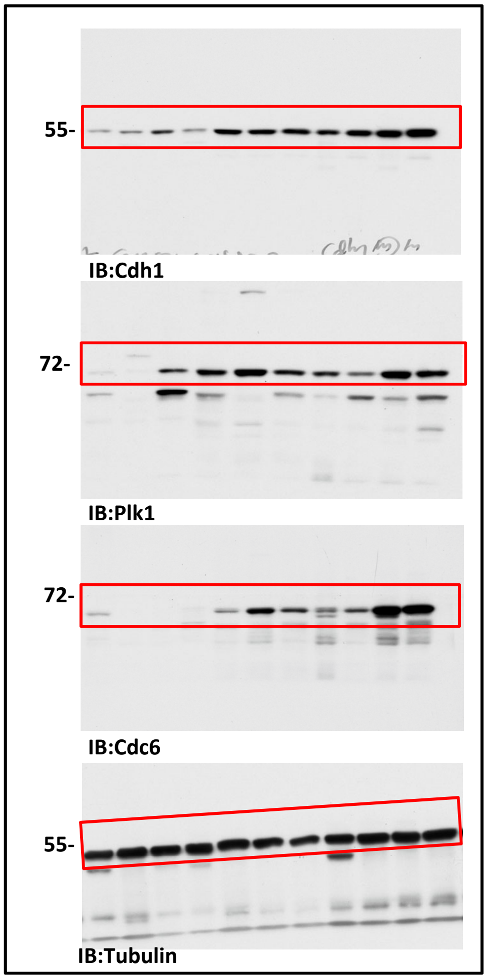

Uncropped immunoblots from Supplementary Figure 3

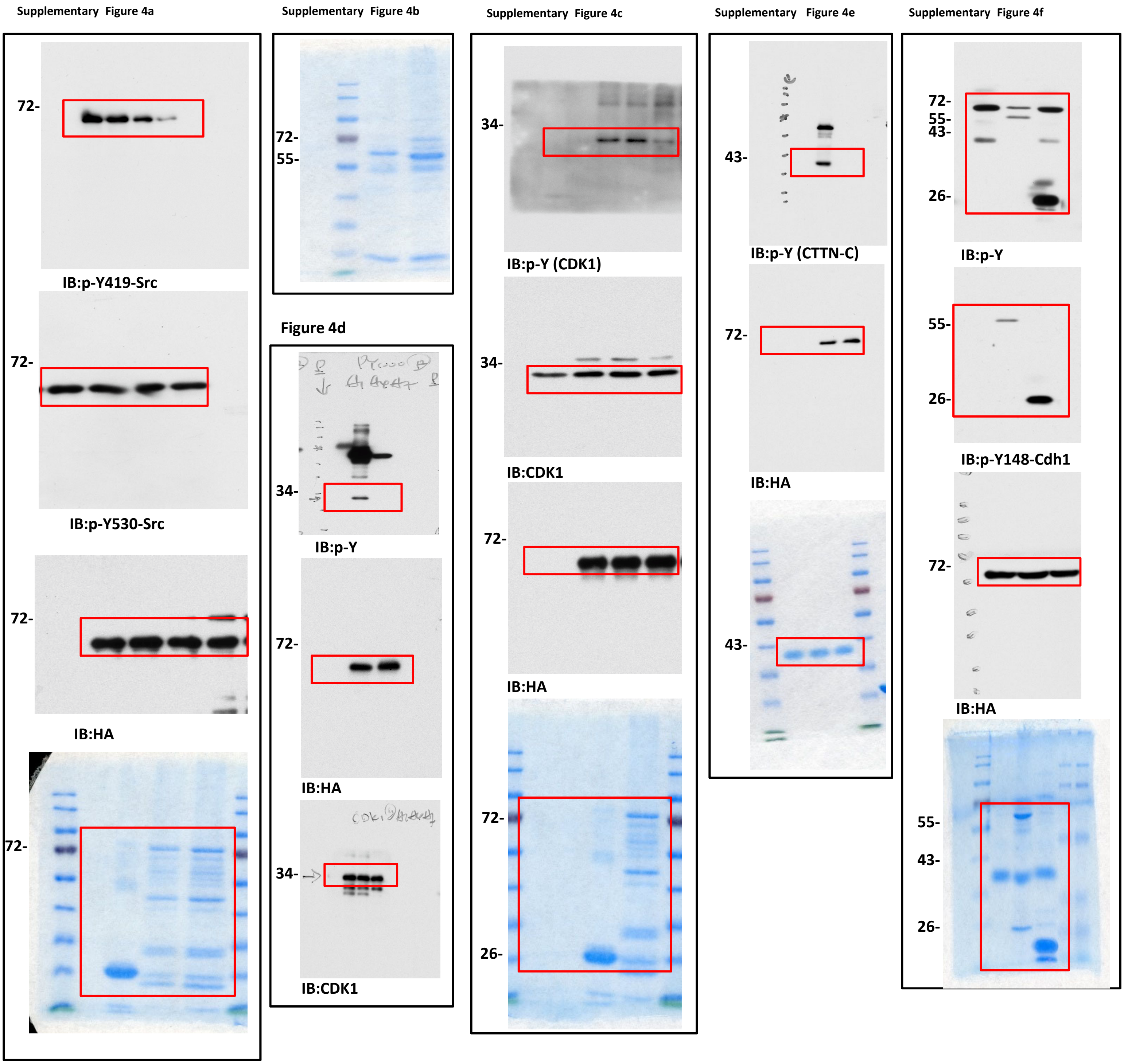

Uncropped immunoblots from Supplementary Figure 4

Supplementary Figure 4g

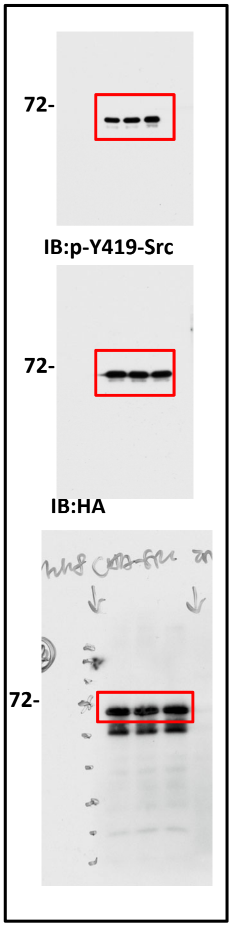

Supplementary Figure 4h

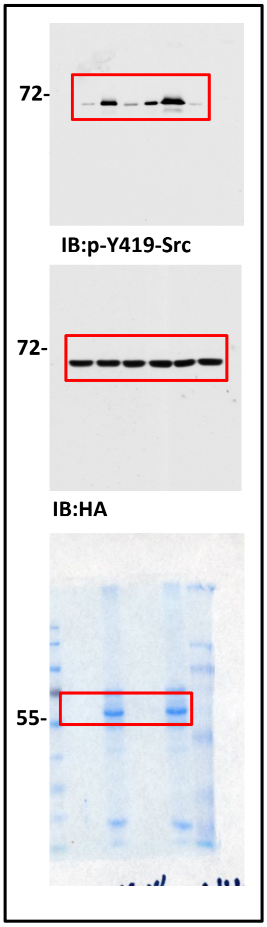

Supplementary Figure 4i

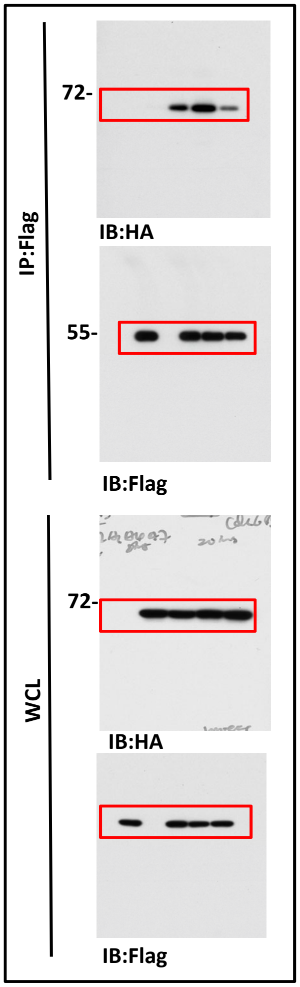

Supplementary Figure 4k

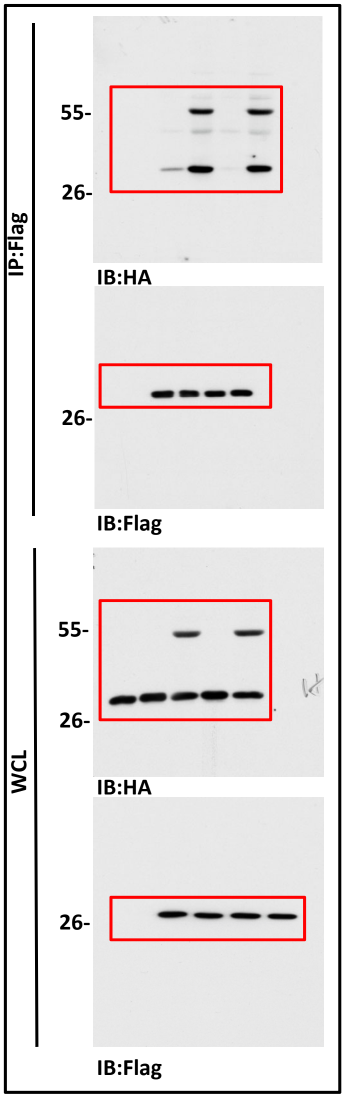

Supplementary Figure 4l

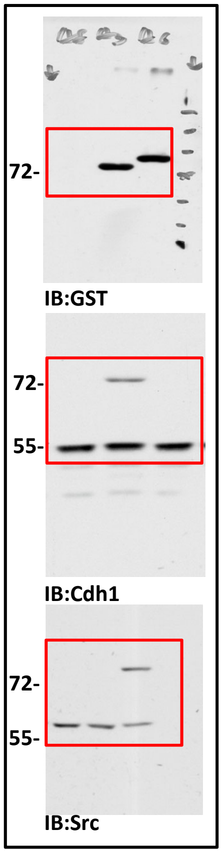

Supplementary Figure 4m

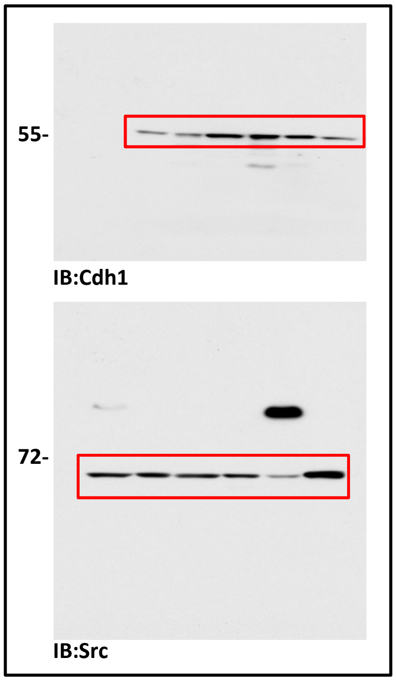

Uncropped immunoblots from Supplementary Figure 4

Supplementary Figure 5a

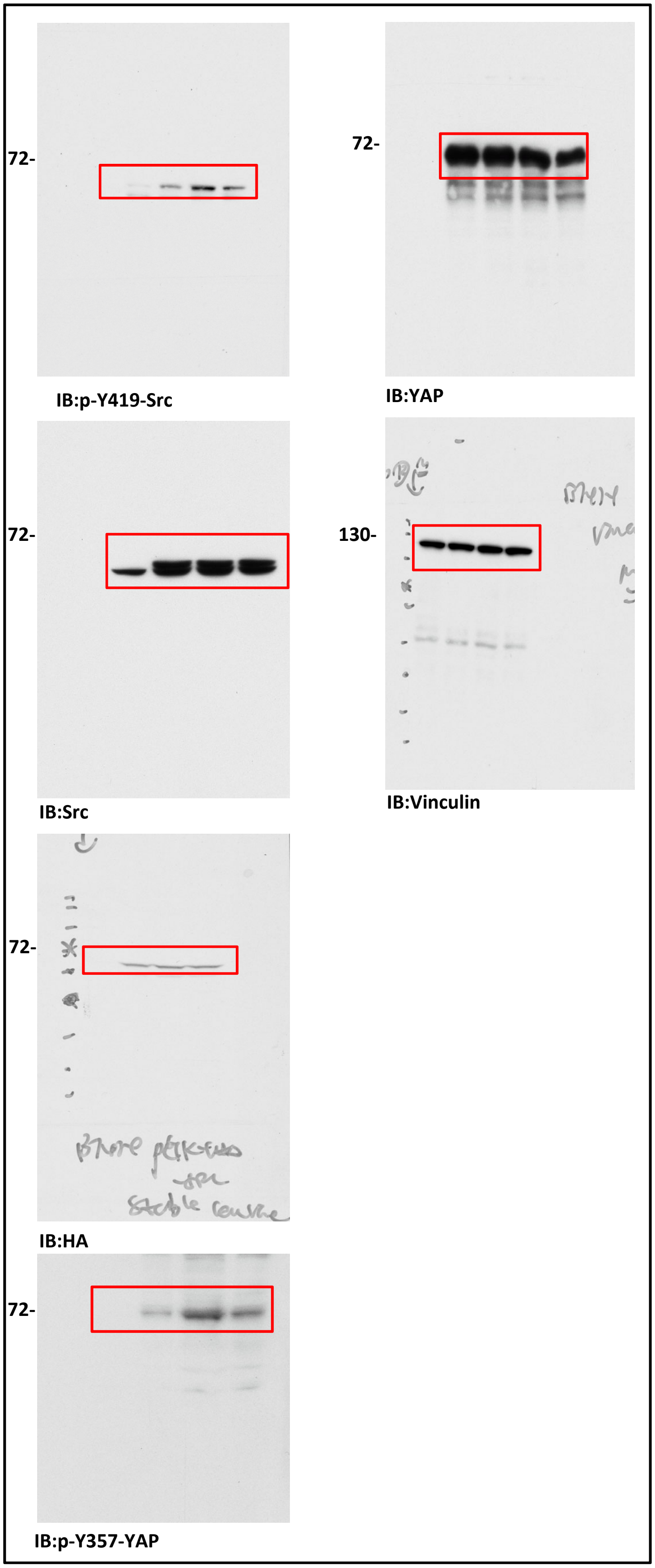

Supplementary Figure 5g

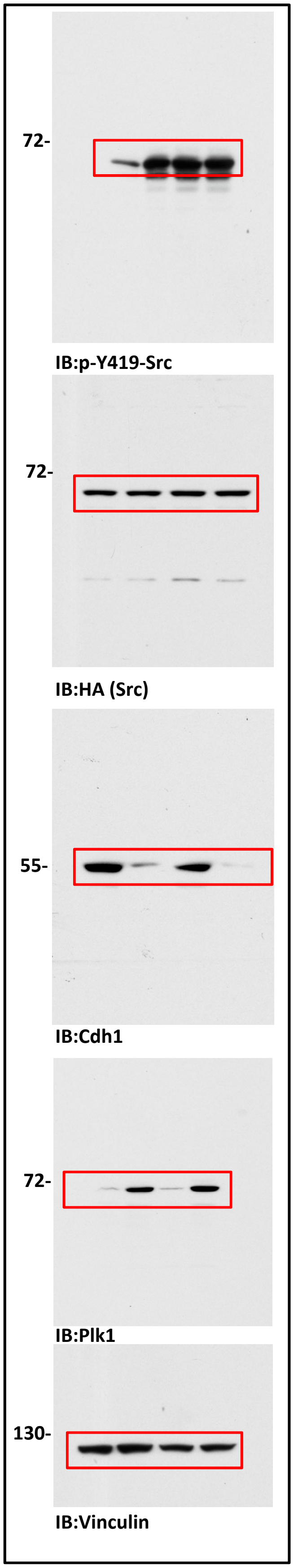

Uncropped immunoblots from Supplementary Figure 5

Supplementary Figure 6a

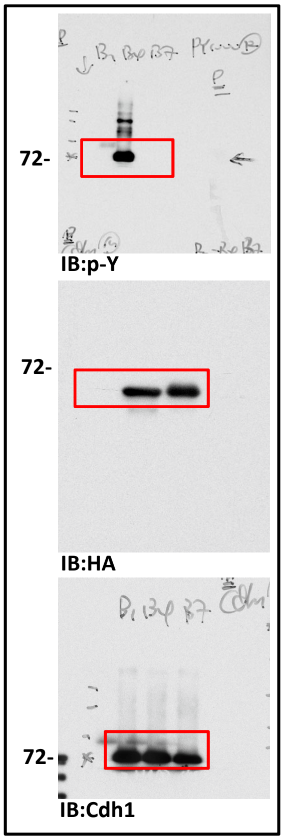

Supplementary Figure 6c

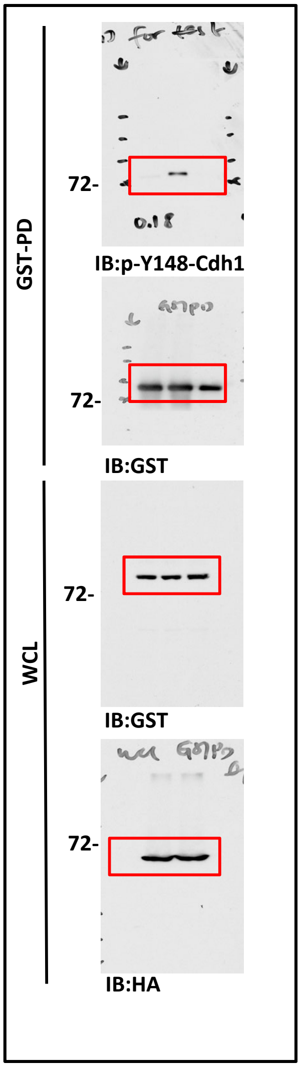

Supplementary Figure 6d

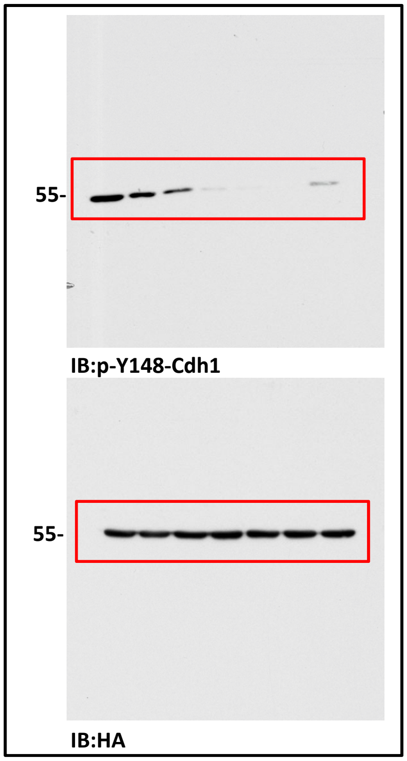

Supplementary Figure 6f

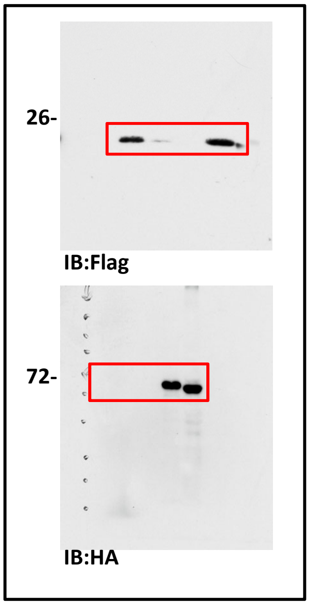

Supplementary Figure 6g

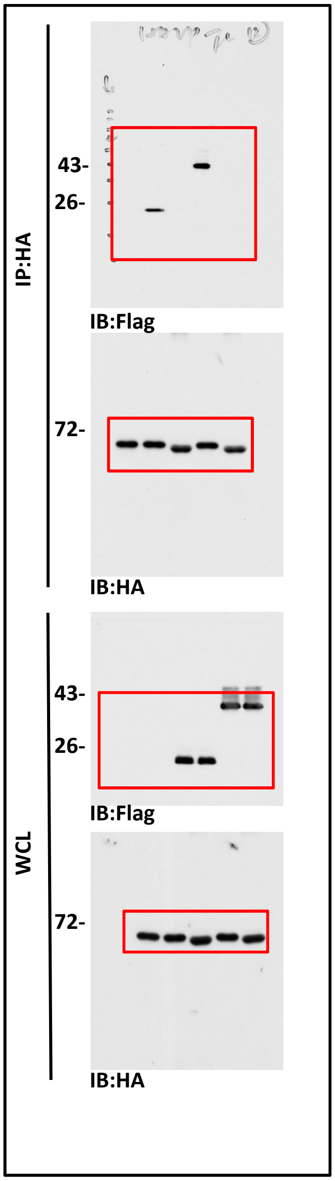

Supplementary Figure 6h

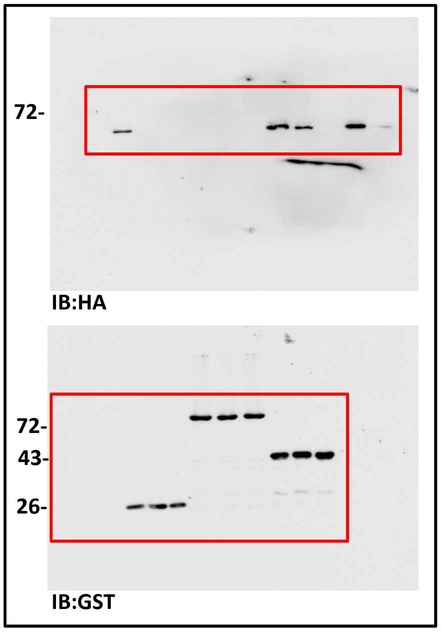

Uncropped immunoblots from Figure 6

Supplementary Figure 6j

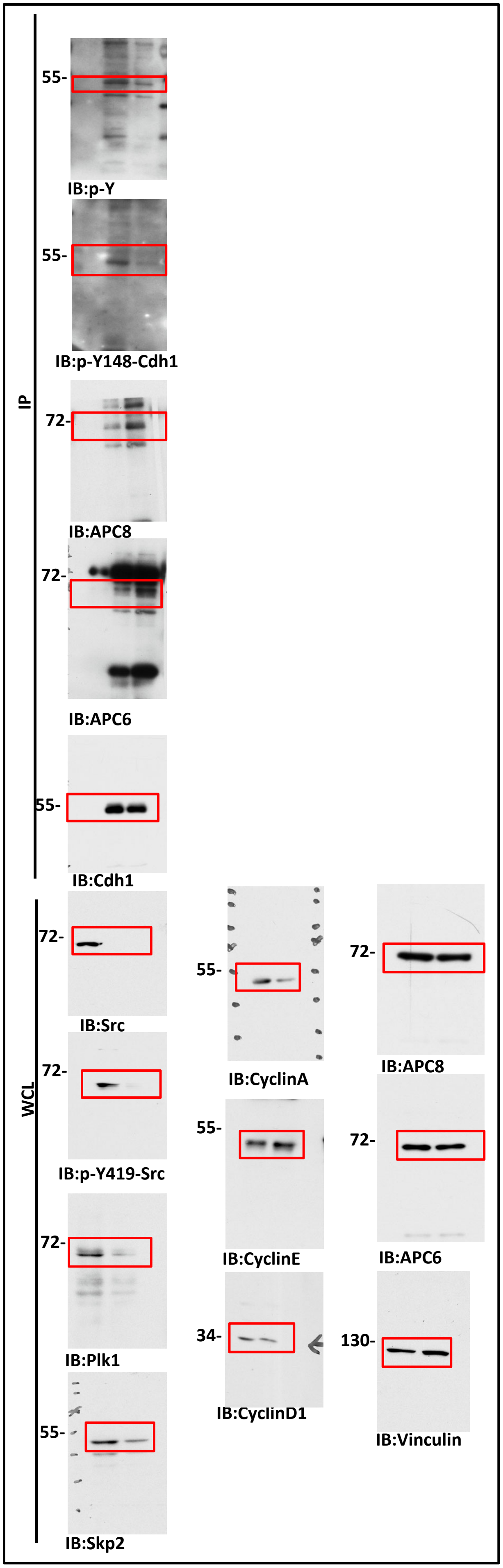

Supplementary Figure 6k

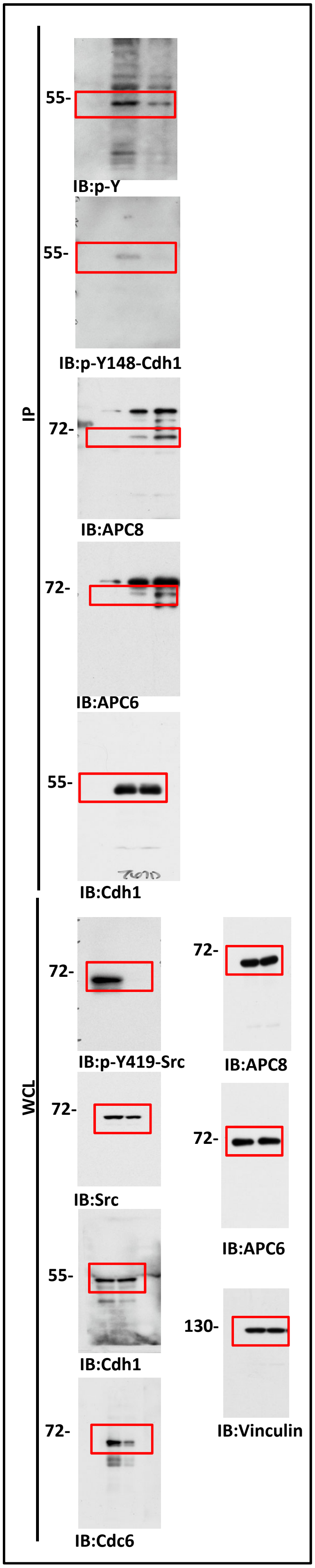

Supplementary Figure 6m

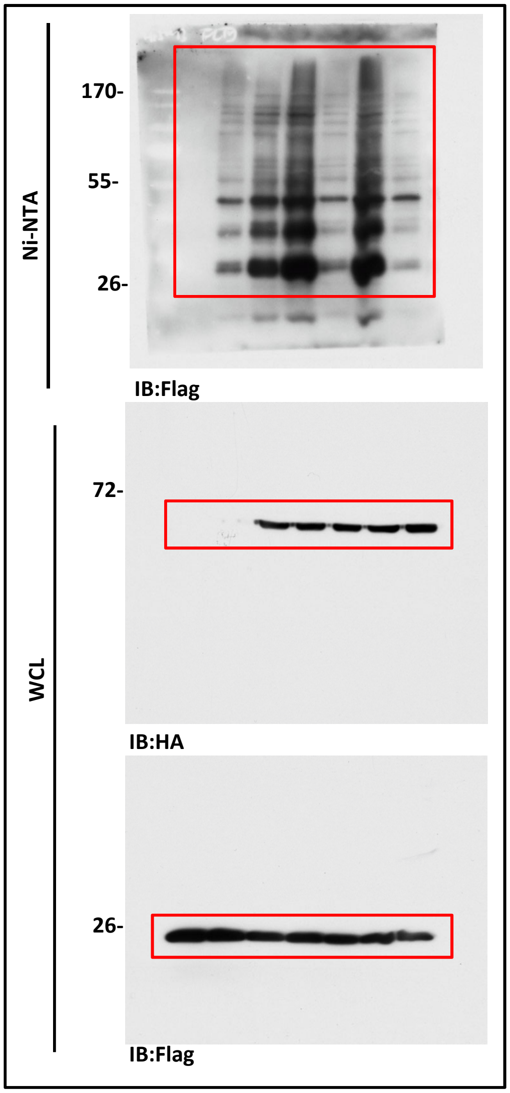

Uncropped immunoblots from Supplementary Figure 6

Supplementary Figure 7d

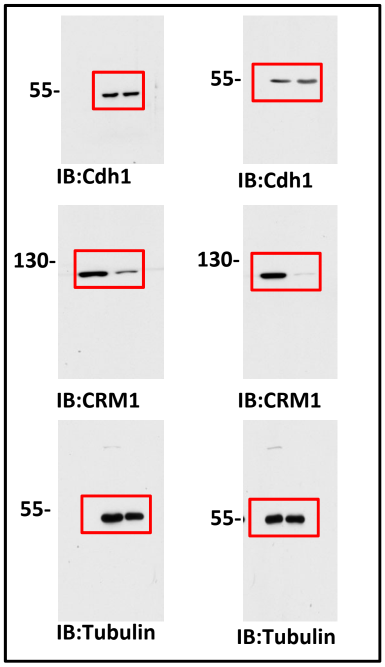

Supplementary Figure 7e

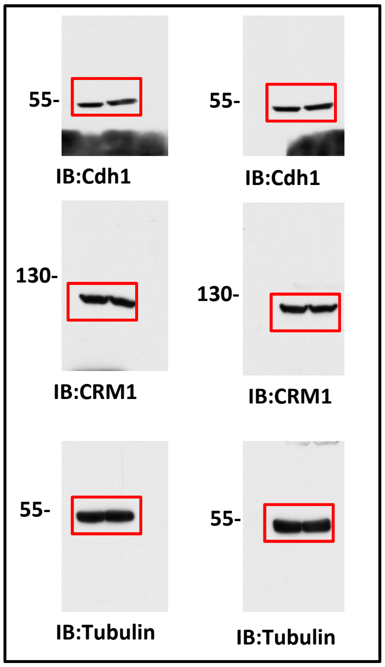

Supplementary Figure 7f

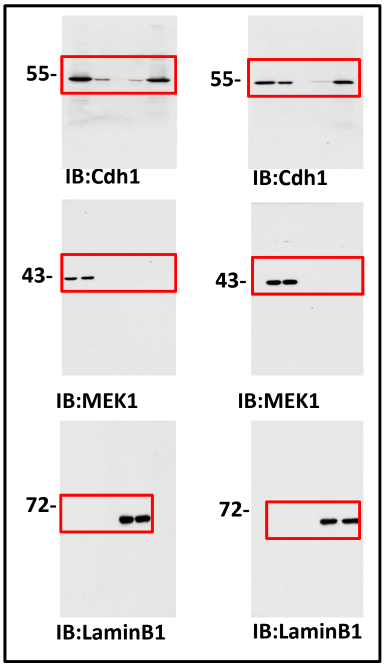

Uncropped immunoblots from Supplementary Figure 7

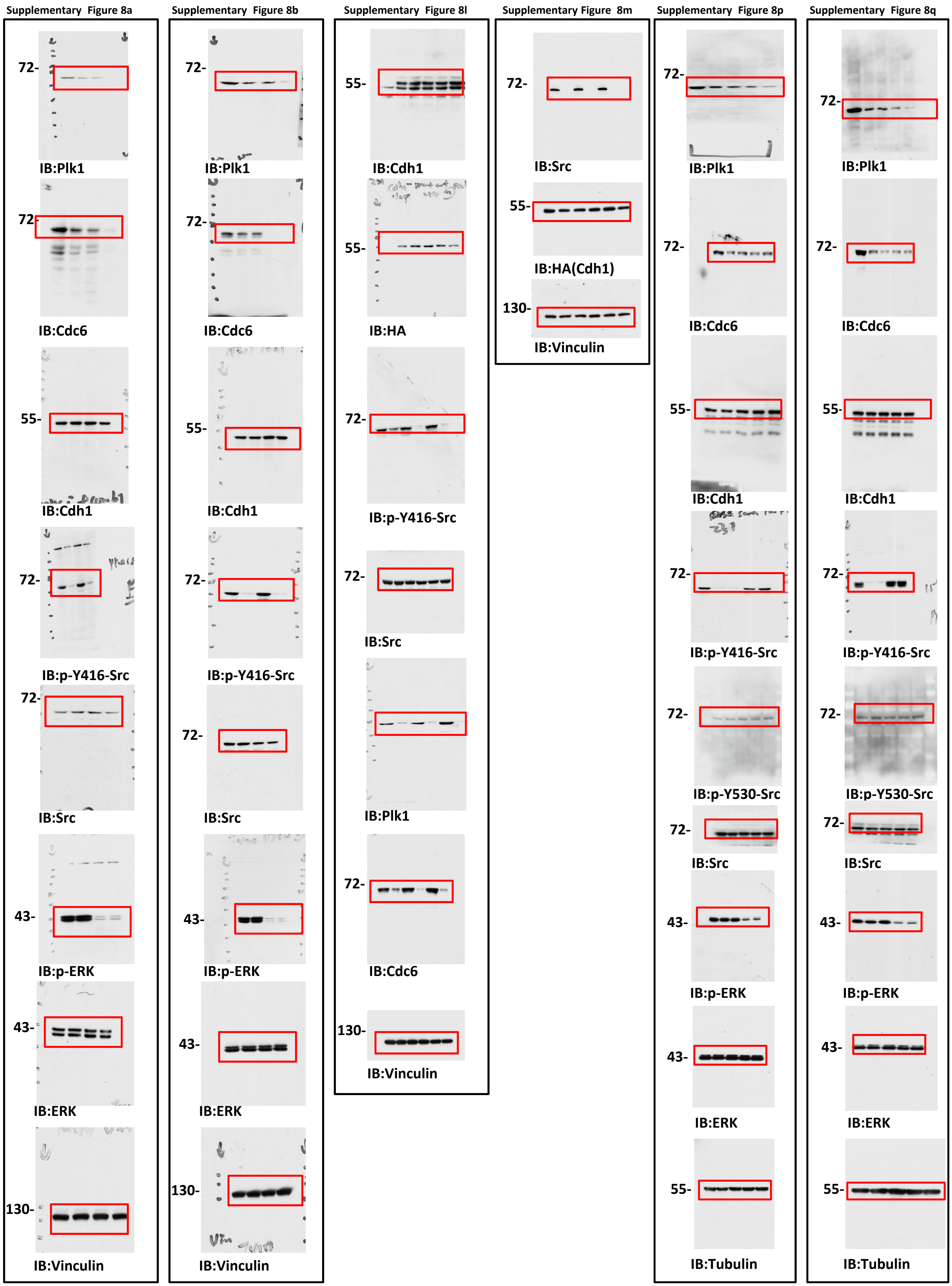

Uncropped immunoblots from Supplementary Figure 8

Supplementary Figure 8t

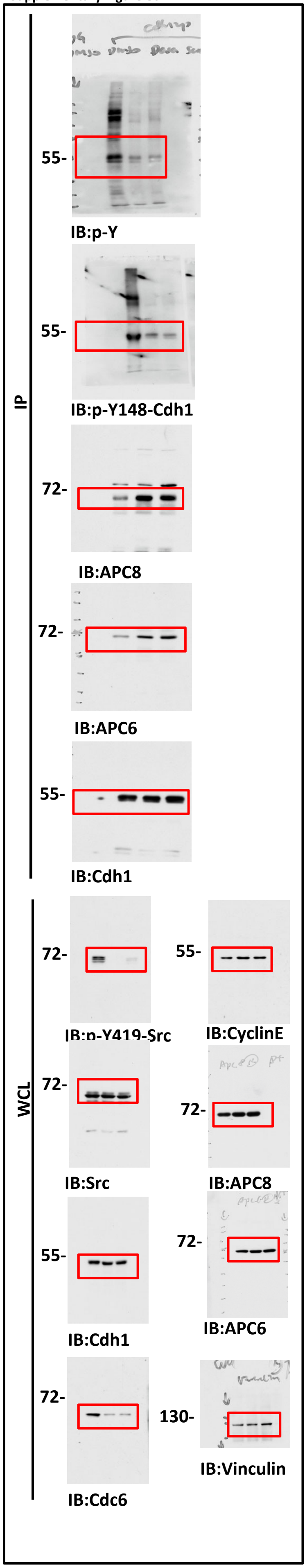

Supplementary Figure 8v

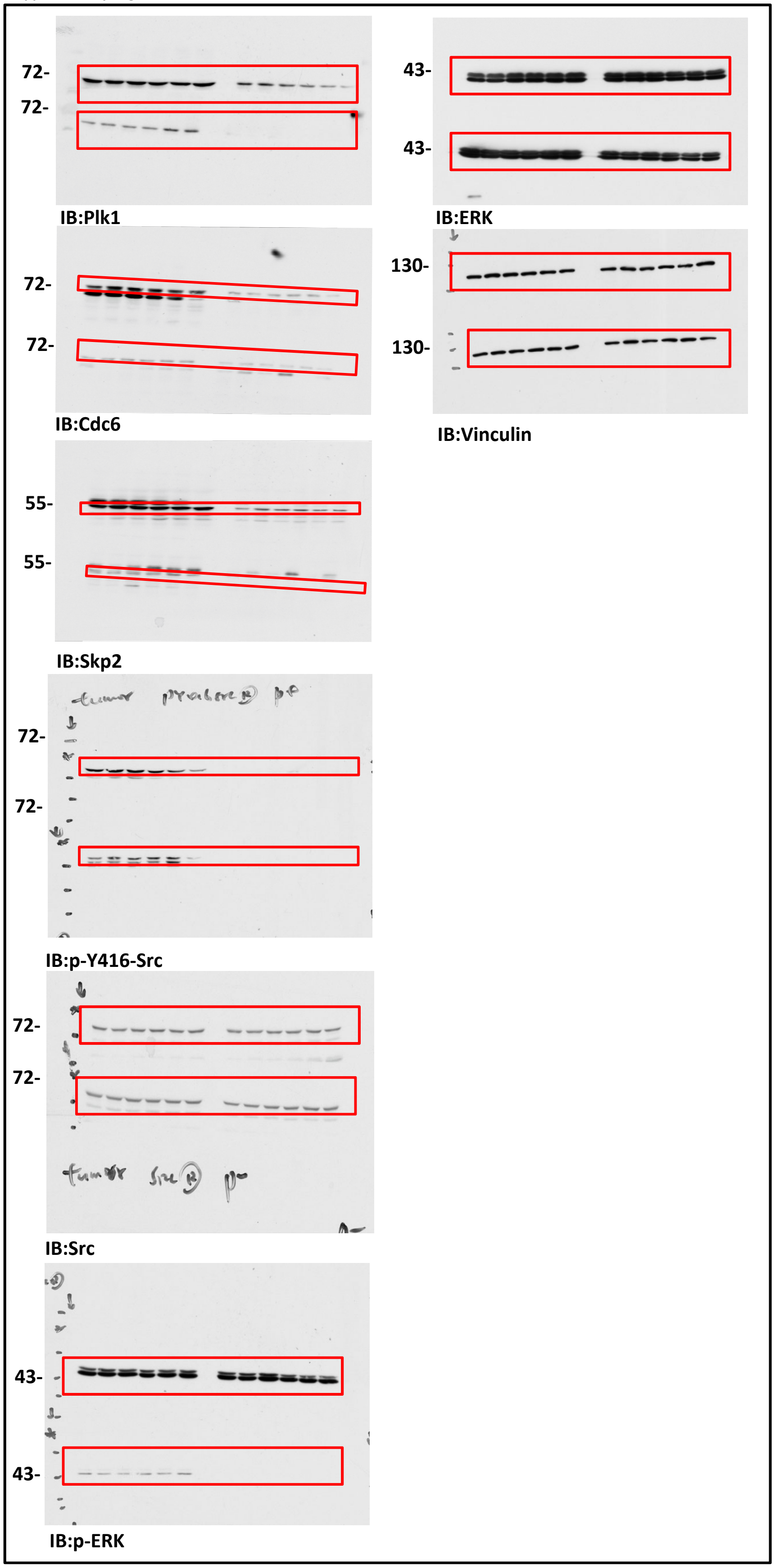

Uncropped immunoblots from Supplementary Figure 8

Supplementary Figure 9a

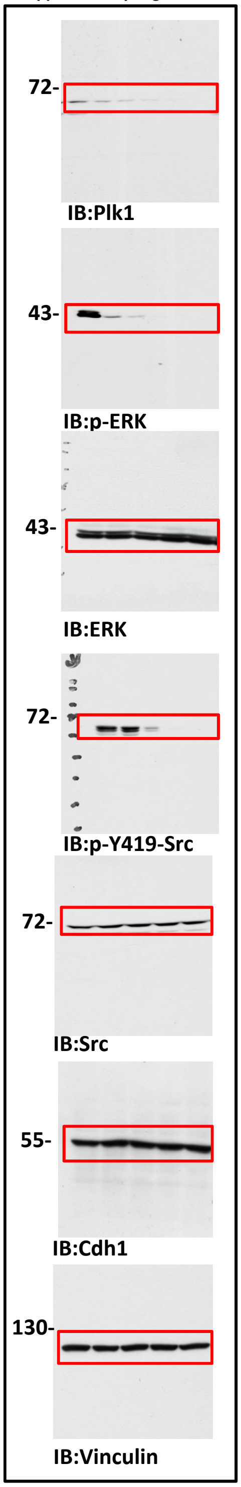

Supplementary Figure 9b

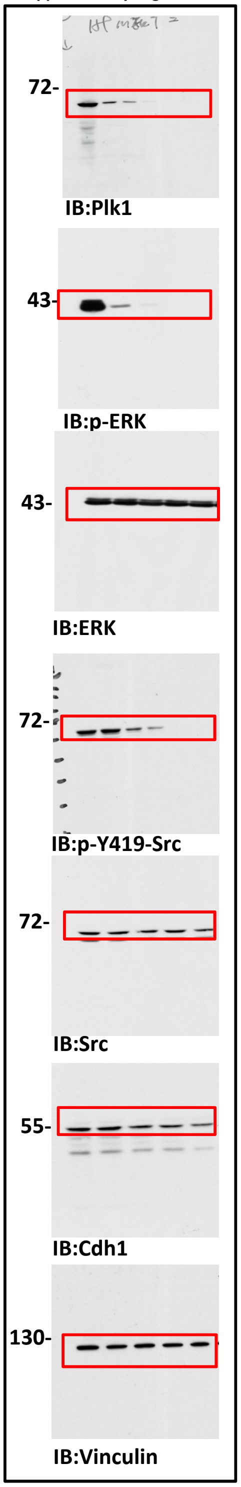

Supplementary Figure 9c

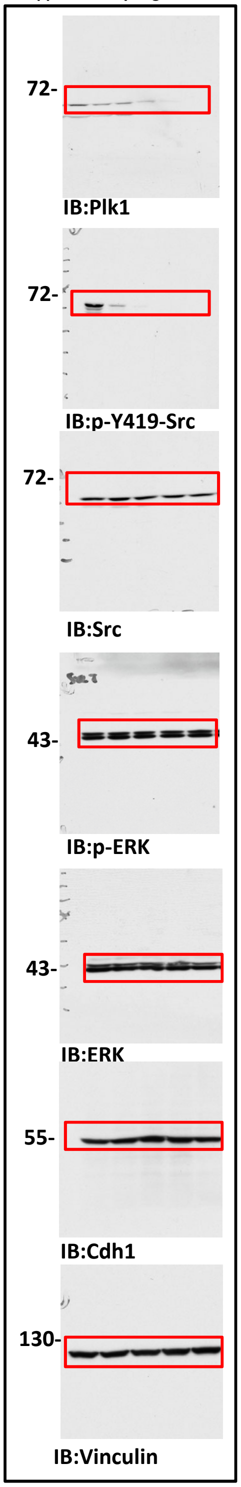

Supplementary Figure 9d

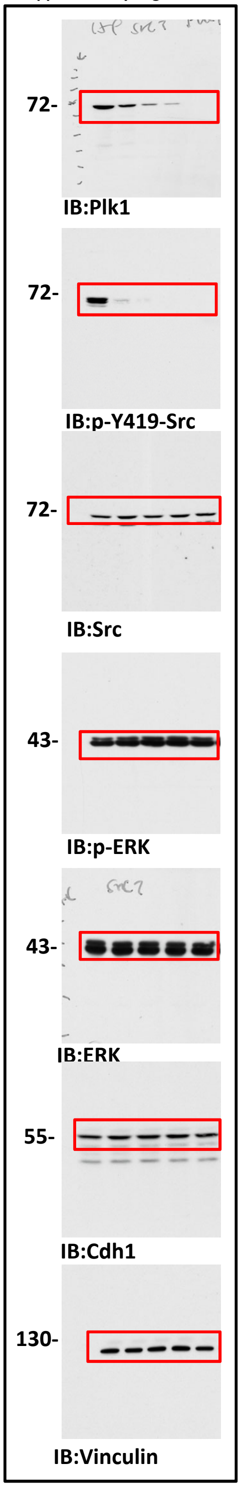

Supplementary Figure 9e

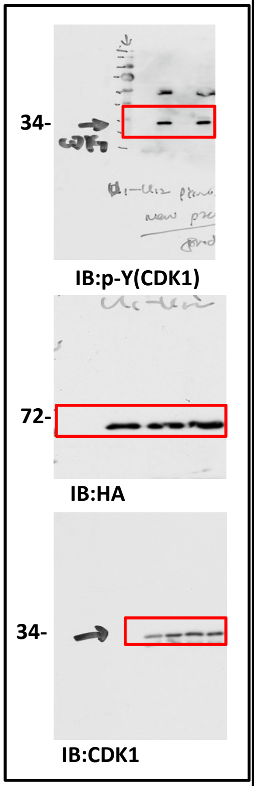

Supplementary Figure 9f

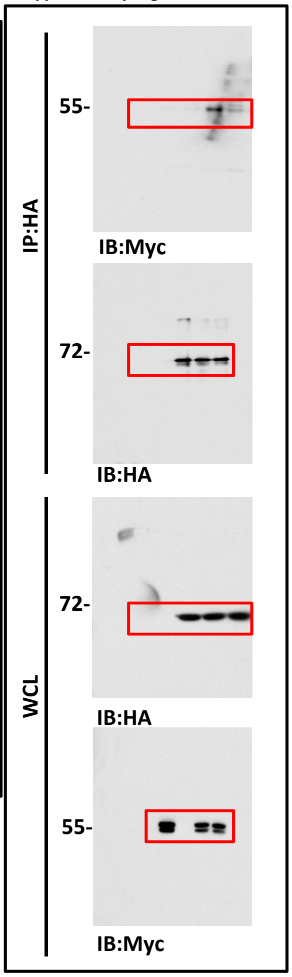

Supplementary Figure 9g

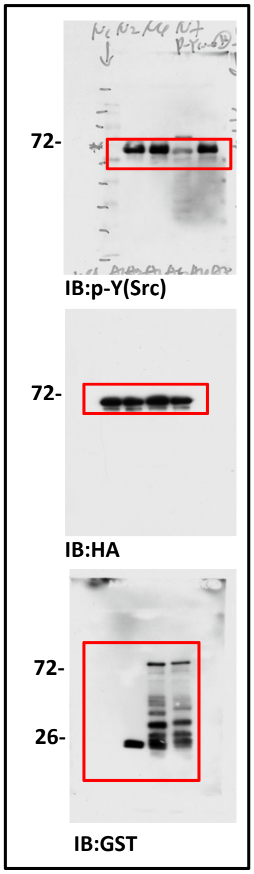

Uncropped immunoblots from Supplementary Figure 9

Supplementary Table 1. A list of primers used in this study

Primer name		Primer sequence (5'-3')
hSrC-HindIII-F	Forward	GCATAAGCTTGGTAGCAACAAGAGCAAGC
hSrC-SalI-R	Reverse	GCATGTCGACCTAGAGGTTCTCCCCGGG
hSrc-K260-BglII-F	Forward	GCATAGATCTAAGGATGCCTGGGAG
hSrc-A259-SalI-R	Reverse	GCATGTCGACTTAGGCCAGGCCCTGAGTC
hSrc-G2A-HindIII-F	Forward	GCATAAGCTTGCTAGCAACAAGAGCAAGC
hSrc-D144-XhoI-R	Reverse	GCATCTCGAGGTCGGAGGGCGCCAC
FYN-HindIII-F	Forward	GCATAAGCTTGGCTGTGTGCAATGTAAG
FYN-SalI-R	Reverse	GCATGTCGACTTACAGGTTTTCACCAG
YES1-NotI-F	Forward	GCATGCGGCCGCGGGCTGCATTAAAAG
YES1-SalI-R	Reverse	GCATGTCGACTTATAAATTTTCTCCTGGCTGG
hSrc-Y530F	Sense	CCACGAGCCCCAGTTCCAGCCCGG
	Antisense	CCGGGCTGGAACTGGGGCTCGGTGG
hSrc-Y419F	Sense	CTCATTGAAGACAATGAGTTCACGGCGCAAG
	Antisense	CTTGCCGCGCCGTGAACTCATTGTCTTCAATGAG
hSrc-K298R	Sense	CAGGGTGGCCATCAGAACCCTGAAGCCTG
	Antisense	CAGGGTGGCCATCAGAACCCTGAAGCCTG
hSrc-S17A	Sense	CAGCGGCGCCGCGCCCTGGAGCCCG
	Antisense	CGGGCTCCAGGGCGCGGCGCCGCTG
hSrc-Dbox1-RLAA	Sense	GATGCCAGCCAGCGGGCCCGCAGCGCCGAGCCCGCCGAGAAC
	Antisense	GTTCTCGGCGGGCTCGGCGCTGCGGGCCCGCTGGCTGGCATC
hSrc-Dbox2-RLAA	Sense	GACGGGAGTCAGAGGCCTTACTGGCCAATGCAGAGAACCC
	Antisense	GGGTTCTCTGCATTGGCCAGTAAGGCCTCTGACTCCCGTC
cHA-hSrc-QPGEEI	Sense	CGTCCACCGAGCCCCAGTACGAGGAAATCGAGAACCTCCTCGAGGGTACC
	Antisense	GGTACCCTCGAGGAGGTTCTCGATTTCCTCGTACTGGGGCTCGGTGGACG
hSrc-R178A	Sense	GGACCTTCCTCGTGGCAGAAAGTGAGACC
	Antisense	GGTCTCACTTTCTGCCACGAGGAAGGTCC
hSrc-W121A-S	Sense	CAACAACACAGAGGGAGACGCCTGGCTGGCCCACTCGCTC
	Antisense	GAGCGAGTGGGCCAGCCAGGCGTCTCCCTCTGTGTTGTTG
hSrc-S17A-S	Sense	AGCGGCGCCGCGCCCTGGAGCCCG
	Antisense	CGGGCTCCAGGGCGCGCGGCCGCT
hSrc-S17D-S	Sense	CAGCGGCGCCGCGACCTGGAGCCCGC
	Antisense	GCGGGCTCCAGGTCGCGGCGCCGCTG
hSrc-dSB-S	Sense	CGGCAAGGTGCCAAATTCGCTGCCCTCTATG
	Antisense	CATAGAGGGCAGCGAATTTGGCACCTTGCCG
$shSrc^{resistant}$	Sense	GTCTAGGACGGAGACAGATCTCTCTTTTAAGAAAGGCGAGCGGCTC
	Antisense	GAGCCGCTCGCCTTTCTTAAAAGAGAGATCTGTCTCCGTCCTAGAC
shhSrc-A	Sense	CCGGGACAGACCTGTCCTTCAAGAACTCGAGTTCTTGAAGGACAGGTCTGTCTTTTTG
	Antisense	AATTCAAAAAGACAGACCTGTCCTTCAAGAACTCGAGTTCTTGAAGGACAGGTCTGTC
sghSrc-1	Sense	CACCGTAACCGCTCTGACTCCCGTC
	Antisense	AAACGACGGGAGTCAGAGCGGTTAC
sghCdh1-3	Sense	CACCGGCAGTACACGGAGCACCTGG
	Antisense	AAACCCAGGTGCTCCGTGTACTGCC
shhCRM1-A	Sense	CCGGGCTCAAGAAGTACTGACACATCTCGAGATGTGTCAGTACTTCTTGAGCTTTTTG
	Antisense	AATTCAAAAAGCTCAAGAAGTACTGACACATCTCGAGATGTGTCAGTACTTCTTGAGC
hCdh1-BamHI-F	Forward	GCATGGATCCGACCAGGACTATGAGC
hCdh1-N(1-174)-SalI-R	Reverse	GCATGTCGACTTAGATCTTGGAGATCTTG
hCdh1-S172-BamHI-F	Forward	GCATGGATCCTCCAAGATCCCCTTC
hCdh1-SalI-R	Reverse	GCATGTCGACTTACCTGATCCTGGTGAAGAGG
mCttn-T323-BamHI-F	Forward	GCATGGATCCACCTTTGAAGAAGTGG
mCttn-XhoI-R	Reverse	GCATCTCGAGCTACTGCCGCAG
hCdh1-Y5F-BamHI-F	Forward	GCATGGATCCGACCAGGACTTTGAGCGGCGCC
hCdh1-Y91F	Sense	GACGGCCTGGCCTTCTCTGCCCTGCTC
	Antisense	GAGCAGGGCAGAGAAGGCCAGGCCGTC
hCdh1-Y130F	Sense	GAAGGGTCTGTTCACGTTTTCCCTTAGCACCAAGC
	Antisense	GCTTGGTGCTAAGGGAAAACGTGAACAGACCCTTC

hCdh1-Y148F	Sense	CGATGTGTCTCCCCTTCTCCCTGTCTCCCG
	Antisense	CGGGAGACAGGGAGAAGGGAGACACATCG
hCdh1-Y189F	Sense	GAGCTGCAGGACGACTTCTTCCTAATCTGGTGGAC
	Antisense	GTCCACCAGATTGAGGAAGAAGTCGTCCTGCAGCTC
hCdh1-Y148E	Sense	CAACGATGTGTCTCCCGAGTCCCTGTCTCCCGTC
	Antisense	GACGGGAGACAGGGACTCGGGAGACACATCGTTG
$shCdh1^{resistant}$	Sense	CCCCGATGACGGCAATGACGTCTCTCCCTACTCCC
	Antisense	GGGAGTAGGGAGAGACGTCATTGCCGTCATCGGGG
hCdh1-dNES	Sense	GACGCGCCCGAGGTGGACTGGTG
	Antisene	CGACCAGTCCACCTCGGGCGCGTC

Supplementary References

1. Brown, N. G. *et al.* ual RING E3 Architectures Regulate Multiubiquitination and Ubiquitin Chain Elongation by APC/C. *Cell* **165**, 1440–1453 (2016).
