# Supplementary material for: Interplay between c-Src and the APC/C co-activator Cdh1 regulates mammary tumorigenesis
Source: Nat Commun. 2019 Aug 16;10:3716. doi: 10.1038/s41467-019-11618-7 (PMC6697746; doi:10.1038/s41467-019-11618-7)
Supplement: Supplementary file 3 — Reporting Summary [file 41467_2019_11618_MOESM3_ESM.pdf]

nature research

Corresponding author(s): Lixin Wan

Last updated by author(s): 6/26/2019

Reporting Summary

Nature Research wishes to improve the reproducibility of the work that we publish. This form provides structure for consistency and transparency in reporting. For further information on Nature Research policies, see Authors & Referees and the Editorial Policy Checklist.

Statistics

For all statistical analyses, confirm that the following items are present in the figure legend, table legend, main text, or Methods section.

n/a	Confirmed	
☐	☒	The exact sample size (n) for each experimental group/condition, given as a discrete number and unit of measurement
☐	☒	A statement on whether measurements were taken from distinct samples or whether the same sample was measured repeatedly
☐	☒	The statistical test(s) used AND whether they are one- or two-sided<br>*Only common tests should be described solely by name; describe more complex techniques in the Methods section.*
☒	☐	A description of all covariates tested
☒	☐	A description of any assumptions or corrections, such as tests of normality and adjustment for multiple comparisons
☐	☒	A full description of the statistical parameters including central tendency (e.g. means) or other basic estimates (e.g. regression coefficient) AND variation (e.g. standard deviation) or associated estimates of uncertainty (e.g. confidence intervals)
☐	☒	For null hypothesis testing, the test statistic (e.g. F, t, r) with confidence intervals, effect sizes, degrees of freedom and P value noted<br>*Give P values as exact values whenever suitable.*
☒	☐	For Bayesian analysis, information on the choice of priors and Markov chain Monte Carlo settings
☒	☐	For hierarchical and complex designs, identification of the appropriate level for tests and full reporting of outcomes
☐	☒	Estimates of effect sizes (e.g. Cohen's d, Pearson's r), indicating how they were calculated

Our web collection on statistics for biologists contains articles on many of the points above.

Software and code

Policy information about availability of computer code

Data collection	*Provide a description of all commercial, open source and custom code used to collect the data in this study, specifying the version used OR state that no software was used.*
Data analysis	Graphpad Prism 7.0, ImageJ, Leica Application Suite X (LAS X), FlowJo

For manuscripts utilizing custom algorithms or software that are central to the research but not yet described in published literature, software must be made available to editors/reviewers. We strongly encourage code deposition in a community repository (e.g. GitHub). See the Nature Research guidelines for submitting code & software for further information.

Data

Policy information about availability of data

All manuscripts must include a data availability statement. This statement should provide the following information, where applicable:

- Accession codes, unique identifiers, or web links for publicly available datasets
- A list of figures that have associated raw data
- A description of any restrictions on data availability

The data that support the findings of this study are available from the corresponding authors in reasonable request.

Field-specific reporting

Please select the one below that is the best fit for your research. If you are not sure, read the appropriate sections before making your selection.

☒ Life sciences　☐ Behavioural & social sciences　☐ Ecological, evolutionary & environmental sciences

Life sciences study design

All studies must disclose on these points even when the disclosure is negative.

Sample size	The chosen sample size are based on the numbers used for previous publications, which is most optimal to generate statistically significant results.
Data exclusions	No data were excluded for statistical analyses.
Replication	All attempts at replication were successful.
Randomization	The samples/cells were randomized to be examined. For in vivo mouse xenograft experiments, the mice were randomly grouped into vehicle and drug-treatment groups prior to the treatment.
Blinding	Blinding was not relevant to the study because all cells/samples were analyzed in the same way.

Reporting for specific materials, systems and methods

We require information from authors about some types of materials, experimental systems and methods used in many studies. Here, indicate whether each material, system or method listed is relevant to your study. If you are not sure if a list item applies to your research, read the appropriate section before selecting a response.

Materials & experimental systems

n/a	Involved in the study	
☐	☒	Antibodies
☐	☒	Eukaryotic cell lines
☒	☐	Palaeontology
☐	☒	Animals and other organisms
☒	☐	Human research participants
☒	☐	Clinical data

Methods

n/a	Involved in the study	
☒	☐	ChIP-seq
☐	☒	Flow cytometry
☒	☐	MRI-based neuroimaging

Antibodies

Antibodies used	Anti-Src (2123, 1:2000), anti-p-Y419-Src (6943, 1:2000), anti-p-Y530-Src (2105, 1:2000), anti-p-S17-Src (5473, 1:2000), anti-p-Y1000 (8954, 1:1000), anti-p-MAPK/CDK Substrates (PXS*P or S*PXR/K) (2325, 1:1000), anti-p-T202/pY204-ERK1/2 (4370, 1:3000), anti-ERK1/2 (4695, 1:3000), anti-p-S217/p-S221-MEK1/2 (9154, 1:3000), anti-MEK1/2 (9122, 1:3000), anti-p-Y705-Stat3 (9145, 1:1000), anti-YAP (14074, 1:1000), anti-CREB (9197, 1:2000), anti-p-S133-CREB (9198, 1:2000), anti-Exportin-1/CRM1 (46249, 1:1000), anti-PDGFR-β (3169, 1:1000), anti-p-Y751-PDGFR-β (4549, 1:1000), anti-Lamin B1 (13435, 1:1000) and anti-GST (2622, 1:2000) antibodies were purchased from Cell Signaling Technology. Anti-cyclin A (H-432, 1:1000), anti-cyclin E (HE-12, 1:1000), anti-cyclin D1 (C-20, 1:1000), anti-STAT3 (F-2, 1:1000), anti-Plk1 (F-8, 1:1000), anti-APC10 (B-1, 1:1000), anti-Cdc6 (180.2, 1:1000), anti-Cdc27 (AF3.1, 1:1000), anti-Cdh1(DCS-266, 1:1000), anti-Cdc20 (E-7, 1:2000), anti-Cdc2 (17, 1:1000), anti-Vinculin (H-10, 1:1000). anti-c-Myc (9E10, 1:2000) and polyclonal anti-HA (Y-11, 1:2000) antibodies were purchased from Santa Cruz. Anti-Tubulin (T-5168, 1:2000) and, anti-APC6 (A301-165A, 1:1000), and anti-APC8 (A301-181A, 1:1000) antibodies were purchased from Bethyl Labs. Polyclonal anti-Flag antibody (F-2425, 1:2000), monoclonal anti-Flag (F-3165, 1:2000) antibody, anti-Flag agarose beads (A-2220), anti-Flag agarose beads (A-2220) anti-HA agarose beads (A-2095) as well as peroxidase-conjugated anti-mouse secondary antibody (A-4416, 1:2000) and peroxidase-conjugated anti-rabbit secondary antibody (A-4914, 1:2000) were purchased from Sigma. Monoclonal anti-SKP2 antibody (32-3300, 1:2000) was purchased from Thermo Fisher Scientific. Anti-p-Y357-YAP (ab62751, 1:500) antibody was purchased from Abcam. Monoclonal anti-HA antibody (MMS-101P, 1:2000) was purchased from Covance. Human anti-centromere antibody (ACA) derived from human CREST patient serum was purchased from Antibodies, Inc. (#15-235-0001, 1:1000). Polyclonal anti-p-Y148-Cdh1(1:200) antibody was generated by Genescript.
Validation	Antibody validation was deferred to the manufacturers and was supported by multiple publications.

Eukaryotic cell lines

Policy information about cell lines

Cell line source(s)	293T, HEK293, MCF7, T47D, HCT116, BT474, MCF10A, SH-SY5Y, Hs587T, and ZR75-1 were obtained from ATCC. MDA-MB-231 and SUM159PT are kind gift from Dr. Andriy Marusyk. Src-/- MEFs are kind gift from Drs. Philippe Soriano and Akira Imamoto. Cdh1-/- MEFs are kind gift from Dr. Marcos Malumbres 12. Immortalized human ovarian epithelial cells are kind gift from Drs. Hidetaka Katabuchi and Tohru Kiyono. Immortalized human foreskin fibroblasts are kind gift from Dr. Wenyi Wei.

Authentication

Cancer cell lines were used without further authentication.

Mycoplasma contamination

293T, MCF7, T47D, MDA-MB-231 and SUM159PT cell lines were tested negative for mycoplasma contamination.

Commonly misidentified lines
(See ICLAC register)

No commonly misidentified cell lines were used in this study.

Animals and other organisms

Policy information about studies involving animals; ARRIVE guidelines recommended for reporting animal research

Laboratory animals

6-week-old female nude mice (NCRNU-M-M) were purchased from Taconic.

Wild animals

The study did not involve wild animals.

Field-collected samples

The study did not involve samples collected from the field.

Ethics oversight

All research involving animals was complied with protocols approved by the University of South Florida Animal Care and Use Committee.

Note that full information on the approval of the study protocol must also be provided in the manuscript.

Flow Cytometry

Plots

Confirm that:

☐ The axis labels state the marker and fluorochrome used (e.g. CD4-FITC).

☐ The axis scales are clearly visible. Include numbers along axes only for bottom left plot of group (a 'group' is an analysis of identical markers).

☐ All plots are contour plots with outliers or pseudocolor plots.

☐ A numerical value for number of cells or percentage (with statistics) is provided.

Methodology

Sample preparation

Cells were trypsinized and re-suspended in 200 µl cold PBS, 5 ml of cold 90% ethanol was added for fixation overnight. Prior to the assay, cells were centrifuged for 5 min at 200 × g and re-suspended in 0.5 ml PBS with propidium iodide (PI, 50 µg·ml-1, Sigma) and RNase A (250 µg·ml-1, Roche). After incubating 30 min at 37 °C

Instrument

BD LSR II flow cytometer

Software

FlowJo

Cell population abundance

approximately 1 million

Gating strategy

N/A

☐ Tick this box to confirm that a figure exemplifying the gating strategy is provided in the Supplementary Information.
